# Physical activity and mental health: a systematic review and best-evidence synthesis of mediation and moderation studies

**DOI:** 10.1186/s12966-024-01676-6

**Published:** 2024-11-28

**Authors:** Rhiannon L. White, Stewart Vella, Stuart Biddle, Jordan Sutcliffe, Justin M. Guagliano, Riaz Uddin, Alice Burgin, Maria Apostolopoulos, Tatiana Nguyen, Carmen Young, Nicole Taylor, Samantha Lilley, Megan Teychenne

**Affiliations:** 1 School of Health Sciences, Western Sydney University, Locked Bag 1797, Penrith, NSW 2751 Australia; 2 Global Alliance of Mental Health and Sport, University of Wollongong, Wollongong, Australia; 3 Centre for Health Research, University of Southern Queensland, Springfield Central, QLD 4300 Australia; 4 Faculty of Sport & Health Sciences, University of Jyväskylä, Jyväskylä, FI-40014 Finland; 5 Department of Military Psychology and Leadership, Royal Military College of Canada, Kingston, Canada; 6 Institute for Physical Activity and Nutrition (IPAN) School of Exercise and Nutrition Sciences, Deakin University, Geelong, Australia; 7 School of Exercise and Nutrition Sciences, Deakin University, Burwood, VIC Australia

**Keywords:** Exercise, Depression, Anxiety, Mediators, Moderators, Mechanisms, Wellbeing

## Abstract

**Background:**

While evidence consistently demonstrates that physical activity is beneficial to mental health, it remains relatively unknown how physical activity benefits mental health, and which factors influence the effect of physical activity on mental health. This understanding could vastly increase our capacity to design, recommend, and prescribe physical activity in more optimal ways. The purpose of this study was to systematically review and synthesise evidence of all mediators and moderators of the relationship between physical activity and mental health.

**Methods:**

Systematic searches of four databases (i.e., Scopus, PsycINFO, PubMed, and SPORTDiscus) identified 11,633 initial studies. Empirical studies that quantitatively assessed physical activity, or conducted a physical activity intervention, measured a mental health outcome, and tested one or more mediator or moderator of the relationship between physical activity and mental health were included. A total of 247 met the inclusion criteria; 173 studies examined mediation and 82 examined moderation.

**Results:**

Results of the best-evidence synthesis revealed strong evidence for 12 mediators including affect, mental health and wellbeing, self-esteem, self-efficacy, physical self-worth, body image satisfaction, resilience, social support, social connection, physical health, pain, and fatigue. Moderate evidence was identified for a further 15 mediators and eight moderators.

**Conclusions:**

Findings should inform the design of future physical activity interventions to ensure optimal effects on mental health related outcomes. Additionally, if health professionals were to take these mediators and moderators into consideration when prescribing or recommending physical activity, physical activity would likely have a greater impact on population mental health.

**Supplementary Information:**

The online version contains supplementary material available at 10.1186/s12966-024-01676-6.

## Background

Mental health disorders cause considerable burden of disease globally [1]. As such, the prevention and treatment of mental health disorders remains a high priority worldwide, as does the need to protect and promote positive mental health and wellbeing [2, 3]. Abundant evidence demonstrates that physical activity (PA) has the potential to offer a plethora of psychological benefits. As such, PA has become a universally accepted intervention for both the promotion of mental health and wellbeing, and the prevention of mental ill-health [4]. Despite this, evidence shows that not all PA is equal in terms of its effect on mental health (i.e., the strength and direction of the effect of PA on mental health outcomes varies considerably) [5]. Previous research aiming to understand this heterogeneity has focused on determining the ideal frequency, duration, and intensity of PA, but researchers have not been able to confirm an optimal dose for mental health benefits [6].

While many biophysical and psychosocial mechanisms of the effect of PA on mental health have been proposed [7, 8], it remains relatively unknown how PA contributes to positive mental health outcomes [9]. In-depth understanding of mediators can help to understand real-world mechanisms of influence [10], thereby identifying important factors that should be targeted in PA interventions, programs, and prescriptions, to ensure psychological benefits, or to maximise and strengthen benefits [11]. Indeed, many mediation studies have been conducted over the years, providing evidence of possible pathways between PA and, depression [12], anxiety [13], and psychological wellbeing [14]. However, these studies examine a variety of PA domains (e.g., leisure-time PA, active travel, physical education. occupational activity), mental health outcomes (e.g., depression, anxiety, wellbeing, life satisfaction), and mediators (e.g., self-esteem, fitness). This variability makes it difficult for researchers and practitioners to use mediation evidence to guide intervention development and mental health practice. Both Paluska [15] and Kandola [8] have conducted reviews where multiple proposed mechanisms are discussed and evidence on such mechanisms are presented. However, neither of these reviews were systematic reviews. While both papers advance our knowledge of possible mechanisms, these studies do not comprehensively discuss evidence of all tested mediators. Additionally, the review by Knadola et al. only discussed mechanisms of the effect of PA on depression. However, evidence has shown that PA is beneficial for the prevention of mental ill-health and the promotion of mental health [5]. While Paluska and Schwenk discussed mechanisms for a broader range of psychological outcomes, their paper was published in 2000 and far more mediation studies have occurred since 2000, than prior to 2000. Unlike Paluska and Kadola, Lubans et al. [7] conducted a systematic review of mechanisms. However, this study only included children, and only included experimental studies. While experimental studies offer higher level evidence than observational studies, previously relying on experimental evidence only, has resulted in systematic reviews where it is not possible to confirm which factors are most important to the mental health benefits of PA [16]. In addition to this, observational studies are more likely to test moderation and mediation. Including evidence from a range of study designs would vastly increase the evidence base in which conclusions can be drawn from.

While mediation studies provide valuable evidence of factors that explain the positive effects of PA on mental health, recent research suggests that a range of other factors (i.e., moderators) might influence the strength of the effect of PA on mental health [17, 18]. Moderation analyses are therefore equally important because this evidence provides clinicians and practitioners (e.g., psychologists, exercise physiologists) with more detailed guidance around when, where, and with whom PA may be most beneficial for mental health. However, no study has systematically reviewed all moderators of the relationship between PA and mental health. Therefore, the aim of this systematic review was to combine and synthesise evidence of all mediators and moderators in the association between PA and mental health.

## Methods

This research was conducted in accordance with the Preferred Reporting Items for Systematic Reviews and Meta-Analysis (PRISMA) statement [19] and the PERSiST (implementing Prisma in Exercise, Rehabilitation, Sport medicine and SporTs science) guidelines [20].

### Eligibility criteria

#### Inclusion and exclusion criteria

This review aimed to include evidence of both mental health and mental ill-health. Positive outcome measures included mental health, mental wellbeing, and psychological wellbeing, as well as positive affect and life satisfaction as two core components of mental wellbeing [21]. Mental ill-health is defined as a broad term encompassing mental health disorders and preclinical mental health problems [22–25]. Therefore, negative outcome measures included psychological distress, stress, and negative affect, as well as depression (or depressive symptoms) and anxiety (or symptoms of anxiety), as these are the most common mental health disorders globally [26]. We did not specifically exclude any population group, as such, all populations and participants were able to be included. The full inclusion and exclusion criteria are shown in Table 1.

**Table 1:** Inclusion and exclusion criteria

**Inclusion criteria**	
1	A quantitative assessment of physical activity, or an experimental study where the intervention was a PA intervention, and the intervention group was compared to a control group that did not undertake exercise
2	A quantitative assessment of at least one mental health outcome variable (i.e., mental health, mental wellbeing, psychological wellbeing, subjective wellbeing, life satisfaction, positive affect, negative affect, depression, anxiety, stress, or psychological distress)
3	A quantitative assessment of the relationships between physical activity, mental health, and at least one mediator or moderator
4	A cross-sectional, longitudinal, or experimental design
5	A full-text, peer reviewed journal article
**Exclusion criteria**	
1	measured sedentary behaviour but not levels of physical activity
2	measured mental health specifically in terms of a particular setting or circumstance (e.g., job strain)
3	solely reported qualitative data
4	published in languages other than English

### Search strategy

We searched the following four databases from inception to July 2024: Scopus, PsycINFO, PubMed, and SPORTDiscus. Searches included keywords from three main groups, including mental health terms, physical activity terms, and terms reflecting mediation or moderation. The full search terms can be found in Additional File 1.

### Study selection

After exporting records into Covidence, duplicate records were removed. Two reviewers then independently assessed each title and abstract, and we removed records recommended for exclusion by both reviewers. Two reviewers then independently assessed each of the remaining full text studies, recommending each for inclusion or exclusion. We removed studies recommended for exclusion by both reviewers, and rereviewed those where a discrepancy was present until consensus was reached.

### Data extraction

Two reviewers extracted data from each included article into a pre-defined data extraction table, and a third reviewer checked the extracted data for accuracy. We extracted the numerical and textual results of mediation and moderation analyses in whichever format reported in the original study. We also extracted the year of publication, study design, sample characteristics (i.e., size, age, sex, and country), measure of physical activity and mental health, and what the mediator and/or moderator of interest was and how it was measured. All extracted data can be seen Additional File 4.

### Study quality assessment

We used Risk of Bias criteria developed in a previous systematic review on a similar topic [5] to assess the quality of included studies. This risk of bias criteria was particularly valuable in this study as it was designed to assess quality across a range of study types including experimental and observational studies [5]. However, we needed to assess quality of the studies relative to mediation and moderation analyses, therefore we looked to relevant criterion from mediation study checklists developed by Lubans, Foster [27], Rhodes and Pfaeffli [28], and Cerin, Barnett [29] for an additional item to assess quality for mediation studies.. The full risk of bias criteria is listed in Table 2 and assesses quality across a range of study designs. Two reviewers independently assigned a 1 (present and explicitly explained) or a 0 (absent or inadequately described) for each study on the following seven criteria. We then categorised each study as high (a maximum of one criteria not met = score of 6 or 7), acceptable (most criteria met = score of 4 or 5), or low quality (some criteria met = score of ≤ 3) based on the total score [30].

**Table 2:** Risk of bias criteria

	**Risk of bias criteria**
1	Participant eligibility criteria clearly stated and adequately described, and appropriate to the aims of the research.
2	Power calculation reported, and the study was adequately powered to detect mediation or moderation.
3	Valid measure of physical activity used. Objective measures of PA, self-report measures where the validity and reliability of the measures is cited, or experimental studies whereby participants are allocated to a PA intervention relative to a control condition are all considered a valid assessment of PA.
4	Valid measure used for the mental health outcome variable, or psychometric characteristics of the outcome variable reported and within accepted ranges (e.g., Cronbach’s alpha and test–retest reliability > .60)?
5	Valid measure used to assess the mediator or moderator variable, or psychometric characteristics reported and within accepted ranges (e.g., Cronbach’s alpha and test–retest reliability > .60)?
6	Statistically appropriate/acceptable methods of data analysis used for moderation or mediation.
7	Confounding factors/covariates adjusted for in analyses (e.g., sex, age, weight status).

## Data synthesis

Given the broad variety of mediators and moderators examined in the included studies, meta-analyses were not possible as combining dissimilar studies statistically would not provide meaningful results [31]. Instead, we conducted a best-evidence synthesis. A best-evidence synthesis enables the reviewers to synthesise the results of the individual studies in relation to the certainty of those results. Rather than combining effects for different mediators and moderators that were measured differently, and among diverse populations, the reviewers made sense of the best available evidence for each mediator and moderator, in order to provide meaningful and clinically relevant findings that represent the literature [31]. Certainty of evidence was determined by three factors: the number of studies, the quality of the studies (risk of bias), and the consistency of findings. The evidence criteria for the best-evidence synthesis were adapted from previous studies and can be found in Table 3 [30, 32, 33].

**Table 3:** Evidence criteria for the best-evidence synthesis

Evidence category	Criteria
Very strong evidence	Four or more high quality studies, where ≥ 80% of the findings consistently support moderation or mediation
Strong evidence	Four or more acceptable quality studies, where ≥ 80% of the findings consistently support moderation or mediation
Moderate evidence	Two or more high/acceptable studies, where ≥ 60% of the findings consistently support moderation or mediation
	OR
	Two or more low quality studies, where ≥ 80% of the findings consistently support moderation or mediation
Limited evidence	Evidence provided by one study, or by all low-quality studies
Conflicting evidence	Inconsistent findings across multiple studies (< 60% of studies report consistent findings)
No evidence	No studies support mediation or moderation regardless of the number or quality of studies

While not combining effects statistically, to synthesise the results in a meaningful way and provide overarching conclusions based on evidence, we needed to group similar mediators and moderators. We used existing literature on mechanisms and contextual factors to create the following broad categories: psychological, social, behavioural, physiological, neurobiological, cognitive, environmental, and individual characteristics [7, 8, 15, 16]. Given the breadth and number of psychological mediators and moderators, we also grouped more similar variables together under subheadings to assist interpretation.

## Results

**Fig. 1:**
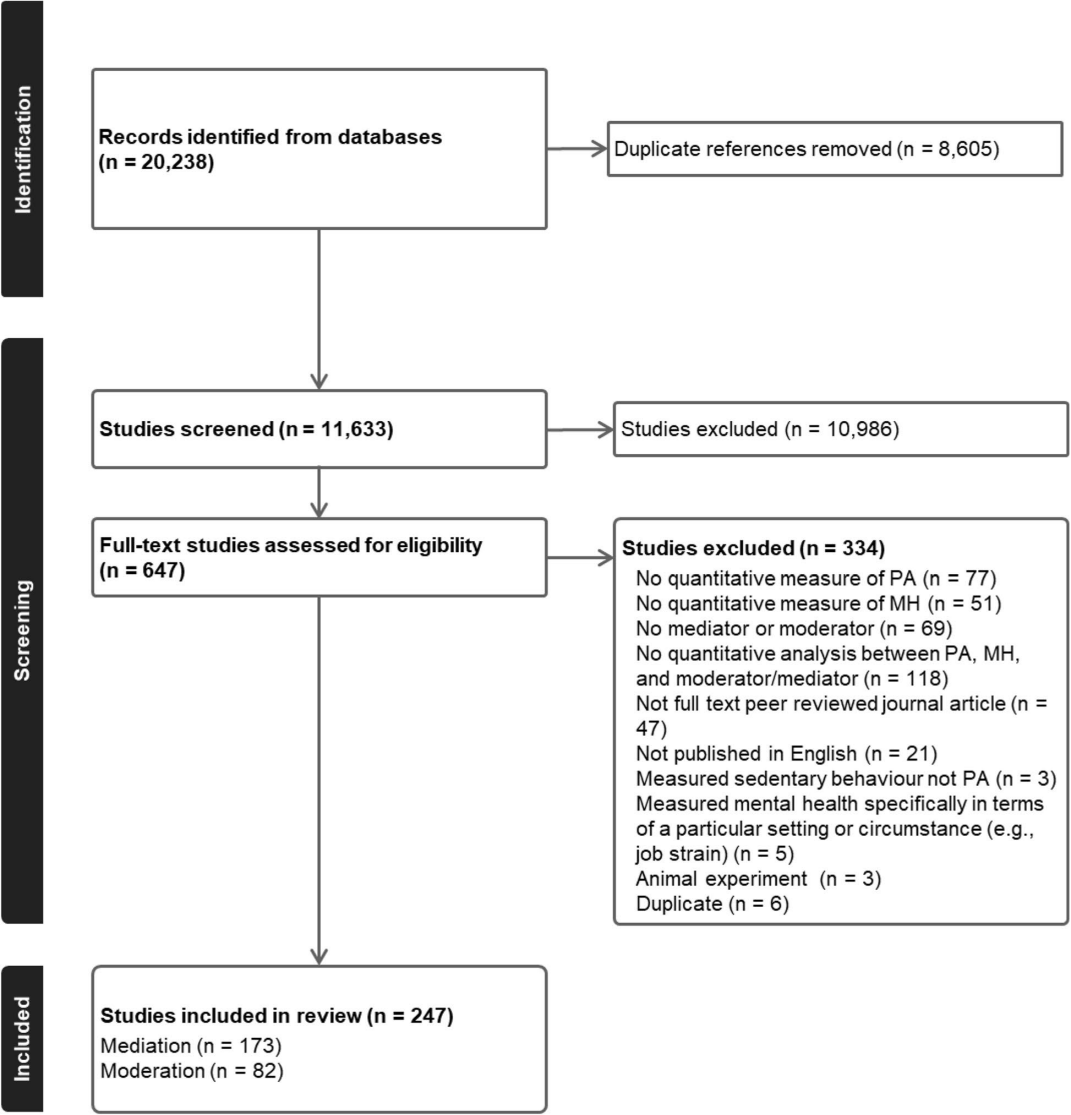
PRISMA flow diagram of studies

Searches yielded 20,238 records, with 11,633 unique articles once duplicates were removed (Fig. 1). Following title and abstract screening, we reviewed 647 full-text articles. A total of 247 studies met the inclusion criteria [12, 13, 34–278], where 173 studies examined mediation and 82 examined moderation (a list of all included studies is available in Additional File 2). Publication dates ranged from 1986–2024, with 61% being published in the last 5 years. A total of 39 experimental studies, and 11 daily/weekly diary or ecological momentary assessment studies were included. Most studies were observational (80%) with 165 cross-sectional, 1 prospective, and 31 longitudinal. The sample sizes ranged from 12 to 727,865, with a total sample size of 2,436,311. Most studies also included general population participants, with 199 studies recruiting adults (81%), 35 studies recruiting adolescents (14%), and 11 studies recruiting children (4%). Of the 199 studies recruiting adults, 49 studies recruited College or University students (25%) and 24 studies recruited older adults (12%). A total of 34 studies (14%) specifically recruited clinical populations with a health or medical condition, including cancer (*n* = 7), depression (*n* = 5), multiple sclerosis (*n* = 3), and spinal cord injury (*n* = 3).

The most assessed outcome was depression or depressive symptoms, followed by psychological distress, life satisfaction, and anxiety. Twenty-three studies measured PA in terms of allocation to an exercise intervention group or to the control group and 20 studies measured PA with a device (e.g., accelerometer). All other studies used self-report measures. The included studies also assessed a wide range of PA types, domains, and contexts. For example, of the 612 results extracted, 101 represent leisure-time PA, 15 active travel, 11 household/domestic PA, and 11 occupational PA. Only four results reflect physical education, three school sport, and a further five school PA. A total of 34 results reflects sport participation, with other results reflecting walking (*n* = 16), running (*n* = 10), yoga (*n* = 5), Tai Chi (*n* = 2), swimming (*n* = 2), cycling (*n* = 1), dancing (*n* = 2), and Ba duan Jin (*n* = 2), and nature-based PA (*n* = 7). Nine results specifically reflected aerobic exercise and six resistance training. All other results reflect an assessment of total PA. The study characteristics of each study are included in Additional File 3.

### Risk of bias

Initial agreement between the two reviewers was 80%, and the kappa coefficient (κ = 0.57) indicated moderate agreement [279]. Upon discussion, 100% agreement was achieved. Of the included studies, 25.5% percent of studies were of high quality, 64.0% were acceptable, and 10.5% were of low quality. The complete results are included in Additional File 5.

### Best-evidence synthesis

The full results from each included study are presented in Additional File 4. This file includes the specific mediation or moderation results of each study relative to the specific population, outcome, and assessment of physical activity. Those interested in the in-depth results relative to a specific mediator (e.g., resilience) can refer to Additional File 4 to see all results for the specific mediator, including the studies, populations, and measures that results are based upon.

Tables 4 and 5 provide a summary of these findings grouped under higher-level mediator and moderator categories. These tables include all data extracted from the 247 included studies regardless of study design or study quality, to best summarise the entire evidence base, however, the Additional Files provide more detailed granular results. The text-based results reported below use the six best-evidence synthesis categories (i.e., very strong, strong, moderate, limited, conflicting, and no evidence) to draw attention to mediators and moderators with strong or moderate evidence. As a summary, Table 6 lists all significant mediators and moderators.

**Table 4:**
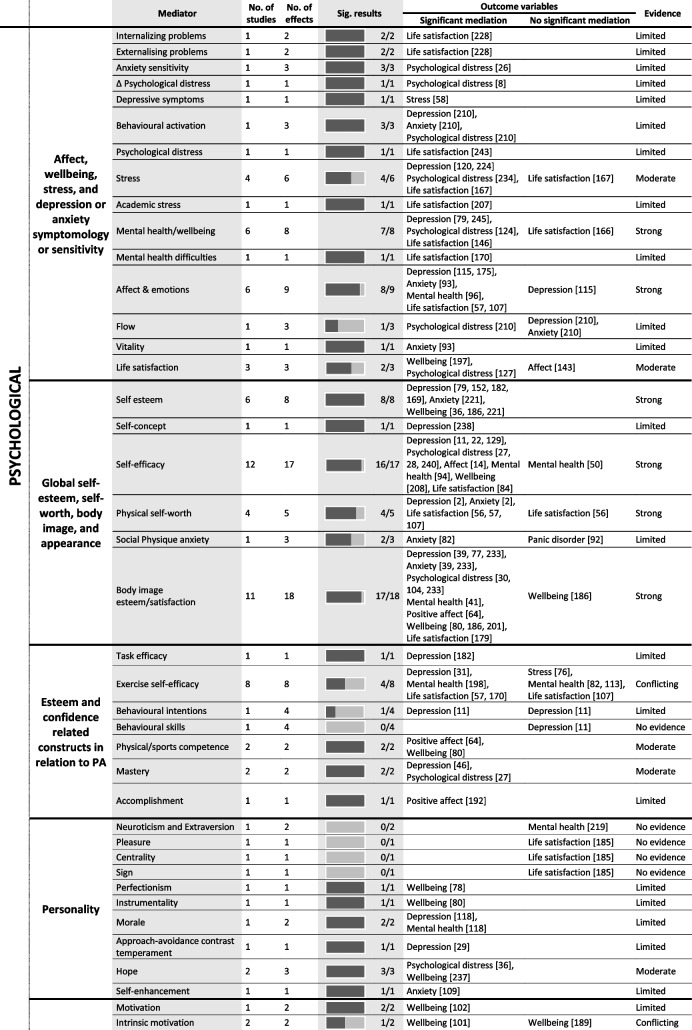

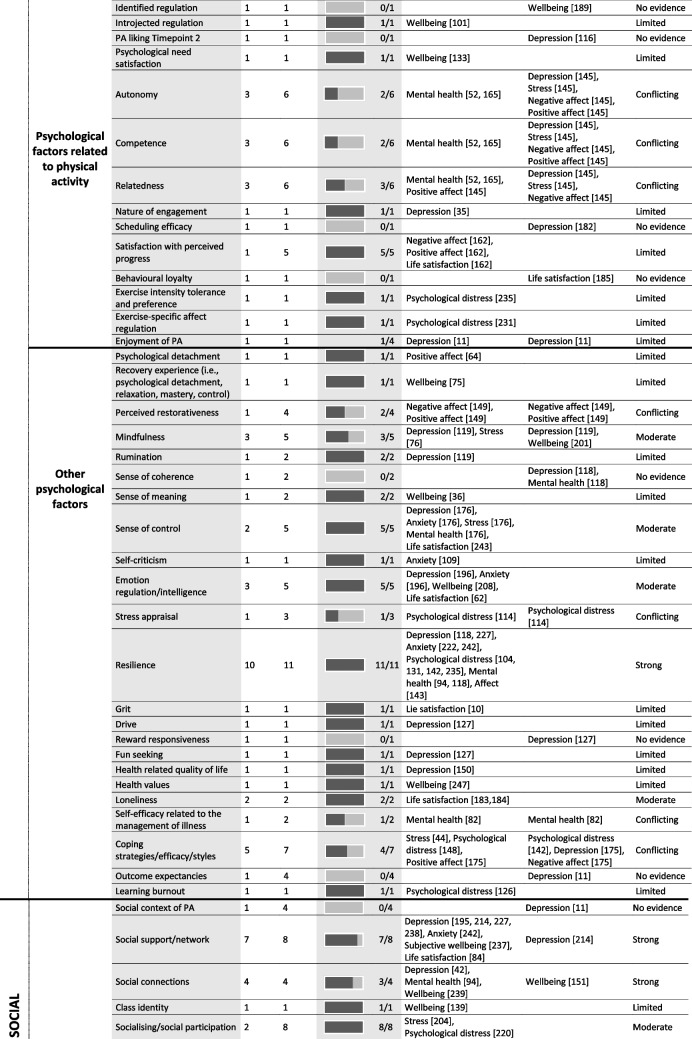

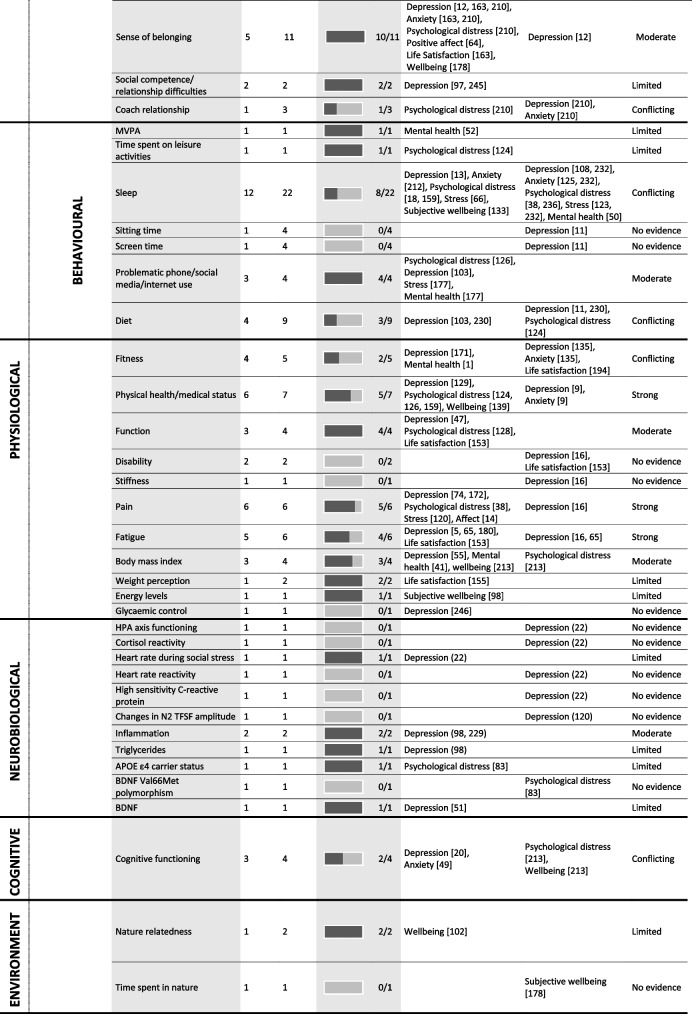
Summary of significant mediators in the association between physical activity and mental health

*PA* physical activity; the numbers in parentheses in the ‘outcome variable’ column refer to the individual study IDs of the 247 included studies

**Table 5:**
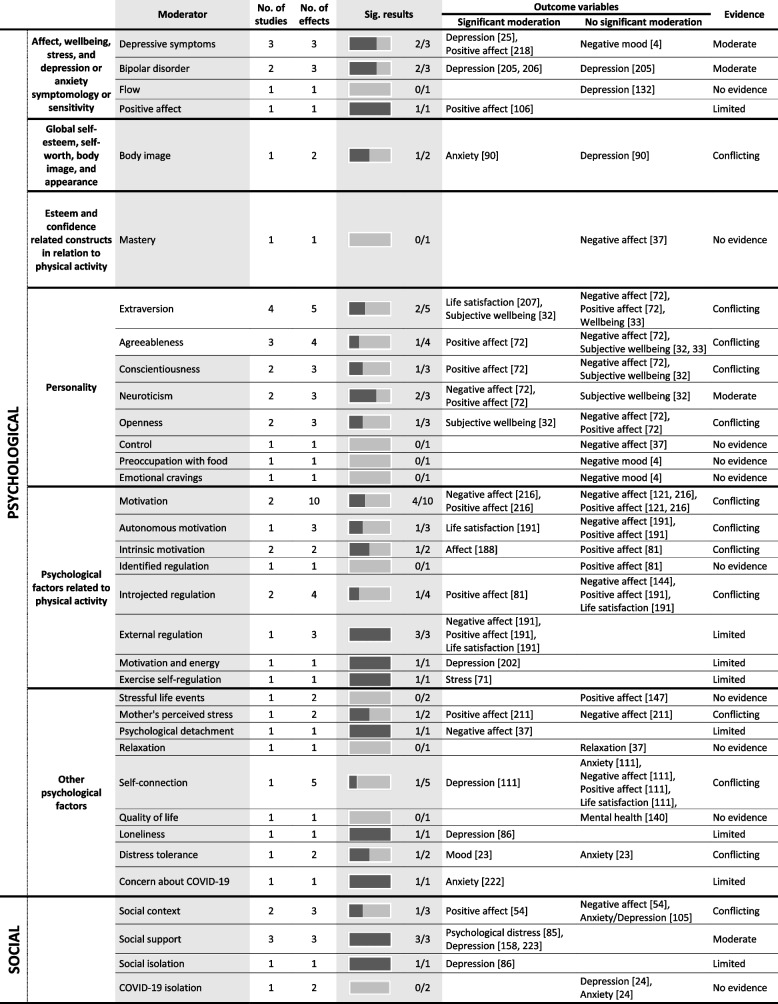

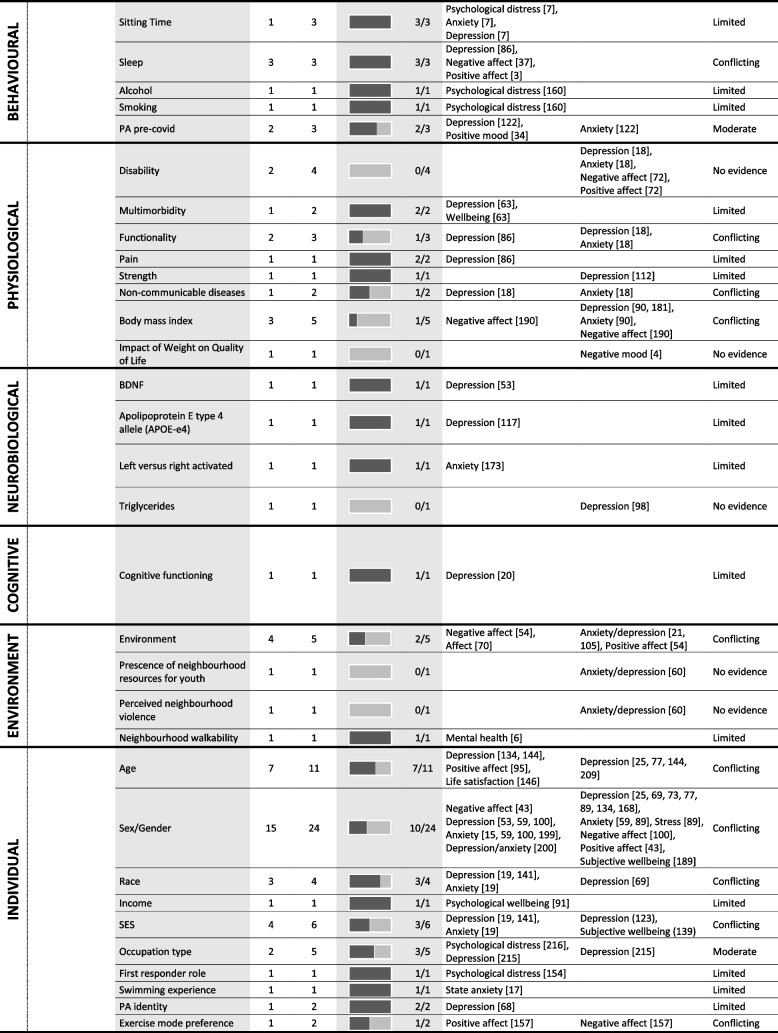
Summary of significant moderators of the association between physical activity and mental health

*PA* physical activity; the numbers in parentheses in the ‘outcome variable’ column refer to the individual study IDs of the 247 included studies

**Table 6:** Summary of evidence for significant mediators and moderators

**Mediators**		**Moderators**
**Strong evidence**	**Moderate evidence**	**Moderate evidence**
• Affect • Mental health • Self-esteem • Self-efficacy • Physical self-worth • Body image satisfaction • Resilience • Social support • Social connection • Physical health • Pain • Fatigue	• Stress • Life satisfaction • Physical/sport competence • Mastery • Hope • Mindfulness • Sense of control • Emotional regulation • Loneliness • Sense of belonging • Socialising • Problematic phone, social media, internet use • Functionality • BMI • Inflammation	• Depressive symptoms • Bipolar disorder • Neuroticism • Social support • Sleep • Race • Age • Occupation *Although the direction is conflicting for sleep, race, age, and occupation.*

## Mediation results

### Psychological factors

#### Affect, wellbeing, stress, and depression or anxiety symptomology or sensitivity

Strong evidence suggests that affect mediates the relationship between PA and mental health. Six studies of acceptable quality reported nine individual effects with 89% supporting mediation. Significant mediation results were reported for depression, anxiety, mental health, and life satisfaction; however, only among adults, including those with depression, anxiety, and multiple sclerosis. Strong evidence suggests that mental health and wellbeing mediates the relationships between PA and other psychological outcomes including depression, anxiety, psychological distress, and life satisfaction. Six studies with five of acceptable or high quality, reported eight individual effects, with 88% supporting mediation. These results were only identified among adults, including those with major depressive disorder. Moderate evidence suggests that stress mediates the relationship between PA and mental health. Four studies (one high quality, two acceptable, and one low) reported six individual effects with 67% supporting mediation. Significant mediation results were reported for depression, psychological distress, and life satisfaction, among adults, and those with a spinal cord injury. Moderate evidence suggests that life satisfaction mediates the relationship between PA and both, wellbeing, and psychological distress. Three studies, two of high quality, reported three individual effects, with 67% supporting mediation. Mediation results were only identified among young adults.

#### Self-esteem, self-worth, self-efficacy, and body image

Strong evidence suggests that self-esteem mediates the relationship between PA and mental health. Six studies (five acceptable quality and one of low quality) reported eight individual results, with 100% supporting mediation. Significant mediation results were reported for depression, anxiety, and wellbeing, among adults including those with a major depressive disorder. Strong evidence suggests that self-efficacy mediates the relationship between PA and mental health. Twelve studies (two of high quality, nine of acceptable quality, and one of low quality) reported 17 individual results, with 94% supporting mediation. Significant mediation was reported across depression, psychological distress, affect, mental health, wellbeing, and life satisfaction among adolescents and adults. Strong evidence suggests that physical self-worth mediates the relationship between PA and mental health. Four studies of high or acceptable quality reported five individual results, with 80% supporting mediation. Significant mediation was reported for depression, anxiety, and life satisfaction among adults. Strong evidence suggests that body image satisfaction mediates the relationship between PA and mental health. Eleven studies (one of high quality, eight of acceptable quality, and one of low quality) reported 18 individual results, with 94% supporting mediation. Significant mediation was reported across depression, anxiety, psychological distress, positive affect mental health, wellbeing, and life satisfaction among adolescents and adults, including cancer survivors.

#### PA self-efficacy, accomplishment, competence, and mastery

Moderate evidence suggests that physical or sport competence mediates the relationship between PA and mental health. Two studies of acceptable quality reported two individual results with 100% supporting mediation. Significant mediation was reported for positive affect and wellbeing only, among only adults. Moderate evidence suggests that mastery mediates the relationship between PA and mental health. Two studies of high or acceptable quality reported two individual results with 100% supporting mediation. Significant mediation was reported for depression and psychological distress only, among only adults but including cancer survivors.

#### Personality

Moderate evidence suggests that hope mediates the relationship between PA and mental health. Two studies, one of low and one of high quality, reported three individual effects, with 100% supporting mediation. Significant mediation results were reported for psychological distress and wellbeing, among adults.

#### Psychological factors related to the PA experience

No evidence suggests scheduling efficacy or behavioural loyalty mediate the relationship between PA and mental health, and conflicting evidence exists for autonomy, competence, and relatedness.

#### Other psychological factors

Moderate evidence suggests that mindfulness mediates the relationship between PA and mental health. Three studies of high or acceptable quality reported five individual results with 60% supporting mediation. Significant mediation was reported for depression and stress only, among only adults, including advanced yoga participants. Moderate evidence suggests that a sense of control mediates the relationship between PA and mental health. Two studies of high or acceptable quality reported five individual results with 100% supporting mediation. Significant mediation was reported for depression, anxiety, stress, mental health and life satisfaction, although only among university students. Moderate evidence suggests that emotional regulation or emotional intelligence mediates the relationship between PA and mental health. Three studies of acceptable quality reported five individual results with 100% supporting mediation. Significant mediation was reported for depression, anxiety, wellbeing, and life satisfaction, among adolescents and young adults. Strong evidence suggests that resilience mediates the relationship between PA and mental health. Ten studies (three of high quality, six of acceptable, and one of low quality) reported 11 individual results with 100% supporting mediation. Significant mediation was reported for depression, anxiety, psychological distress, mental health, and affect, among children, adolescents, and adults, including cancer survivors and children with ADHD. Moderate evidence suggests that loneliness mediates the relationship between PA and mental health. Two studies of high and acceptable quality reported two individual results with 100% supporting mediation. Significant mediation was reported for life satisfaction among adults only.

### Social factors

Strong evidence suggests that social support mediates the relationship between PA and mental health. Seven studies, five of acceptable quality, reported eight individual results with 88% supporting mediation. Significant mediation was reported among adults for depression, anxiety, wellbeing, and life satisfaction. Strong evidence suggests that social connections mediate the relationship between PA and mental health. Four studies (three of high quality and one of acceptable quality) reported four individual results with 75% supporting mediation. Significant mediation was reported only among children and adolescents, for depression, and mental health and wellbeing. Moderate evidence suggests that a sense of belonging mediates the relationship between PA and mental health. Five studies, three of acceptable quality, reported eleven individual results, 91% supporting mediation. Significant mediation was reported for depression, anxiety, psychological distress, positive affect, life satisfaction, and wellbeing, among adults and adolescents. Moderate evidence suggests that socialising/social participation mediates the relationship between PA and mental health. Two studies of high and acceptable quality reported eight individual results, with 100% supporting mediation. Significant mediation was reported among adults for stress and psychological distress.

### Behavioural factors

Moderate evidence suggests that problematic phone, social media, or internet use mediates the relationship between PA and mental health. Three studies of high or acceptable quality reported four individual results with 100% supporting mediation. Significant mediation was reported for depression, stress, psychological distress, and mental health, among adolescents and adults. No evidence supported sitting time or screen time as mediators and evidence was conflicting as to whether sleep was a mediator.

### Physiological mechanisms

Strong evidence suggests that physical health, or medical status, mediates the relationship between PA and mental health. Six studies (one of high quality, three of acceptable quality, and two of low quality) reported seven individual results with 71% supporting mediation. Significant mediation was reported for depression, psychological distress, and wellbeing, although only among university students. Strong evidence suggests that pain mediates the relationship between PA and mental health. Six studies (five of acceptable quality) reported six individual results with 83% supporting mediation. Significant mediation was reported for depression, stress, and psychological distress, among adults, including those with rheumatoid arthritis and spinal cord injury. Strong evidence suggests that fatigue mediates the relationship between PA and mental health. Five studies (two of high quality and three of acceptable quality) reported six individual results with 67% supporting mediation. Significant mediation was reported for depression and life satisfaction, although only among clinical populations diagnosed with Parkinson’s, cancer, or multiple sclerosis. Moderate evidence suggests that functionality mediates the relationship between PA and mental health. Three studies, two of acceptable or higher quality, reported four individual results with 100% supporting mediation. Significant mediation was reported for depression, psychological distress, and life satisfaction, among adults, including breast cancer survivors. Moderate evidence suggests that BMI mediates the relationship between PA and mental health. Three studies of acceptable quality reported four individual results with 75% supporting mediation. Significant mediation was reported for depression, mental health, and wellbeing among children and adults.

### Neurobiological mechanisms

Moderate evidence suggests inflammation mediates the relationship between PA and depression among adults. Two studies of high or acceptable quality reported two individual effects with 100% supporting mediation.

### Cognitive factors

There is conflicting evidence as to whether cognitive function mediates the relationship between PA and mental health.

### Environmental factors

No evidence suggests time spent in nature mediates the relationship between PA and mental health.

## Moderation results

### Psychological factors

#### Affect, wellbeing, stress, and depression or anxiety symptomology or sensitivity

Moderate evidence suggests that depressive symptoms moderate the relationship between PA and mental health. Three studies of acceptable quality reported three individual results, with 67% supporting moderation. For example, Brière, et al. reported that the association between sport participation and subsequent reduced psychological difficulties was stronger in participants who had higher depressive symptoms at baseline [34]. Significant moderation results were only reported for depression and positive affect, among adolescents and adults. Moderate evidence also suggests that bipolar disorder moderates the effect of PA on mental health. Two studies of acceptable quality reported three individual results, with 67% supporting moderation. For example, PA was associated with a reduction in subsequent depressive symptoms for those with bipolar disorder more than those without [35, 36]. These results only examined depression as the outcome variable, among only young adults.

#### Personality

Moderate evidence suggests neuroticism significantly moderates the relationship between PA and mental health. Two studies of high and acceptable quality reported three individual results, with 67% supporting mediation.

#### Psychological factors related to the PA experience

Evidence is conflicting as to whether motivation moderates the relationship between PA and mental health with less than 50% of results supporting moderation.

#### Other psychological factors

No evidence suggests that stressful life events, relaxation, or quality of life moderate the relationship between PA and mental health, with 100% of results indicating no moderation. Evidence is contradictory for mother’s perceived stress, self-connection, or distress tolerance, with less than 50% of results supporting moderation.

### Social factors

Moderate evidence suggests that social support moderates the relationship between PA and mental health. Three studies of acceptable quality reported three individual effects, with 100% supporting moderation. For example, Gyasi showed that the inverse relationship between PA and psychological distress was stronger as levels of perceived social support increased (OR = 0.651; 95% CI = 0.376, 0.727) [37]. Significant moderation results were reported for psychological distress and depression, although only older adults were recruited in these studies. Evidence is conflicting as to whether the social context of PA itself is a moderator.

### Behavioural factors

Moderate evidence suggests sleep moderates the relationship between PA and mental health. Three studies of acceptable or high quality reported three individual effects, with 100% indicating moderation. However the role of sleep as a moderator was unclear, with one study showing PA was only associated with lower negative affect among those engaging in sufficient sleep [38] and another study reporting greater exercise-induced increases in positive affect among those with more sleep disturbances [39].

### Physiological factors

No evidence suggests that disability moderates the relationship between PA and mental health. Evidence is conflicting as to whether functionality or BMI are significant moderators.

### Neurobiological factors

No evidence suggests that triglycerides moderate the relationship between PA and mental health. Evidence for all other neurobiological factors is limited.

### Cognitive factors

Evidence as to whether cognitive functioning moderates the relationship between PA and mental health is limited.

### Environmental factors

No evidence suggests that the presence of violence or neighbourhood resources moderates the relationship between PA and mental health. However, evidence remains conflicting as to whether the environment itself moderates the effect of PA on mental health.

### Individual characteristics

Moderate evidence suggests that race moderates the relationship between PA and mental health. Three studies of acceptable or high quality reported four individual results, with 75% supporting moderation. Significant moderation was reported for depression and anxiety; however, findings are inconclusive as one study reported a stronger association among white participants while another study found stronger associations among those from racial minority groups. Similarly, moderate evidence suggests age moderates the relationship between PA and mental health, however the evidence is conflicting as to whether younger or older populations benefit more. Moderate evidence also suggests that occupation type moderates the effect of PA on mental health. Two studies of high quality reported five individual results, with 60% supporting mediation. However, one study specifically examined work-related PA while the other study assessed multiple domains finding moderation for leisure-time PA, making results difficult to compare. Evidence is conflicting as to whether sex or socioeconomic status moderate the relationship between PA and mental health.

## Discussion

Despite an increasing number of studies demonstrating positive effects of PA on mental health, the underlying mechanisms responsible for mental health benefits have still not been well established. Variables that influence the strength and direction of the relationship have also not been well understood. By systematically combining results from many studies, we now have more conclusive evidence of 27 different mediators, 12 with strong evidence and 15 with moderate evidence. Interestingly, seven of the 12 mediators with strong evidence are psychological mediators, two are social mediators, and three are physical. There is no strong evidence for any behavioural, neurobiological, cognitive, or environmental mediators. Only eight significant moderators were identified, although the direction was not consistent for four of them. Among the four consistent moderators, three were psychological moderators and one was social.

Self-efficacy, sleep, body image satisfaction, and resilience were the most examined mediators, with 10 or more studies examining each mediator. Self-efficacy theory [280] has been long-proposed as a possible mechanism for the effect of PA on mental health [15]. Given that PA poses a challenging task, successfully engaging in exercise may increase self-efficacy (i.e., confidence in one’s ability to exercise), and this increased confidence in one’s ability to exercise may produce improved mood [281, 282]. However, the mediation results reported in this review indicated that global self-esteem (i.e., the way people feel about themselves overall), global self-efficacy, and physical self-worth were significant mediators, whereas evidence was conflicting for exercise self-efficacy. Moderate evidence did however suggest that both mastery and competence are more tenable mechanisms than exercise self-efficacy. For people to experience a sense of mastery or competence, activities and exercises need to be optimally challenging, group targets or goals should be avoided, and a task environment should be promoted [283, 284]. It’s possible that a sense of mastery and accomplishment translates into improvements in global self-efficacy and self-esteem, more than exercise self-efficacy translates into global self-efficacy. However, the process by which mastery leads to global self-esteem or self-efficacy needs to be further understood.

Physical self-worth and body image satisfaction were also both strong mediators and may also both contribute to global self-esteem. However, very few studies have examined body related psychological variables alongside physical competence or mastery related variables, to understand how each contributes to global self-esteem or global constructs of mental health. It may be likely that the same strategies, behaviours, and PA environments similarly target all these mediators, however, the level of evidence to date is dictated by the number of studies and the quality of those studies, rather than which mediator truly plays a stronger role, as evidence is consistent for all of these mediators. While resilience has been well studied as an outcome of sport participation [285–287], it has been less-studies in relation to PA more broadly, meaning it’s not entirely clear how resilience can be enhanced or developed through different exercise contexts. Therefore, further research should consider examining how different instructional styles and trainer behaviours, as well as individual actions of participants themselves, lead to the development of resilience.

Evidence is also rather consistent for both affect and mental health. For example, strong evidence showed that affect was a significant mediator of the associations between PA and, depression, anxiety, mental health, and life satisfaction. This suggests that temporary state-based moods, emotions, and affective experiences are an important element of the PA experience, and while they may be temporary, consistent experiences of positive affect contribute to overall positive mental health and wellbeing, and may protect against the onset of a mental health disorder, or reduce symptoms of depression and anxiety [288, 289]. There was also moderate evidence of existing mental health conditions moderating the effect of PA on mental health, whereby stronger effects were observed among those with existing symptoms or pre-existing diagnoses. Therefore, PA represents a suitable mental health promotion strategy for the whole population, with possibly the greatest benefit obtained by those most in need of mental health improvements.

As early as the 1980’s it was recognised that the group interaction and social attention one receives when exercising may be responsible for increased mood post-exercise [290, 291]. However, early research showed that exercise alone also facilitated mental health improvements [282]. Debate, and inconsistent findings, over the role of social interaction, continue today, with evidence still supporting both individual and group-based PA [16]. The results of this review mirror this debate, as there was conflicting evidence as to whether the social context moderates the effect of PA on mental health. There was, however, strong evidence that social *support* moderated the effect, such that the association between PA and mental health was greater among those reporting greater social support. Strong evidence also shows that both social support and social connection mediate the relationship between PA and mental health, and moderate evidence supports a sense of belonging and socialising as mediators. These findings collectively suggest that feelings of support and belonging may be more important to the mental health benefits of PA, than the mere opportunity for social interaction. Therefore, practitioners need to guide individuals toward selecting activities or exercise environments that are supportive, rather than encouraging group activities.

One of the earliest proposed psychological mechanisms was the cognitive-behavioural hypothesis. This hypothesis proposed that positive thoughts during PA break or interrupt the downward thought-affect spiral whereby negative thoughts lead to negative affective states, leading to depressive symptoms [282, 292]. However, no studies in this review measured distraction as a mediator. Moderate evidence did however suggest that mindfulness was a significant mediator. Results also suggested that reduced rumination and psychological detachment could be important mediators, although evidence is limited. Many aspects of the PA environment can enhance the distractive power of PA, or increase the possible mindfulness that come from reducing rumination and detaching from either work or stress, including nature, pleasant scenery, group interaction, and activities with high attention requirements [17]. These factors can be used to recommend engagement in activities that will likely provide a distraction. However, additional studies are needed to better understand the roles of rumination, mindfulness, distraction, and detachment, as evidence is limited although promising.

Interestingly, evidence regarding the role of motivation was contradictory, as was any evidence of the satisfaction of psychological needs (i.e., competence, autonomy, and relatedness). It is important to note though, that all studies that assessed autonomy, competence, or relatedness as potential mediators, measured these constructs in relation to PA. While it is possible that autonomy, competence, and relatedness specifically in relation to one type of exercise might not enhance overall mental health, research shows that PA can be used as an opportunity to participate in an activity that satisfies people’s overall psychological needs [17]. For example, people who are more active, may experience feelings of autonomy, competence, and relatedness more regularly on a weekly basis, and consequently experience greater mental health. However, for this to be determined, global need satisfaction would have to be examined as a possible mediator, rather than exercise-specific need satisfaction.

Given that PA elicits a wide variety of biochemical changes in the brain [8], many physiological, neurobiological, and biochemical mechanisms have been long proposed as responsible for the effect of PA on mental health [15]. These mechanisms include the release of endorphins [293], the transmission of monoamine neurotransmitters, including serotonin, dopamine, and norepinephrine [282], and more recently neuroplasticity, inflammation, and oxidative stress [8]. These mechanisms have all been criticised for oversimplifying the effect of PA, and evidence appears limited [15, 293]. This review supports the idea that neurobiological mechanisms may not be primary pathways by which PA benefits mental health as inflammation was the only neurobiological mediator with moderate evidence, despite 11 neurobiological mediators being examined. However, evidence is limited for many of them. Physical health, pain, fatigue, and functionality, however, did all mediate the relationship between PA and mental health, suggesting that PA may simultaneously improve aspects of physical and mental health; however, experimental or longitudinal studies are needed to determine whether PA leads to improved physical health, which leads to improved mental health. Overall, there is very little evidence for neurobiological or physiological mechanisms, relative to psychological and social mechanisms. This is advantageous to the future of mental health promotion through PA, as activities, exercises, instructional approaches, and environments can all be tailored to specifically target psychological and social mediators and moderators with strong evidence.

### Strengths and limitations

This is the first systematic review to combine evidence of moderators and mediators. It presents the most comprehensive evidence to date, explaining the mechanisms that enable PA to benefit mental health, and the factors that influence the strength of this relationship. The broad variety of mental health outcomes included is also a strength, as many studies examine mediators or moderators in relation to one particular outcome (e.g., depression). Nevertheless, several notable limitations exist. Firstly, a meta-analysis was not possible with the data obtained given the breadth of different mediators, moderators, and outcomes included. Perhaps, as the number of studies in this area expands, meta-analyses could be conducted across several mediators for each outcome. Secondly, most included studies were cross-sectional. While a review can only include studies existing in the literature, perhaps future experimental studies could consider testing the range of mediators examined in this review, and publish mediation findings whether significant or not, to help understand mechanisms from PA to mental health. Thirdly, several of the mediators and moderators reported in this review were only examined in one empirical study. This means evidence is limited for these constructs and it’s unknown whether they could be significant mediators or moderators. All variables with limited evidence need further examination in future studies.

## Conclusions

Despite PA becoming increasingly accepted and adopted as a mental health strategy in recent years, most recommendations only focus on the amount of PA needed for health benefits. This comprehensive review provides evidence of all mediators and moderators. Taking these findings into consideration when designing PA experiences, running exercise classes, or prescribing or recommending PA to clients, patients, or participants, will enable PA to have greater impacts on population mental health. 

## Supplementary Information

Additional file 1. Search Terms. Search terms. Includes the full search ran in Scopus to identify studies.

Additional file 2. Studies included in systematic review. List of all studies included in the review with their corresponding study number. A table presenting characteristics of each included study.

Additional file 3. Study characteristics Table 14AUG24. Study Characteristics Table. A table presenting characteristics of each included study.

Additional file 4. Results tables 16AUG24. Results tables. A file including the individual results extracted for each included study.

Additional file 5. Risk of Bias Table 14AUG24. Risk of Bias table. Results of the risk of bias assessment for each included study.

## Data Availability

All data generated or analysed during this study are included in this published article (specifically, within Additional File 3).

## Abbreviation

PA: Physical activity
