# Supplementary material for: Physical activity and mental health: a systematic review and best-evidence synthesis of mediation and moderation studies
Source: Int J Behav Nutr Phys Act. 2024 Nov 28;21:134. doi: 10.1186/s12966-024-01676-6 (PMC11603721; doi:10.1186/s12966-024-01676-6)
Supplement: Supplementary file 1 — Additional file 1. Search Terms. Search terms. Includes the full search ran in Scopus to identify studies. [file 12966_2024_1676_MOESM1_ESM.pdf]

Supplementary File 1: Search Terms

Mental health cluster	Physical activity cluster	Mediation/moderation cluster
"mental health"	"physical activit*"	moderation
"mental well*being"	"physical inactivity"	mediation
"psychological well*being"	exercise	moderator
"subjective well*being"	sport*	mediator
"life satisfaction"	walking	moderated
"positive affect"		mediated
"negative affect"		moderating
"mental ill*"		mediating
"mental disorder*"		
depress*		
anxiety		
stress		
"psychological distress"		

Exported search from Scopus

TITLE-ABS-KEY ("mental health") OR TITLE-ABS-KEY ("mental well*being") OR TITLE-ABS-KEY ("psychological well*being") OR TITLE-ABS-KEY ("subjective well*being") OR TITLE-ABS-KEY ("life satisfaction") OR TITLE-ABS-KEY ("positive affect") OR TITLE-ABS-KEY ("negative affect") OR TITLE-ABS-KEY ("mental ill*") OR TITLE-ABS-KEY ("mental disorder*") OR TITLE-ABS-KEY (depress*) OR TITLE-ABS-KEY (anxiety) OR TITLE-ABS-KEY (stress) OR TITLE-ABS-KEY ("psychological distress") AND TITLE-ABS-KEY ("physical activit*") OR TITLE-ABS-KEY ("physical inactivity") OR TITLE-ABS-KEY (exercise) OR TITLE-ABS-KEY (sport*) OR TITLE-ABS-KEY (walking) AND TITLE-ABS-KEY (moderation) OR TITLE-ABS-KEY (mediation) OR TITLE-ABS-KEY (moderator) OR TITLE-ABS-KEY (mediator) OR TITLE-ABS-KEY (moderated) OR TITLE-ABS-KEY (mediated) OR TITLE-ABS-KEY (moderating) OR TITLE-ABS-KEY (mediating)
