# Supplementary material for: Physical activity and mental health: a systematic review and best-evidence synthesis of mediation and moderation studies
Source: Int J Behav Nutr Phys Act. 2024 Nov 28;21:134. doi: 10.1186/s12966-024-01676-6 (PMC11603721; doi:10.1186/s12966-024-01676-6)
Supplement: Supplementary file 2 — Additional file 2. Studies included in systematic review. List of all studies included in the review with their corresponding study number. A table presenting characteristics of each included study. [file 12966_2024_1676_MOESM2_ESM.pdf]

Studies included in systematic review

The number next to each reference is the study ID allocated to each of the 247 included studies. These numbers can be used to locate the original research referred to in Tables 4 and 5 in the manuscript as well as Tables S1-S4 in the Additional Files.

1. Adams SC, Delorey DS, Davenport MH, Fairey AS, North S, Courneya KS. Effects of high-intensity interval training on fatigue and quality of life in testicular cancer survivors. Br J Cancer. 2018;118(10):1313-21.
2. Aguiñaga S, Ehlers DK, Salerno EA, Fanning J, Motl RW, McAuley E. Home-based physical activity program improves depression and anxiety in older adults. J Phys Act Health. 2018;15(9):692-6.
3. Alfini AJ, Won J, Weiss LR, Nyhuis CC, Shackman AJ, Spira AP, et al. Impact of exercise on older adults' mood is moderated by sleep and mediated by altered brain connectivity. Soc Cogn Affect Neurosci. 2020;15(11):1238-51.
4. Annesi J. Moderating effects of depression, food cravings, and weight-related quality-of-life on associations of treatment-targeted psychosocial changes and physical activity in adolescent candidates for bariatric surgery. J Phys Act Health. 2018;15(12):946-53.
5. Annesi JJ. Gender effects on theory-based psychosocial predictors of increased physical activity, and its subsequent influences on relations of fatigue with other psychosocial factors within Parkinson disease physical activity classes. Minerva Psichiatr. 2019;60(1):1-11.
6. Asiamah N, Vieira ER, Kouveliotis K, Gasana J, Awuviry-Newton K, Eduafo R. Associations between older African academics' physical activity, walkability and mental health: a social distancing perspective. Health Promot Int. 2022;37(2).
7. Asztalos M, Cardon G, De Bourdeaudhuij I, De Cocker K. Cross-sectional associations between sitting time and several aspects of mental health in Belgian adults. J Phys Act Health. 2015;12(8):1112-8.
8. Awick EA, Ehlers DK, Aguiñaga S, Daugherty AM, Kramer AF, McAuley E. Effects of a randomized exercise trial on physical activity, psychological distress and quality of life in older adults. Gen Hosp Psychiatry. 2017;49:44-50.
9. Backström-Eriksson L, Bergsten-Brucefors A, Hjelte L, Melin B, Sorjonen K. Associations between genetics, medical status, physical exercise and psychological well-being in adults with cystic fibrosis. BMJ Open Respiratory Research. 2016;3(1).
10. Bae MH, Zhang X, Lee JS. Exercise, grit, and life satisfaction among Korean adolescents: a latent growth modeling analysis. BMC Public Health. 2024;24(1).

11. Baker T, White R, Abbott G, Litterbach E, Teychenne M. Investigating psychosocial and behavioural mediators of the relationship between physical activity and depressive symptoms in women from socioeconomically disadvantaged neighbourhoods. Ment Health Phys Act. 2023;25.
12. Bang H, Chang M, Kim S. Team and individual sport participation, school belonging, and gender differences in adolescent depression. Children and Youth Services Review. 2024;159.
13. Barham WT, Buysse DJ, Kline CE, Kubala AG, Brindle RC. Sleep health mediates the relationship between physical activity and depression symptoms. Sleep and Breathing. 2022;26(3):1341-9.
14. Barr NG, Martin Ginis KA, Arent SM. The acute effects of arm ergometry on affect. Comparative Exercise Physiology. 2010;7(3):117-25.
15. Bartholomew JB, Linder DE. State anxiety following resistance exercise: The role of gender and exercise intensity. J Behav Med. 1998;21(2):205-19.
16. Baruth M, Wilcox S, Schoffman DE, Becofsky K. Understanding the effects of a self-directed exercise program on depressive symptoms among adults with arthritis through serial mediation analyses. Ment Health Phys Act. 2016;11:13-8.
17. Berger BG, Owen DR. Anxiety reduction with swimming: relationships between exercise and state, trait, and somatic anxiety. / Diminution de l' anxiete en natation: relations entre l' anxiete d' exercice et d' etat de caractere et somatique. Int J Sport Psychol. 1987;18(4):286-302.
18. Bhandari P, Paswan B. Lifestyle Behaviours and Mental Health Outcomes of Elderly: Modification of Socio-Economic and Physical Health Effects. Ageing International. 2020.
19. Biese KM, McGuine TA, Haraldsdottir K, Reardon C, Watson AM. The Influence of Race, Socioeconomic Status, and Physical Activity on the Mental Health Benefits of Sport Participation During COVID-19. Sports Health. 2024;16(2):195-203.
20. Birch K, ten Hope M, Malek-Ahmadi M, O'Connor K, Schofield S, Coon D, et al. Cognitive function as a mediator in the relationship between physical activity and depression status in older adults. Journal of Aging and Physical Activity. 2016;24(4):540-6.
21. Bodin M, Hartig T. Does the outdoor environment matter for psychological restoration gained through running? Psychol Sport Exerc. 2003;4(2):141-53.
22. Booij SH, Bos EH, Jonge P, Oldehinkel AJ. Markers of stress and inflammation as potential mediators of the relationship between exercise and depressive symptoms: Findings from the trails study. Psychophysiology. 2015;52(3):352-8.
23. Borges AM, Uebelacker LA, Brown RA, Price LH, Abrantes AM. An examination of the effects of distress intolerance and rating of perceived exertion on changes in mood and

anxiety following aerobic exercise among treatment-seeking smokers. Psychology, Health and Medicine. 2022.

24. Brady SM, Fenton SAM, Metsios GS, Bosworth A, Duda JL, Kitas GD, et al. Different types of physical activity are positively associated with indicators of mental health and psychological wellbeing in rheumatoid arthritis during COVID-19. Rheumatol Int. 2021;41(2):335-44.
25. Brière FN, Yale-Soulière G, Gonzalez-Sicilia D, Harbec M-J, Morizot J, Janosz M, et al. Prospective associations between sport participation and psychological adjustment in adolescents. J Epidemiol Community Health. 2018;72(7):575-81.
26. Broman-Fulks JJ, Abraham CM, Thomas K, Canu WH, Nieman DC. Anxiety sensitivity mediates the relationship between exercise frequency and anxiety and depression symptomology. Stress and Health: Journal of the International Society for the Investigation of Stress. 2018.
27. Buffart LM, Ros WJG, Chinapaw MJM, Brug J, Knol DL, Korstjens I, et al. Mediators of physical exercise for improvement in cancer survivors' quality of life. Psychooncology. 2014;23(3):330-8.
28. Castan A, Bonilla I, Chamarro A, Saurí J. Psychosocial Outcomes Associated With Types and Intensities of Physical Activity in People With Spinal Cord Injury: The Mediating Role of Self-Efficacy and Functionality. Journal of Physical Activity and Health. 2024;21(5):481-90.
29. Cecchini JA, Fernandez-Río J, Mendez-Gimenez A. Physical activity, approach-avoidance temperament and depressive symptoms. Kinesiology. 2019;51(1):60-9.
30. Chae SM, Kang HS, Ra JS. Body esteem is a mediator of the association between physical activity and depression in Korean adolescents. Appl Nurs Res. 2017;33:42-8.
31. Chair SY, Cheng HY, Chew HSJ, Zang YL, Siow EKC, Cao X. Leisure-Time Physical Activity and Depressive Symptoms Among Patients With Coronary Heart Disease: The Mediating Role of Physical Activity Self-Efficacy. Worldviews Evid Based Nurs. 2020.
32. Chan BCL, Luciano M, Lee B. Interaction of physical activity and personality in the subjective wellbeing of older adults in Hong Kong and the United Kingdom. Behavioral Sciences. 2018;8(8).
33. Chan BCL, Luciano M, Lee B. A Longitudinal Study of Physical Activity and Personality in the Wellbeing of Older Adults. J Aging Health. 2023.
34. Chang YK, Hung CL, Timme S, Nosrat S, Chu CH. Exercise behavior and mood during the COVID-19 pandemic in Taiwan: Lessons for the future. Int J Environ Res Public Health. 2020;17(19):1-17.

35. Chen YC, Putnam M, Lee YS, Morrow-Howell N. Activity Patterns and Health Outcomes in Later Life: The Role of Nature of Engagement. The Gerontologist. 2019;59(4):698-708.
36. Chen R, Liu YF, Huang GD, Wu PC. The relationship between physical exercise and subjective well-being in Chinese older people: The mediating role of the sense of meaning in life and self-esteem. Front Psychol. 2022;13:1029587.
37. Cho S, Park Y. How to benefit from weekend physical activities: Moderating roles of psychological recovery experiences and sleep. Stress and Health. 2018;34(5):639-48.
38. Choi NG, Choi BY, Marti CN. Mediation of the Association Between Physical Exercise and Depressive/Anxiety Symptoms by Pain and Sleep Problems Among Older Adults. Gerontology and Geriatric Medicine. 2024;10.
39. Chu Q, Wong CCY, He G, Yang J, Chen C, He Y. Walking activity and emotional distress among breast cancer survivors: the parallel mediating effects of posttraumatic growth and body image. Support Care Cancer. 2023;31(3).
40. Clément JF, Gallant F, Hudon C, Montiel C, Riglea T, Berbiche D, et al. Use of physical activity as a coping strategy mediates the association between adolescent team sports participation and emerging adult mental health. Ment Health Phys Act. 2024;27.
41. Condello G, Capranica L, Stager J, Forte R, Falbo S, Di Baldassarre A, et al. Physical activity and health perception in aging: Do body mass and satisfaction matter? A three-path mediated link. PLoS One. 2016;11(9).
42. Conley MI, Hindley I, Baskin-Sommers A, Gee DG, Casey BJ, Rosenberg MD. The importance of social factors in the association between physical activity and depression in children. Child and Adolescent Psychiatry and Mental Health. 2020;14(1).
43. Costigan SA, Lubans DR, Lonsdale C, Sanders T, del Pozo Cruz B. Associations between physical activity intensity and well-being in adolescents. Preventive Medicine: An International Journal Devoted to Practice and Theory. 2019;125:55-61.
44. Dahlstrand J, Friberg P, Fridolfsson J, Börjesson M, Arvidsson D, Ekblom Ö, et al. The use of coping strategies “shift-persist” mediates associations between physical activity and mental health problems in adolescents: a cross-sectional study. BMC Public Health. 2021;21(1).
45. Dang K, Ritvo P, Katz J, Gratzer D, Knyahnytska Y, Ortiz A, et al. The Role of Daily Steps in the Treatment of Major Depressive Disorder: Secondary Analysis of a Randomized Controlled Trial of a 6-Month Internet-Based, Mindfulness-Based Cognitive Behavioral Therapy Intervention for Youth. Interact J Med Res. 2023;12:e46419.
46. Deng Y, Lee K, Lam MHS, Lee PH. Understanding sociobehavioral mitigators of depressive symptoms among US young adults. Behav Med. 2016;42(4):217-26.

47. Deng Y, Paul DR. The relationships between depressive symptoms, functional health status, physical activity, and the availability of recreational facilities: A rural-urban comparison in middle-aged and older Chinese adults. Int J Behav Med. 2018;25(3):322-30.
48. Deng J, Liu Y, Chen R, Wang Y. The Relationship between Physical Activity and Life Satisfaction among University Students in China: The Mediating Role of Self-Efficacy and Resilience. Behavioral Sciences. 2023;13(11).
49. Dong Z, Wang P, Xin X, Li S, Wang J, Zhao J, et al. The relationship between physical activity and trait anxiety in college students: The mediating role of executive function. Front Hum Neurosci. 2022;16.
50. Dong RB, Dou KY, Luo J. Construction of a model for adolescent physical and mental health promotion based on the multiple mediating effects of general self-efficacy and sleep duration. BMC Public Health. 2023;23(1).
51. Donyaei A, Kiani E, Bahrololoum H, Moser O. Effect of combined aerobic–resistance training and subsequent detraining on brain-derived neurotrophic factor (BDNF) and depression in women with type 2 diabetes mellitus: A randomized controlled trial. Diabet Med. 2024;41(3).
52. Doré I, Sylvester B, Sabiston C, Sylvestre MP, O'Loughlin J, Brunet J, et al. Mechanisms underpinning the association between physical activity and mental health in adolescence: A 6-year study. Int J Behav Nutr Phys Act. 2020;17(1).
53. Dotson VM, Hsu FC, Langaee TY, McDonough CW, King AC, Cohen RA, et al. Genetic Moderators of the Impact of Physical Activity on Depressive Symptoms. The Journal of frailty & aging. 2016;5(1):6-14.
54. Dunton GF, Liao Y, Intille S, Huh J, Leventhal A. Momentary assessment of contextual influences on affective response during physical activity. Health Psychol. 2015;34(12):1145-53.
55. Eddolls WTB, McNarry MA, Lester L, Winn CON, Stratton G, Mackintosh KA. The association between physical activity, fitness and body mass index on mental well-being and quality of life in adolescents. Quality of Life Research: An International Journal of Quality of Life Aspects of Treatment, Care & Rehabilitation. 2018;27(9):2313-20.
56. Elavsky S, McAuley E. Physical activity, symptoms, esteem, and life satisfaction during menopause. Maturitas. 2005;52(3-4):374-85.
57. Elavsky S, McAuley E, Motl RW, Konopack JF, Marquez DX, Hu L, et al. Physical activity enhances long-term quality of life in older adults: Efficacy, esteem, and affective influences. Ann Behav Med. 2005;30(2):138-45.

58. Elavsky S, Gold CH. Depressed mood but not fatigue mediate the relationship between physical activity and perceived stress in middle-aged women. Maturitas. 2009;64(4):235-40.
59. Evans M, Rohan KJ, Howard A, Ho S-Y, Dubbert PM, Stetson BA. Exercise dimensions and psychological well-being: A community-based exercise study. J Clin Sport Psychol. 2017;11(2):107-25.
60. Fauth RC, Roth JL, Brooks-Gunn J. Does the neighborhood context alter the link between youth's after-school time activities and developmental outcomes? A multilevel analysis. Dev Psychol. 2007;43(3):760-77.
61. Feng W, Zhao L, Ge Z, Zhao X, Li T, Zhu Q. Association between physical activity and adolescent mental health in the post COVID-19: The chain mediating effect of self-esteem and social anxiety. PLoS One. 2024;19(5 May).
62. Fernandes HM, Costa H, Esteves P, Machado-Rodrigues AM, Fonseca T. Direct and Indirect Effects of Youth Sports Participation on Emotional Intelligence, Self-Esteem, and Life Satisfaction. Sports. 2024;12(6).
63. Fessler L, Maltagliati S, Sieber S, Cullati S, Tessitore E, Craviari C, et al. Physical activity matters for everyone's health, but individuals with multimorbidity benefit more. Preventive Medicine Reports. 2023;34.
64. Feuerhahn N, Sonnentag S, Woll A. Exercise after work, psychological mediators, and affect: A day-level study. European Journal of Work and Organizational Psychology. 2014;23(1):62-79.
65. Fisher HM, Jacobs JM, Taub CJ, Lechner SC, Lewis JE, Carver CS, et al. How changes in physical activity relate to fatigue interference, mood, and quality of life during treatment for non-metastatic breast cancer. Gen Hosp Psychiatry. 2018;49:37-43.
66. Fontana F, Bourbeau K, Moriarty T, da Silva MP. The Relationship between Physical Activity, Sleep Quality, and Stress: A Study of Teachers during the COVID-19 Pandemic. Int J Environ Res Public Health. 2022;19(23).
67. Foroughi A, Henschel NT, Shahi H, Hall SS, Meyers LS, Sadeghi K, et al. Keeping Things Positive: Affect as a Mediator between Physical Activity and Psychological Functioning. European Journal of Investigation in Health, Psychology and Education. 2023;13(11):2428-59.
68. Forshaw A, Lee Alfrey K, Maher JP, Rebar AL. But that's who I Am: The inability to enact physical activity identity is associated with depression and anxiety symptoms. Ment Health Phys Act. 2023;24.
69. Fredricks JA, Eccles JS. Is extracurricular participation associated with beneficial outcomes? Concurrent and longitudinal relations. Dev Psychol. 2006;42(4):698-713.

70. Geniole SN, David JPF, Euzébio RFR, Toledo BZS, Neves AIM, McCormick CM. Restoring land and mind: The benefits of an outdoor walk on mood are enhanced in a naturalized landfill area relative to its neighboring urban area. Ecopsychology. 2016;8(2):107-20.
71. Gerber M, Lindwall M, Brand S, Lang C, Elliot C, Pühse U. Longitudinal relationships between perceived stress, exercise self-regulation and exercise involvement among physically active adolescents. J Sports Sci. 2015;33(4):369-80.
72. Giacobbi Jr PR, Hardin B, Frye N, Hausenblas HA, Sears S, Stegelin A. A Multi-Level Eamination of Personality, Eercise, and Daily Life Events for Individuals With Physical Disabilities. Adapted Physical Activity Quarterly. 2006;23(2):129-47.
73. Giannotta F, Nilsson KW, Åslund C, Larm P. Frequency of vigorous physical activity and depressive symptoms across adolescence: Disentangling the reciprocal associations between different groups and subtypes of symptoms. Ment Health Phys Act. 2023;25.
74. Ginis KAM, Latimer AE, McKechnie K, Ditor DS, McCartney N, Hicks AL, et al. Using exercise to enhance subjective well-being among people with spinal cord injury: The mediating influences of stress and pain. Rehabil Psychol. 2003;48(3):157-64.
75. Ginoux C, Isoard-Gautheur S, Teran-Escobar C, Forestier C, Chalabaev A, Clavel A, et al. Being active during the lockdown: The recovery potential of physical activity for well-being. Int J Environ Res Public Health. 2021;18(4):1-14.
76. Goldstein E, Topitzes J, Brown RL, Barrett B. Mediational pathways of meditation and exercise on mental health and perceived stress: A randomized controlled trial. J Health Psychol. 2020;25(12):1816-30.
77. Gomez-Baya D, Mendoza R, de Matos MG, Tomico A. Sport participation, body satisfaction and depressive symptoms in adolescence: A moderated-mediation analysis of gender differences. Eur J Dev Psychol. 2019;16(2):183-97.
78. González-Hernández J, Gómez-López M, Pérez-Turpin JA, Muñoz-Villena AJ, Andreu-Cabrera E. Perfectly active teenagers. When does physical exercise help psychological well-being in adolescents? Int J Environ Res Public Health. 2019;16(22).
79. Görgülü E, Bieber M, Engeroff T, Zabel K, Etyemez S, Prvulovic D, et al. Physical activity, physical self-perception and depression symptoms in patients with major depressive disorder: a mediation analysis. Eur Arch Psychiatry Clin Neurosci. 2021;271(7):1205-15.
80. Greenleaf C, Boyer EM, Petrie TA. High school sport participation and subsequent psychological well-being and physical activity: The mediating influences of body image, physical competence, and instrumentality. Sex Roles: A Journal of Research. 2009;61(9-10):714-26.

81. Guérin E, Fortier MS. The moderating influence of situational motivation on the relationship between preferred exercise and positive affect: An experimental study with active women. SAGE Open. 2013;3(4).
82. Guicciardi M, Carta M, Pau M, Cocco E. The relationships between physical activity, self-efficacy, and quality of life in people with multiple sclerosis. Behavioral Sciences. 2019;9(12).
83. Gujral S, Burns M, Erickson KI, Rofey D, Peiffer JJ, Laws SM, et al. Dose-response effects of exercise on mental health in community-dwelling older adults: Exploration of genetic moderators. Int J Clin Health Psychol. 2024;24(1).
84. Guo S, Fu H, Guo K. Effects of physical activity on subjective well-being: the mediating role of social support and self-efficacy. Frontiers in Sports and Active Living. 2024;6.
85. Gyasi RM. Social support, physical activity and psychological distress among community-dwelling older Ghanaians. Arch Gerontol Geriatr. 2019;81:142-8.
86. Gyasi RM, Quansah N, Boateng PA, Akomeah E, Yakubu AF, Ahiabli PA, et al., editors. Meeting the WHO Physical Activity Guidelines is Associated With Lower Odds of Depression in Older Adults: Potential Psychosomatic Mechanisms. 2024.
87. Hachenberger J, Li YM, Lemola S. Physical activity, sleep and affective wellbeing on the following day: An experience sampling study. J Sleep Res. 2023;32(2):e13723.
88. Hachenberger J, Teuber Z, Li YM, Abkai L, Wild E, Lemola S. Investigating associations between physical activity, stress experience, and affective wellbeing during an examination period using experience sampling and accelerometry. Sci Rep. 2023;13(1).
89. Halliday AJ, Kern ML, Turnbull DA. Can physical activity help explain the gender gap in adolescent mental health? A cross-sectional exploration. Ment Health Phys Act. 2019;16:8-18.
90. Han B, Du G, Yang Y, Chen J, Sun G. Relationships between physical activity, body image, BMI, depression and anxiety in Chinese college students during the COVID-19 pandemic. BMC Public Health. 2023;23(1).
91. Hayes D, Ross CE. Body and mind: The effect of exercise, overweight, and physical health on psychological well-being. J Health Soc Behav. 1986;27(4):387-400.
92. Herring MP, Gordon BR, McDowell CP, Quinn LM, Lyons M. Physical activity and analogue anxiety disorder symptoms and status: Mediating influence of social physique anxiety. J Affect Disord. 2021;282:511-6.
93. Herzog E, Voß M, Keller V, Koch S, Takano K, Cludius B. The benefits of physical exercise on state anxiety: Exploring possible mechanisms. Ment Health Phys Act. 2022;23.

94. Ho FKW, Louie LHT, Chow CB, Wong WHS, Ip P. Physical activity improves mental health through resilience in Hong Kong Chinese adolescents. BMC Pediatr. 2015;15(1).
95. Hogan CL, Mata J, Carstensen LL. Exercise holds immediate benefits for affect and cognition in younger and older adults. Psychol Aging. 2013;28(2):587-94.
96. Hogan CL, Catalino LI, Mata J, Fredrickson BL. Beyond emotional benefits: Physical activity and sedentary behaviour affect psychosocial resources through emotions. Psychol Health. 2015;30(3):354-69.
97. Hou B, Wu Y, Huang Y. Physical exercise and mental health among older adults: the mediating role of social competence. Frontiers in Public Health. 2024;12.
98. Huang Y, Xu P, Fu X, Ren Z, Cheng J, Lin Z, et al. The effect of triglycerides in the associations between physical activity, sedentary behavior and depression: An interaction and mediation analysis. J Affect Disord. 2021;295:1377-85.
99. Huang JH, Li RH, Tsai LC. Relationship between Depression with Physical Activity and Obesity in Older Diabetes Patients: Inflammation as a Mediator. Nutrients. 2022;14(19).
100. Hunt-Shanks T, Blanchard C, Reid RD. Gender differences in cardiac patients: A longitudinal investigation of exercise, autonomic anxiety, negative affect and depression. Psychology, Health and Medicine. 2009;14(3):375-85.
101. Jenkins M, Houge Mackenzie S, Hodge K, Hargreaves EA, Calverley JR, Lee C. Physical Activity and Psychological Well-Being During the COVID-19 Lockdown: Relationships With Motivational Quality and Nature Contexts. Front Sports Act Living. 2021;3:637576.
102. Jenkins M, Lee C, Houge Mackenzie S, Hargreaves EA, Hodge K, Calverley J. Nature-Based Physical Activity and Hedonic and Eudaimonic Wellbeing: The Mediating Roles of Motivational Quality and Nature Relatedness. Front Psychol. 2022;13.
103. Ji L, Ren Z, Chen J, Zhao H, Zhang X, Xue B, et al. Associations of vegetable and fruit intake, physical activity, and school bullying with depressive symptoms in secondary school students: the mediating role of internet addiction. BMC Psychiatry. 2024;24(1).
104. Jia Y, Gao X. A chained mediator model: the effect of physical activity on negative emotions in obese adolescents. Gazzetta Medica Italiana Archivio per le Scienze Mediche. 2024;183(3):146-53.
105. Johansson M, Hartig T, Staats H. Psychological benefits of walking: Moderation by company and outdoor environment. Applied Psychology: Health and Well-Being. 2011;3(3):261-80.
106. Johnson MN, Maher JP, Meadows CC, Bittel KM, Hevel DJ, Drollette ES. Positive affect moderates inhibitory control and positive affect following a single bout of self-select aerobic exercise. Psychol Sport Exerc. 2022;60.

107. Joseph RP, Royse KE, Benitez TJ, Pekmezi DW. Physical activity and quality of life among university students: Exploring self-efficacy, self-esteem, and affect as potential mediators. Quality of Life Research: An International Journal of Quality of Life Aspects of Treatment, Care & Rehabilitation. 2014;23(2):661-9.
108. Kaseva K, Dobewall H, Yang X, Pulkki-Råback L, Lipsanen J, Hintsa T, et al. Physical Activity, Sleep, and Symptoms of Depression in Adults - Testing for Mediation. Med Sci Sports Exerc. 2019;51(6):1162-8.
109. Kayani S, Kiyani T, Morris T, Biasutti M, Wang J. Physical activity and anxiety of chinese university students: Mediation of self-system. Int J Environ Res Public Health. 2021;18(9).
110. Kikkawa M, Shimura A, Nakajima K, Morishita C, Honyashiki M, Tamada Y, et al. Mediating Effects of Trait Anxiety and State Anxiety on the Effects of Physical Activity on Depressive Symptoms. Int J Environ Res Public Health. 2023;20(7).
111. Klussman K, Langer J, Nichols AL. The Relationship between Physical Activity, Health, and Well-Being: Type of Exercise and Self-Connection as Moderators. European Journal of Health Psychology. 2021;28(2):59-70.
112. Kong JY, Hong H, Kang H. Relationship between physical activity and depressive symptoms in older Korean adults: moderation analysis of muscular strength. BMC Geriatr. 2022;22(1).
113. Konopack JF, McAuley E. Efficacy-mediated effects of spirituality and physical activity on quality of life: A path analysis. Health and Quality of Life Outcomes. 2012;10.
114. Koziel Ly NK, Mohamud L, Villeneuve PJ, Matheson K, Anisman H, Chee MJ. Protective effects of physical activity on mental health outcomes during the COVID-19 pandemic. PLoS One. 2022;17(12 December).
115. Kratz AL, Ehde DM, Bombardier CH. Affective mediators of a physical activity intervention for depression in multiple sclerosis. Rehabil Psychol. 2014;59(1):57-67.
116. Kruk M, Zarychta K, Horodyska K, Boberska M, Scholz U, Radtke T, et al. What comes first, negative emotions, positive emotions, or moderate-to-vigorous physical activity? Ment Health Phys Act. 2019;16:38-42.
117. Ku PW, Steptoe A, Chen LJ. Prospective associations of exercise and depressive symptoms in older adults: the role of apolipoprotein E4. Qual Life Res. 2017;26(7):1799-808.
118. Kukihara H, Yamawaki N, Ando M, Tamura Y, Arita K, Nakashima E. The mediating effects of resilience, morale, and sense of coherence between physical activity and perceived physical/mental health among Japanese community-dwelling older adults: A cross-sectional study. Journal of Aging and Physical Activity. 2018;26(4):544-52.

119. La Rocque CL, Mazurka R, Stuckless TJR, Pyke K, Harkness KL. Randomized controlled trial of bikram yoga and aerobic exercise for depression in women: Efficacy and stress-based mechanisms. J Affect Disord. 2021;280:457-66.
120. Latimer AE, Martin Ginis KA, Hicks AL, McCartney N. An examination of the mechanisms of exercise-induced change in psychological well-being among people with spinal cord injury. J Rehabil Res Dev. 2004;41(5):643-51.
121. Lau SCL, Tabor Connor L, Baum CM. Motivation, Physical Activity, and Affect in Community-Dwelling Stroke Survivors: An Ambulatory Assessment Approach. Ann Behav Med. 2023.
122. Laurier C, Pascuzzo K, Beaulieu G. Uncovering the Personal and Environmental Factors Associated With Youth Mental Health During the COVID-19 Pandemic: The Pursuit of Sports and Physical Activity as a Protective Factor. Traumatology. 2021;27(4):354-64.
123. Leahy AA, Diallo TMO, Eather N, Duncan MJ, Smith JJ, Morgan PJ, et al. Mediating effects of sleep on mental health in older adolescents: Findings from the Burn 2 Learn randomized controlled trial. Scand J Med Sci Sports. 2023;33(11):2369-80.
124. Levante A, Quarta S, Massaro M, Calabriso N, Carluccio MA, Damiano F, et al. Physical activity habits prevent psychological distress in female academic students: The multiple mediating role of physical and psychosocial parameters. Heliyon. 2024;10(4).
125. Lewis R, Roden LC, Scheuermaier K, Gomez-Olive FX, Rae DE, Iacovides S, et al. The impact of sleep, physical activity and sedentary behaviour on symptoms of depression and anxiety before and during the COVID-19 pandemic in a sample of South African participants. Sci Rep. 2021;11(1).
126. Li Y, Sun Q, Sun M, Sun P, Xia X. Physical exercise and psychological distress: The mediating roles of problematic mobile phone use and learning burnout among adolescents. Int J Environ Res Public Health. 2021;18(17).
127. Li S, Wang X, Wang P, Qiu S, Xin X, Wang J, et al. Correlation of exercise participation, behavioral inhibition and activation systems, and depressive symptoms in college students. Sci Rep. 2023;13(1).
128. Li Z, Huang F. Mechanisms of the impact of sports participation on college students' psychological well-being. Soc Behav Pers. 2024;52(5).
129. Li B, Jiang W, Han SS, Ye YP, Li YX, Lou H, et al. Influence of moderate-to-high intensity physical activity on depression levels: a study based on a health survey of Chinese university students. BMC Public Health. 2024;24(1).

130. Li W, Gao Y, Liu G, Hao R, Zhang M, Li X. Shifting the Paradigm: A Fresh Look at Physical Activity Frequency and Its Impact on Mental Health, Life Satisfaction, and Self-Rated Health in Adolescents. International Journal of Mental Health Promotion. 2024;26(2):83-92.
131. Liang X, Qiu H, Sit CHP. The mediating role of resilience in the association between MVPA and psychological ill-being in children with ADHD. Scand J Med Sci Sports. 2023;33(4):485-94.
132. Lin ST, Hung YH, Yang MH. The Relationships among Sport Participation Level, Flow Experience, Perceived Health Status and Depression Level of College Students. Int J Environ Res Public Health. 2023;20(1).
133. Lin S, Li L, Zheng D, Jiang L. Physical Exercise and Undergraduate Students' Subjective Well-Being: Mediating Roles of Basic Psychological Need Satisfaction and Sleep Quality. Behav Sci (Basel). 2022;12(9).
134. Lindwall M, Larsman P, Hagger MS. The reciprocal relationship between physical activity and depression in older European adults: A prospective cross-lagged panel design using SHARE data. Health Psychol. 2011;30(4):453-62.
135. Lindwall M, Ljung T, Hadžibajramović E, Jonsdottir IH. Self-reported physical activity and aerobic fitness are differently related to mental health. Ment Health Phys Act. 2012;5(1):28-34.
136. Liu S, Ding M, Wang K, editors. The Effects of Physical Exercise on Anxiety Among College Students: The Mediating Role of Psychological Resilience2023.
137. Liu Y, Feng Q, Guo K. Physical activity and depression of Chinese college students: chain mediating role of rumination and anxiety. Front Psychol. 2023;14.
138. Liu M, Shi B. The effect of physical exercise on the anxiety of college students in the post-pandemic era: The mediating role of social support and proactive personality. Front Psychol. 2023;14.
139. Liu N, Zhong Q. The impact of sports participation on individuals’ subjective well-being: the mediating role of class identity and health. Humanities and Social Sciences Communications. 2023;10(1).
140. Liu R, Menhas R, Saqib ZA. Does physical activity influence health behavior, mental health, and psychological resilience under the moderating role of quality of life? Front Psychol. 2024;15.
141. Liu Y, Ge X, Wang Y, Qiao S, Cai Y. How race and socioeconomic status moderate the association between moderate-to-vigorous physical activity and depressive symptoms: a cross-sectional study with compositional data. Br J Sports Med. 2024.

142. Liu M, Liu H, Qin Z, Tao Y, Ye W, Liu R. Effects of physical activity on depression, anxiety, and stress in college students: the chain-based mediating role of psychological resilience and coping styles. Front Psychol. 2024;15.
143. Liu X, Du Q, Fan H, Wang Y. The impact of square dancing on psychological well-being and life satisfaction among aging women. Sci Rep. 2024;14(1).
144. Lopes MVV, Matias TS, da Costa BGG, Schuch FB, Chaput JP, Samara Silva K. The relationship between physical activity and depressive symptoms is domain-specific, age-dependent, and non-linear: An analysis of the Brazilian national health survey. J Psychiatr Res. 2023;159:205-12.
145. Mack DE, Meldrum LS, Wilson PM, Sabiston CM. Physical activity and psychological health in breast cancer survivors: An application of basic psychological needs theory. Applied Psychology: Health and Well-Being. 2013;5(3):369-88.
146. Maher JP, Pincus AL, Ram N, Conroy DE. Daily physical activity and life satisfaction across adulthood. Dev Psychol. 2015;51(10):1407-19.
147. Maher JP, Hevel DJ, Reifsteck EJ, Drollette ES. Physical activity is positively associated with college students' positive affect regardless of stressful life events during the COVID-19 pandemic. Psychol Sport Exerc. 2021;52.
148. Malek Rivan NF, Shahar S, Singh DKA, Ibrahim N, Mat Ludin AF, Yahya HM, et al. Mediation effect of coping strategies on general psychological health among middle-aged and older adults during the covid-19 pandemic. Aging & Mental Health. 2021.
149. Marselle MR, Irvine KN, Lorenzo-Arribas A, Warber SL. Does perceived restorativeness mediate the effects of perceived biodiversity and perceived naturalness on emotional well-being following group walks in nature? J Environ Psychol. 2016;46:217-32.
150. McIntyre E, Lauche R, Frawley J, Sibbritt D, Reddy P, Adams J. Physical activity and depression symptoms in women with chronic illness and the mediating role of health-related quality of life. J Affect Disord. 2019;252:294-9.
151. McNeil DG, Singh A, Chambers T. Exploring Nature-and Social-Connectedness as Mediators of the Relationship between Nature-Based Exercise and Subjective Wellbeing. Ecopsychology. 2022;14(4):226-34.
152. McPhie ML, Rawana JS. Unravelling the relation between physical activity, self-esteem and depressive symptoms among early and late adolescents: A mediation analysis. Ment Health Phys Act. 2012;5(1):43-9.
153. Meadows R, Bonner T, Dobhal M, Borra S, Killion JA, Paxton R. Pathways between physical activity and quality of life in African-American breast cancer survivors. Support Care Cancer. 2017;25(2):489-95.

154. Meckes SJ, McDonald MA, Lancaster CL. Association between physical activity and mental health among first responders with different service roles. Psychological Trauma: Theory, Research, Practice, and Policy. 2021;13(1):66-74.
155. Meyer S, Weidmann R, Grob A. The mirror's curse: Weight perceptions mediate the link between physical activity and life satisfaction among 727,865 teens in 44 countries. Journal of Sport and Health Science. 2021;10(1):48-54.
156. Meyer S, Lang C, Ludyga S, Grob A, Gerber M. “What If Others Think I Look Like…” The Moderating Role of Social Physique Anxiety and Sex in the Relationship between Physical Activity and Life Satisfaction in Swiss Adolescents. Int J Environ Res Public Health. 2023;20(5).
157. Miller BM, Bartholomew JB, Springer BA. Post-exercise affect: The effect of mode preference. J Appl Sport Psychol. 2005;17(4):263-72.
158. Moya CAM, Soares FC, Lima RA, de Barros MVG, Bezerra J. Depressive symptoms in older adults: the role of physical activity and social support. Trends Psychiatry Psychother. 2021.
159. Mu FZ, Liu J, Lou H, Zhu WD, Wang ZC, Li B. Influence of physical exercise on negative emotions in college students: chain mediating role of sleep quality and self-rated health. Frontiers in Public Health. 2024;12.
160. Mumba MN, Nancarrow A, Jaiswal JL, Hocchaus E, Campbell MH, Davis LL. Moderation Effects of Substance Use on Physical and Mental Well-Being in Adults. J Am Psychiatr Nurses Assoc. 2021.
161. Mumba MN, Nancarrow AF, Jaiswal JL, Hocchaus E, Campbell MH, Davis LL. Moderation Effects of Substance Use on Physical and Mental Well-Being in Adults. J Am Psychiatr Nurses Assoc. 2024;30(1):37-43.
162. Nezlek JB, Cypryańska M, Cypryański P, Chlebosz K, Jenczylik K, Sztachańska J, et al. Within-person relationships between recreational running and psychological well-being. J Sport Exerc Psychol. 2018;40(3):146-52.
163. O’Rourke RH, Sabiston CM, Trinh L, Arbour-Nicitopoulos KP. The indirect effects of basic psychological needs on the relationship between physical activity and mental health in adults with disabilities: A cross-sectional study. Advances in Rehabilitation. 2023;37(4):1-11.
164. Oberle E, Ji XR, Guhn M, Schonert-Reichl KA, Gadermann AM. Benefits of extracurricular participation in early adolescence: Associations with peer belonging and mental health. J Youth Adolesc. 2019;48(11):2255-70.
165. Olson RL, Brush CJ, Ehmann PJ, Alderman BL. A randomized trial of aerobic exercise on cognitive control in major depression. Clin Neurophysiol. 2017;128(6):903-13.

166. Oshimi D, Kinoshita K. Relationship between residents' sporting life and hedonic and eudaimonic well-being in Hiroshima: the mediating role of PERMA in sport. Managing Sport and Leisure. 2022.
167. Pacewicz CE, Rowley TW, Savage JL. The Role of Physical Activity on the Link Between Stress, Burnout, and Well-Being in Athletic Trainers. J Athl Train. 2022.
168. Pan Y, Zhou D, Shek DTL. After-School Extracurricular Activities Participation and Depressive Symptoms in Chinese Early Adolescents: Moderating Effect of Gender and Family Economic Status. Int J Environ Res Public Health. 2022;19(7).
169. Pascoe MC, Patten RK, Tacey A, Woessner MN, Bourke M, Bennell K, et al. Physical activity and depression symptoms in people with osteoarthritis-related pain: A cross-sectional study. PLOS Glob Public Health. 2024;4(7):e0003129.
170. Paxton RJ, Motl RW, Aylward A, Nigg CR. Physical activity and quality of life—The complementary influence of self-efficacy for physical activity and mental health difficulties. Int J Behav Med. 2010;17(4):255-63.
171. Perez-Sousa MA, Olivares PR, Gonzalez-Guerrero JL, Gusi N. Effects of an exercise program linked to primary care on depression in elderly: Fitness as mediator of the improvement. Quality of Life Research: An International Journal of Quality of Life Aspects of Treatment, Care & Rehabilitation. 2020.
172. Perez-Sousa MA, Pedro J, Carrasco-Zahinos R, Raimundo A, Parraca JA, Tomas-Carus P. Effects of Aquatic Exercises for Women with Rheumatoid Arthritis: A 12-Week Intervention in a Quasi-Experimental Study with Pain as a Mediator of Depression. Int J Environ Res Public Health. 2023;20(10).
173. Petruzzello SJ, Tate AK. Brain activation, affect, and aerobic exercise: An examination of both state-independent and state-dependent relationships. Psychophysiology. 1997;34(5):527-33.
174. Phillips SM, Wójcicki TR, McAuley E. Physical activity and quality of life in older adults: An 18-month panel analysis. Quality of Life Research: An International Journal of Quality of Life Aspects of Treatment, Care & Rehabilitation. 2013;22(7):1647-54.
175. Pickett K, Yardley L, Kendrick T. Physical activity and depression: A multiple mediation analysis. Ment Health Phys Act. 2012;5(2):125-34.
176. Precht LM, Margraf J, Stirnberg J, Brailovskaia J. It's all about control: Sense of control mediates the relationship between physical activity and mental health during the COVID-19 pandemic in Germany. Current Psychology. 2021.

177. Precht LM, Stirnberg J, Margraf J, Brailovskaia J. Can physical activity foster mental health by preventing addictive social media use? - A longitudinal investigation during the COVID-19 pandemic in Germany. J Affect Disord Rep. 2022;8:100316.
178. Quarta S, Levante A, García-Conesa MT, Lecciso F, Scoditti E, Carluccio MA, et al. Assessment of Subjective Well-Being in a Cohort of University Students and Staff Members: Association with Physical Activity and Outdoor Leisure Time during the COVID-19 Pandemic. Int J Environ Res Public Health. 2022;19(8).
179. Riddervold S, Haug E, Kristensen SM. Sports participation, body appreciation and life satisfaction in Norwegian adolescents: A moderated mediation analysis. Scandinavian Journal of Public Health. 2023.
180. Roppolo M, Mulasso A, Gollin M, Bertolotto A, Ciairano S. The role of fatigue in the associations between exercise and psychological health in Multiple Sclerosis: Direct and indirect effects. Ment Health Phys Act. 2013;6(2):87-94.
181. Rutherford ER, Vandelanotte C, Chapman J, To QG. Associations between depression, domain-specific physical activity, and BMI among US adults: NHANES 2011-2014 cross-sectional data. BMC Public Health. 2022;22(1).
182. Ryan MP. The antidepressant effects of physical activity: Mediating self-esteem and self-efficacy mechanisms. Psychology and Health. 2008;23(3):279-307.
183. Santino N, Larocca V, Hitzig SL, Guilcher SJT, Craven BC, Bassett-Gunter RL. Physical activity and life satisfaction among individuals with spinal cord injury: Exploring loneliness as a possible mediator. J Spinal Cord Med. 2020.
184. Santino N, Larocca V, Hitzig SL, Guilcher SJT, Craven BC, Bassett-Gunter RL. Physical activity and life satisfaction among individuals with spinal cord injury: Exploring loneliness as a possible mediator. J Spinal Cord Med. 2022;45(2):173-9.
185. Sato M, Jordan JS, Funk DC. A distance-running event and life satisfaction: The mediating roles of involvement. Sport Management Review. 2016;19(5):536-49.
186. Shang Y, Xie HD, Yang SY. The Relationship Between Physical Exercise and Subjective Well-Being in College Students: The Mediating Effect of Body Image and Self-Esteem. Front Psychol. 2021;12:658935.
187. Shang Y, Chen SP, Xie HD. The Effect of Physical Exercise on Subjective Well-Being in Chinese Middle School Students: The Mediation Roles of Peer Relationships and Self-Actualization. Asia-Pacific Education Researcher. 2024;33(3):615-23.
188. Shin M, Kim I, Kwon S. Effect of intrinsic motivation on affective responses during and after exercise: Latent curve model analysis. Percept Mot Skills. 2014;119(3):717-30.

189. Smith JJ, Beauchamp MR, Faulkner G, Morgan PJ, Kennedy SG, Lubans DR. Intervention effects and mediators of well-being in a school-based physical activity program for adolescents: The ‘Resistance Training for Teens’ cluster RCT. Ment Health Phys Act. 2018;15:88-94.
190. Smith KE, Mason TB, O'Connor SM, Wang S, Dzubur E, Crosby RD, et al. Bi-Directional Associations Between Real-Time Affect and Physical Activity in Weight-Discordant Siblings. J Pediatr Psychol. 2021;46(4):443-53.
191. Solberg PA, Halvari H, Ommundsen Y, Hopkins WG. A 1-year follow-up on effects of exercise programs on well-being in older adults. Journal of Aging and Physical Activity. 2014;22(1):52-64.
192. Stuntz CP, Grosshans M, Boghosian R, Brendel A, Williamson MS. Exert more and feel better, not worse?: Examining links among changes in exertion, feelings of accomplishment, and feeling states. Psychol Sport Exerc. 2020;48.
193. Sun D, Zhu X, Bao Z. The relationship between physical activity and anxiety in college students: exploring the mediating role of lifestyle habits and dietary nutrition. Front Psychol. 2024;15.
194. Syue SH, Yang HF, Wang CW, Hung SY, Lee PH, Fan SY. The Associations between Physical Activity, Functional Fitness, and Life Satisfaction among Community-Dwelling Older Adults. Int J Environ Res Public Health. 2022;19(13).
195. Taliaferro LA, Dodd VJ. Potential mediating pathways through which sports participation relates to reduced risk of suicidal ideation. Res Q Exerc Sport. 2010;81(3):328-39.
196. Tang S, Chen H, Wang L, Lu T, Yan J. The Relationship between Physical Exercise and Negative Emotions in College Students in the Post-Epidemic Era: The Mediating Role of Emotion Regulation Self-Efficacy. Int J Environ Res Public Health. 2022;19(19).
197. Tao B, Chen H, Lu T, Yan J. The Effect of Physical Exercise and Internet Use on Youth Subjective Well-Being—The Mediating Role of Life Satisfaction and the Moderating Effect of Social Mentality. Int J Environ Res Public Health. 2022;19(18).
198. Theodoropoulou E, Stavrou NAM, Karteroliotis K. Neighborhood environment, physical activity, and quality of life in adults: Intermediary effects of personal and psychosocial factors. Journal of Sport and Health Science. 2017;6(1):96-102.
199. Tian J, Yu H, Austin L. The Effect of Physical Activity on Anxiety: The Mediating Role of Subjective Well-Being and the Moderating Role of Gender. Psychol Res Behav Manag. 2022;15:3167-78.

200. Tiggelman D, van de Ven MOM, van Schayck OCP, Engels RCME. Moderating effect of gender on the prospective relation of physical activity with psychosocial outcomes and asthma control in adolescents: A longitudinal study. J Asthma. 2014;51(10):1049-54.
201. Tihanyi BT, Bör P, Emanuelsen L, Köteles F. Mediators between yoga practiceand psychological Well-Being mindfulness, body awareness and satisfaction with body image. European Journal of Mental Health. 2016;11(1-2):112-27.
202. Toups M, Carmody T, Greer T, Rethorst C, Grannemann B, Trivedi MH. Exercise is an effective treatment for positive valence symptoms in major depression. J Affect Disord. 2017;209:188-94.
203. Vandendriessche A, Ghekiere A, Van Cauwenberg J, De Clercq B, Dhondt K, Desmet A, et al. Does sleep mediate the association between school pressure, physical activity, screen time, and psychological symptoms in early adolescents? A 12-country study. Int J Environ Res Public Health. 2019;16(6).
204. VanKim NA, Nelson TF. Vigorous physical activity, mental health, perceived stress, and socializing among college students. Am J Health Promot. 2013;28(1):7-15.
205. Walsh RFL, Smith LT, Titone MK, Ng TH, Goel N, Alloy LB. The relationship between physical activity states and depressive symptoms: Using ambulatory assessment to characterize day-to-day associations among individuals with and without bipolar spectrum disorder. Depress Anxiety. 2022;39(12):835-44.
206. Walsh RFL, Smith LT, Klugman J, Titone MK, Ng TH, Goel N, et al. An examination of bidirectional associations between physical activity and mood symptoms among individuals diagnosed and at risk for bipolar spectrum disorders. Behav Res Ther. 2023;161:104255.
207. Wang GH, Li WD, Dou K. Extracurricular sports participation increases life satisfaction among Chinese adolescents: A moderated mediation model. Soc Behav Pers. 2020;48(8).
208. Wang K, Li Y, Zhang T, Luo J. The Relationship among College Students' Physical Exercise, Self-Efficacy, Emotional Intelligence, and Subjective Well-Being. Int J Environ Res Public Health. 2022;19(18).
209. Wassink-Vossen S, Collard RM, Penninx BW, Hiles SA, Oude Voshaar RC, Naarding P. The reciprocal relationship between physical activity and depression: Does age matter? Eur Psychiatry. 2018;51:9-15.
210. Watt T, Kehoe EJ. Toward a Mediated Model of Physical Activity in Relation to Psychological Distress. Physical Activity Review. 2022;10(1):52-9.
211. Wen CKF, Liao Y, Maher JP, Huh J, Belcher BR, Dzubur E, et al. Relationships among affective states, physical activity, and sedentary behavior in children: Moderation by perceived stress. Health Psychol. 2018;37(10):904-14.

212. Werneck AO, Silva DR, Malta DC, Lima MG, Souza-Júnior PRB, Azevedo LO, et al. The mediation role of sleep quality in the association between the incidence of unhealthy movement behaviors during the COVID-19 quarantine and mental health. Sleep Med. 2020;76:10-5.
213. Werneck AO, Stubbs B, Kandola A, Oyeyemi AL, Schuch FB, Hamer M, et al. Prospective Associations of Leisure-Time Physical Activity with Psychological Distress and Well-Being: A 12-Year Cohort Study. Psychosom Med. 2022;84(1):116-22.
214. Werneck AO, Cunha PM, Silva DR. The mediation role of social network size and perception in the association between physical activity and depressive symptoms: a prospective analysis from the SHARE study. Aging and Mental Health. 2023.
215. Werneck AO, Schuch FB, Felez-Nobrega M, Araujo RHO, Szwarcwald CL, Stubbs B, et al. Does occupation moderate the association between domain-based physical activity and depressive symptoms? Ment Health Phys Act. 2023;24.
216. White RL, Parker PD, Lubans DR, MacMillan F, Olson R, Astell-Burt T, et al. Domain-specific physical activity and affective wellbeing among adolescents: An observational study of the moderating roles of autonomous and controlled motivation. Int J Behav Nutr Phys Act. 2018;15.
217. White RL, Bennie J, Abbott G, Teychenne M. Work-related physical activity and psychological distress among women in different occupations: a cross-sectional study. BMC Public Health. 2020;20(1):1007.
218. Wichers M, Peeters F, Rutten BPF, Jacobs N, Derom C, Thiery E, et al. A time-lagged momentary assessment study on daily life physical activity and affect. Health Psychol. 2012;31(2):135-44.
219. Wilson KE, Das BM, Evans EM, Dishman RK. Structural equation modeling supports a moderating role of personality in the relationship between physical activity and mental health in college women. Journal of Physical Activity and Health. 2016;13(1):67-78.
220. Wu B, Xiong G, Zhang P, Ma X. Effects of tai chi, ba duan jin, and walking on the mental health status of urban older people living alone: the mediating role of social participation and the moderating role of the exercise environment. Frontiers in Public Health. 2024;12.
221. Wut TM, Lee SW, Xu JB. Mental Health of Working Adults during the COVID-19 Pandemic: Does Physical Activity Level Matter? Int J Environ Res Public Health. 2023;20(4).
222. Xin S, Ma X. Mechanisms of Physical Exercise Effects on Anxiety in Older Adults during the COVID-19 Lockdown: An Analysis of the Mediating Role of Psychological Resilience and the Moderating Role of Media Exposure. Int J Environ Res Public Health. 2023;20(4).

223. Xiong G, Wang C, Ma X. The Relationship between Physical Activity and Mental Depression in Older Adults during the Prevention and Control of COVID-19: A Mixed Model with Mediating and Moderating Effects. Int J Environ Res Public Health. 2023;20(4).
224. Yang L, Liu Z, Shi S, Dong Y, Cheng H, Li T. The Mediating Role of Perceived Stress and Academic Procrastination between Physical Activity and Depressive Symptoms among Chinese College Students during the COVID-19 Pandemic. Int J Environ Res Public Health. 2022;20(1).
225. Yao Y, Chen J, Dong D, Feng Y, Qiao Z. The Relationship between Exercise and Mental Health Outcomes during the COVID-19 Pandemic: From the Perspective of Hope. Int J Environ Res Public Health. 2022;19(7).
226. Yildirim S, ÖZgÖKÇE G. The Relationship Between Physical Activity and Life Satisfaction: The Mediating Role of Social-Physique Anxiety and Self-Esteem. Pamukkale Journal of Sport Sciences. 2023;14(3):346-67.
227. Yoshikawa E, Nishi D, Matsuoka YJ. Association between regular physical exercise and depressive symptoms mediated through social support and resilience in Japanese company workers: A cross-sectional study. BMC Public Health. 2016;16(1).
228. You S, Shin K, Kim M. Long-term effect of physical activity on internalizing and externalizing problems and life satisfaction. Sustainability (Switzerland). 2021;13(4):1-12.
229. You Y, Chen Y, Yin J, Zhang Z, Zhang K, Zhou J, et al. Relationship between leisure-time physical activity and depressive symptoms under different levels of dietary inflammatory index. Frontiers in Nutrition. 2022;9.
230. You Y, Wang R, Li J, Cao F, Zhang Y, Ma X. The role of dietary intake of live microbes in the association between leisure-time physical activity and depressive symptoms: a population-based study. Appl Physiol Nutr Metab. 2024.
231. Zeibig JM, Seiffer B, Frei AK, Takano K, Sudeck G, Rösel I, et al. Long-term efficacy of exercise across diagnostically heterogenous mental disorders and the mediating role of affect regulation skills. Psychol Sport Exerc. 2023;64.
232. Zhang Y, Zhang H, Ma X, Di Q. Mental health problems during the COVID-19 pandemics and the mitigation effects of exercise: A longitudinal study of college students in China. Int J Environ Res Public Health. 2020;17(10).
233. Zhang X, Pennell ML, Bernardo BM, Clark J, Krok-Schoen JL, Focht BC, et al. Body image, physical activity and psychological health in older female cancer survivors. J Geriatr Oncol. 2021;12(7):1059-67.

234. Zhang Z, Wang T, Kuang J, Herold F, Ludyga S, Li J, et al. The roles of exercise tolerance and resilience in the effect of physical activity on emotional states among college students. Int J Clin Health Psychol. 2022;22(3).
235. Zhang B, Lei SM, Le S, Gong Q, Cheng S, Wang X. Changes in health behaviors and conditions during COVID-19 pandemic strict campus lockdown among Chinese university students. Front Psychol. 2022;13.
236. Zhang J, Zheng S, Hu Z. The Effect of Physical Exercise on Depression in College Students: The Chain Mediating Role of Self-Concept and Social Support. Front Psychol. 2022;13.
237. Zhang X, Feng S, Peng R, Li H. Using Structural Equation Modeling to Examine Pathways between Physical Activity and Sleep Quality among Chinese TikTok Users. Int J Environ Res Public Health. 2022;19(9).
238. Zhang X, Wang D, Li F. Physical Exercise, Social Capital, Hope, and Subjective Well-Being in China: A Parallel Mediation Analysis. Int J Environ Res Public Health. 2022;20(1).
239. Zhang M, Xu X, Jiang J, Ji Y, Yang R, Liu Q, et al. The association between physical activity and subjective well-being among adolescents in southwest China by parental absence: a moderated mediation model. BMC Psychiatry. 2023;23(1).
240. Zhao Y. The Effect of Physical Exercise on College Students' Mental Health and General Self-efficacy. Revista de Psicologia del Deporte. 2021;30(4):167-74.
241. Zhao H, Zhang B, Liu W, Jiang Y. The relationship between physical activity and depression in college students: the chain mediating role of mindfulness and meaning in life. Current Psychology. 2024.
242. Zheng Y, Wen P, Wu J, Jia H, Lai D, Xun Y, et al. Physical exercise and anxiety: The chain mediating effect of social support and resilience. Soc Behav Pers. 2024;52(6).
243. Zhou GY, Yang B, Li H, Feng QS, Chen WY. The influence of physical exercise on college students’ life satisfaction: The chain mediating role of self-control and psychological distress. Front Psychol. 2023;14.
244. Zhou Y, Guo K. Physical activity and depression: A chain mediation model. Soc Behav Pers. 2023;51(10).
245. Zhu JH, Li SF, Wang P, Xin X, Zhao Q, Chen SC, et al. Correlation and pathways of behavioral activation systems mediating physical activity level and depressive symptoms among college students. World J Psychiatry. 2023;13(10):784-92.
246. Zou Y, Meng F, Yan X. The combined association of adherence to recommended physical activity and glycemic control with depression: an exploratory study with mediation and moderation models. BMC Public Health. 2023;23(1).

247. Zuo Y, Zhang M, Han J, Chen KW, Ren Z. Residents' physical activities in home isolation and its relationship with health values and well-being: A cross-sectional survey during the COVID-19 social quarantine. Healthcare (Switzerland). 2021;9(7).
