# Supplementary material for: Physical activity and mental health: a systematic review and best-evidence synthesis of mediation and moderation studies
Source: Int J Behav Nutr Phys Act. 2024 Nov 28;21:134. doi: 10.1186/s12966-024-01676-6 (PMC11603721; doi:10.1186/s12966-024-01676-6)
Supplement: Supplementary file 3 — Additional file 3. Study characteristics Table 14AUG24. Study Characteristics Table. A table presenting characteristics of each included study. [file 12966_2024_1676_MOESM3_ESM.pdf]

Table S1

Study Characteristics.

Study ID	First Author	Year	Country	Study Design	Sample Size	Participant Description	Age (Range)	Age (M)	Age (SD)	Sex (% Male)
1	Adams	2018	UK	Experimental	63	Testicular cancer survivors	21-58	43.7	10.8	100.0%
2	Aguinaga	2018	United States	Experimental	307	Low-active older adults		70.6	5.0	28.5%
3	Alfini	2020	United States	Experimental	32	Older adults	55–81	66.3	7.3	25.0%
4	Annesi	2018	United States	Experimental	15	Adolescent candidates for Bariatric Surgery	12-18	15.1	1.7	
5	Annesi	2019	United States	Experimental	41	People participating in Parkinson Disease tailored physical activity classes		71.1	7.0	46.3%
6	Asiamah	2022	Africa	Cross sectional	905	full-time African academics	50+	56.3	5.4	68.5%
7	Asztalos	2015	Belgium	Cross Sectional	4,344	Adults	25-64	43.6	11.1	47.8%
8	Awick	2017	United States	Experimental	247	Older adults	60-79	65.7	4.6	71.6%
9	Backström-Eriksson	2016	Sweden	Cross-sectional	68	Patients with Cystic Fibrosis	18-67	32.2	11.1	54.0%
10	Bae	2024	Korea	Cross sectional	2142	Elementary school students	11-13		7.7	50.0%
11	Baker	2023	Australia	Cross sectional	1876	Women	18-45	36.0	0.7	0.0%
12	Bang	2024	United States	Cross-sectional	4751	Adolescents	Grades 7-12			48.4%
13	Barham	2022	United States	Cross Sectional	1,576	Adults	18-78	39.3	13.7	60.0%
14	Barr	2010	Canada	Experimental	24	Physically active men	18-29	22.5	3.4	100.0%
15	Bartholomew	1998	United States	Experimental	20	Undergraduate students in exercise science classes		21.5 (males) 22.9 (females)	2.42 (males) 2.81 (females)	50.0%
16	Baruth	2016	United States	Cross Sectional	315	Adults		57.0 (intervention) 56.4 (control)	9.9 (intervention) 10.8 (control)	12.4%
17	Berger	1987	United States	Experimental	48	College students	18-59	25.3		48.0%
18	Bhandari	2020	India	Cross-sectional	10,604	Older adults	>60			47.4%
19	Biese	2024	United States	Cross-sectional	4874	Adolescent athletes	16.1	1.3		
20	Birch	2016	United States	Cross-sectional	501	Older adults	52-102	81.8	10.6	30.0%
21	Bodin	2001	Sweden	Experimental	12	Regular runners	26-46	39.7	6.1	50.0%
22	Booij	2014	Netherlands	Longitudinal	715	Adolescents	11-21	13.5 (T2), 16.2 (T3)	0.5 (T2), 0.6 (T3)	41.9%
23	Borges	2022	United states	Experimental	195	Smokers	18-65	45.3	11.0	28.0%
24	Brady	2021	UK	Cross Sectional	345	Rheumatoid arthritis patients		51.5	11.7	7.0%
25	Brière	2018	Canada	Prospective	17,550	Adolescents		14.4	1.3	46.0%
26	Broman-Fulks	2018	United states	Experimental	955	Adults	18-85	45.8	16.2	39.2%
27	Buffart	2014	Netherlands	Experimental	209	Cancer survivors	≥18	48.8 (exercise), 51.3 (waitlist)	10.9 (exercise), 8.8 (waitlist)	Exercise: 16.3%, Waitlist: 9.7%
28	Castan	2024	Spain	Cross-sectional	159	Adults with spinal cord injury		46.9	10.8	78.0%
29	Cecchini	2019	Spain	Cross Sectional	335	College students	19-29	20.1	2.4	49.9%

Study ID	First Author	Year	Country	Study Design	Sample Size	Participant Description	Age (Range)	Age (M)	Age (SD)	Sex (% Male)
30	Chae	2016	South Korea	Cross Sectional	848	High School students	15-18			41.7%
31	Chair	2020	Hong Kong	Cross Sectional	593	Patients discharged from hospitals after a cardiac event or community-dwelling people with coronary heart disease	18+	56.9	12.5	66.0%
32	Chan	2018	Hong Kong and UK	Cross Sectional	349	Older adults	50+	61.5	8.5	45.0%
33	Chan	2024	Scotland	Longitudinal	520	Older adults		76.2		50.4%
34	Chang	2020	Taiwan	Cross Sectional	1,114	Adults		35.9	15.2	46.1%
35	Chen	2019	United States	Cross Sectional	6,044	Older adults		64.3	10.4	46.0%
36	Chen	2022	China	Cross sectional	419	Older adults		72.5	1.6	47.0%
37	Cho	2017	United States	Cross Sectional	70	College students		20.9	1.7	
38	Choi	2024	United States	Cross sectional	5593	Older adults	65+		0.6	45.2%
39	Chu	2023	China	Cross sectional	235	Breast cancer survivors	32-97	64.2	10.9	0.0%
40	Clement	2024	Canada	Longitudinal	454	Adolescents		20.0	3.5	33.7%
41	Condello	2016	Italy	Cross Sectional	179	Adults	55-84			57.5%
42	Conley	2020	United States	Cross Sectional	4,520	Children	9-10	9.9	0.6	52.5%
43	Costigan	2019	Australia	Cross Sectional	1,223	High School students		12.9	0.5	55.1%
44	Dahlstrand	2021	Australia	Cross sectional	1,284	Adolescents	13	13.6	0.4	41.4%
45	Dang	2023	Canada	Cross sectional	22	Patients from the Centre for Addiction and Mental Health	18-30	24.8	2.3	45.0%
46	Deng	2016	United States	Cross Sectional	4,982	Young adults	24-34			46.1%
47	Deng	2018	China	Cross Sectional	5,949	Middle-aged and older adults	45+			46.0%
48	Deng	2023	China	Cross sectional	353	undergraduate students		20.1	1.7	41.4%
49	Dong	2022	China	Cross Sectional	248	College students				
50	Dong	2023	China	Cross sectional	1491	Adolescents		14.9	1.1	50.5%
51	Donyaei	2023	Iran	Experimental	34	Women with type 2 diabetes		60.6	6.3	0.0%
52	Doré	2020	Canada	Cross Sectional	937	Children	10-11	10.3	0.6	45.0%
53	Dotson	2016	United States	Experimental	396	Older adults	70-89			
54	Dunton	2015	United States	EMA	116	Adults		40.3	9.5	27.0%
55	Eddolls	2018	UK	Cross Sectional	314	Children		12.5	1.1	100.0%
56	Elavsky (a)	2005	United States	Cross Sectional	133	Women	44-60	51.1	4.1	0.0%
57	Elavsky (b)	2005	United States	Cross Sectional	174	Sedentary older adults		66.7	5.4	28.0%
58	Elavsky	2009	United States	Cross Sectional	212	Peri- and post-menopausal women		56.7	3.9	0.0%

Study ID	First Author	Year	Country	Study Design	Sample Size	Participant Description	Age (Range)	Age (M)	Age (SD)	Sex (% Male)
59	Evans	2017	United States	Cross Sectional	173	Physically active healthy adults		37.6	13.4	23.1%
60	Fauth	2007	United States	Longitudinal	1,315	Youth	9-12	10.7 (wave 1)	1.5 (wave 1)	50.4%
61	Feng	2024	China	Cross sectional	359	Secondary school students	12-16	13.7	1.6	55.7%
62	Fernandes	2024	Portugal	Cross sectional	1053	Adolescents	12-18	14.4	10.0	41.9%
63	Fessler	2023	28 European countries and Israel	Cross sectional	121875	Adults		67.0	2.2	45.0%
64	Feuerhahn	2012	Germany	A day-level study	126	Adults		37.8	10.4	32.5%
65	Fisher	2017	United States	Longitudinal	240	Women with non-metastatic stage 0-III breast cancer		50.3	9.0	0.0%
66	Fontana	2022	United States	Cross sectional	635	teachers		42.0	18.0	25.4%
67	Foroughi	2023	Iran	Cross sectional	200	University students		24.3		36.0%
68	Forshaw	2023	Australia	Cross-sectional	97	University students	19=33	21.8		49.5%
69	Fredericks	2006	United States	Longitudinal	1,042	Year 11 students				49.0%
70	Geniole	2016	Canada	Experimental	31	Young adults		24.6	3.9	100.0%
71	Gerber	2014	Switzerland	Longitudinal	580	Physically active vocational students		17.8	1.3	66.2%
72	Giacobbi	2006	United States	Cross Sectional	48	Individuals with physical disabilities with experience playing wheelchair sports	18-44	27.9	6.0	72.9%
73	Gianotta	2023	Sweden	Longitudinal	910	Adolescents	T1 = 12-13, T2 = 15-16, T3 = 18-19		5.2	44.0%
74	Ginis	2003	Canada	Experimental	34	Individuals with spinal cord injury	18+	38.6	11.7	67.7%
75	Ginoux	2021	France	Cross Sectional	405	Adults (during Covid lockdown)		34.1	14.2	31.2%
76	Goldstein	2020	United States	Experimental	413	Adults	30-69	49.6	11.6	24.0%
77	Gomez-Baya	2017	Spain	Cross Sectional	1,810	Adolescents	13-18	14.6	1.3	49.1%
78	González-Hernández	2019	Spain	Cross Sectional	436	Adolescents	14-19	16.8	0.8	67.4%
79	Görgülü	2021	Germany	Experimental	76	Patients with major depressive disorder		47.6	12.2	25.0%
80	Greenleaf	2009	United States	Cross Sectional	260	Undergraduate students	17-25	18.3	0.8	0.0%
81	Guérin	2013	United States	Experimental	41	Female runners		41.0	4.9	0.0%
82	Guicciardi	2019	Italy	Cross Sectional	28	Adults with multiple sclerosis	26-74	51.6	14.9	35.7%
83	Gujral	2024	Australia	Experimental	99	Older adults	60-80	69.1	1.1	45.5%
84	Guo	2024	China	Cross sectional	1100	College students		19.7	11.9	55.6%
85	Gyasi	2019	Ghana	Cross Sectional	1,200	Older adults	50-111	66.2	11.9	36.8%
86	Gyasi	2024	Ghana	Cross sectional	1201	Older adults from low and middle income	50+	66.1	1.9	36.7%
87	Hachenberger (a)	2022	Germany	EMA	147	Adults	18-25	22.6	4.4	14.3%

Study ID	First Author	Year	Country	Study Design	Sample Size	Participant Description	Age (Range)	Age (M)	Age (SD)	Sex (% Male)
88	Hachenberger (b)	2022	Germany	EMA	90	University students		24.0	6.6	18.9%
89	Halliday	2019	Australia	Cross Sectional	1,756	Adolescents		14.5	1.3	51.9%
90	Han	2023	China	Cross Sectional	251	College students		20.7	1.2	57.8%
91	Hayes	1986	United States	Cross Sectional	401	Adults	18-83			
92	Herring	2021	Ireland	Cross Sectional	470	Young adults	18-40	23.2	4.8	36.6%
93	Herzog	2022	Germany	Experimental	88	People with moderate or high anxiety		23.7	5.1	31.0%
94	Ho	2015	Hong Kong	Cross Sectional	775	School students	12-14	12.3	1.3	
95	Hogan	2013	United States	Experimental	144	Adults	19-93	51.3 (exercisers) 50.8 (control)	21.7 (exercisers) 20.0 (control)	Exercise: 47.9%, Control: 50.7%
96	Hogan	2015	United States	Longitudinal	624	Adults	19-65	40.9	9.6	46.3%
97	Hou	2024	China	Cross sectional	3240	Older adults	60+	68.1	1.8	53.4%
98	Huang	2021	United States	Cross Sectional	7728	Adults	20+			
99	Huang	2022	Taiwan	Cross sectional	197	Older diabetes patients	>65			45.7%
100	Hunt-Shanks	2009	Canada	Longitudinal	801	Cardiac patients				75.4%
101	Jenkins	2021	New Zealand	Cross Sectional	759	Adults	18-81	43.0	13.7	26.2%
102	Jenkins	2022	New Zealand	Cross Sectional	262	Adults	20-73	44.8	13.8	28.6%
103	Ji	2024	China	Cross-sectional	6195	Secondary school students	10-19	15.1	1.8	51.3%
104	Jia	2023	Thailand	Cross sectional	32	Obese primary school students		11.5	11.8	50.0%
105	Johansson	2011	Sweden	Experimental	20	University students	20-29	22.4 (females) 24.2 (males)	3.0 (females) 2.81 (males)	50.0%
106	Johnson	2022	United States	Experimental	80	University students	18-34	21.7	2.7	32.5%
107	Joseph	2013	United States	Cross Sectional	590	Undergraduate students		20.4 (males) 20.2 (females)	1.9 (males) 1.4 (females)	51.5%
108	Kaseva	2019	Finland	Longitudinal	3,596	Adults		38.1	5.0	49.1%
109	Kayani	2021	China	Cross Sectional	305	University students	18-36			60.7%
110	Kikkawa	2023	Japan	Cross sectional	526	Adults	20-77	41.1	0.5	43.3%
111	Klussman	2021	United States	Cross Sectional	143	Adults		34.5	11.2	33.3%
112	Kong	2022	Korea	Longitudinal	9920	Older adults	65+	73.4	6.5	40.7%
113	Konopack	2012	United States	Cross Sectional	164	Adults	50-84	66.6	9.4	23.7%
114	Kozielly	2022	Canada and the United States	Longitudinal	319	Adults	18+			20.7% (T2)
115	Kratz	2014	United States	Longitudinal	92	Multiple sclerosis patients	18-70	47.1 (intervention) 49.7 (control)	8.9 (intervention) 7.9 (control)	Interventi on: 11%, Control: 17% control
116	Kruk	2019	Poland	Longitudinal	879	Adults	23-68	36.7	6.1	17.1%
117	Ku	2017	Taiwan	Cross Sectional	639	Adults	64+			56.6%

Study ID	First Author	Year	Country	Study Design	Sample Size	Participant Description	Age (Range)	Age (M)	Age (SD)	Sex (% Male)
118	Kukihara	2018	Japan	Cross Sectional	369	Older adults	60-96	74.1	7.3	46.2%
119	La Rocque	2021	United States	Experimental	53	Women	18-65			0.0%
120	Latimer	2004	Canada	Experimental	21	Individuals with traumatic spinal cord injury	19-65	38.3 (intervention) 43.1 (control)	10.0 (intervention) 11.0 (control)	23.8%
121	Lau	2023	United States	EMA	40	Community dwelling stroke survivors	35-65	52.8	7.5	57.5%
122	Laurier	2021	Quebec	Cross sectional	113	Adolescents	Nov-17	15.3	1.5	32.3%
123	Leahy	2023	Australia	Experimental	292	Adolescents		16.0		51.0%
124	Levante	2024	Italy	Cross sectional	411	University students	18-24		2.3	0.0%
125	Lewis	2021	South Africa	Cross Sectional	1,048	adults		32.8	14.4	25.4%
126	Li	2021	China	Cross sectional	2,077	Adolescents	10-19	16.3	1.0	13.5%
127	Li	2023	China	Cross sectional	2606	College students	18-22	19.3	1.4	66.8%
128	Li, Z.	2024	China	Cross sectional	363	College students	18-23	21.7		51.0%
129	Li, B.	2024	China	Cross sectional	41620	College students			1.4	41.4%
130	Li, W.	2023	China	Cross-sectional	3578	High School students	15-18			48.7%
131	Liang (a)	2022	Hong Kong	Cross Sectional	85	Children with ADHD	6-12	8.4	1.4	84.7%
132	Lin (a)	2022	Taiwan	Cross Sectional	605	University students				52.9%
133	Lin (b)	2022	China	Cross sectional	770	Undergrad students	18-22	19.9	1.2	39.7%
134	Lindwall	2011	11 European countries	Longitudinal	17,593	Older adults		64.1	9.6	45.4%
135	Lindwall	2012	Sweden	Cross Sectional	177	Adults		39.1	8.1	49.0%
136	Liu, S.	2023	China	Cross sectional	1658	College students			1.2	34.4%
137	Liu, Y.	2023	China	Cross sectional	1292	College students	18-24	19.4		50.3%
138	Liu, M.	2023	China	Cross sectional	577					31.0%
139	Liu, N.	2023	China	Cross sectional	3501			48.1		
140	Liu, R.	2024	China	Cross sectional	743	Adults	21+		0.8	52.1%
141	Liu, Y.	2024	United States	Cross sectional	2803	Adults	20-85	48.0		49.2%
142	Liu, M.	2024	China	Cross-sectional	1380	Undergraduate students	17-25			56.0%
143	Liu, X.	2024	China	Cross sectional	2311	Middle-aged and elderly women	45+			0.0%
144	Lopes	2023	Brazil	Cross sectional	89,923	Older adolescents and adults	15+			47.6%
145	Mack	2013	Canada	Longitudinal	144	Female breast cancer survivors		55.1	10.9	0.0%
146	Maher	2015	United States	Daily diary study	150	Adults	18-89	47.5	18.6	49.0%
147	Maher	2021	United States	Cross Sectional	107	Undergraduate students	18-34	21.7	2.6	Approximately One third
148	MalekRivan	2021	Malaysia	Cross Sectional	535	middle aged and older adults	52+	71.2	5.7	44.5%

Study ID	First Author	Year	Country	Study Design	Sample Size	Participant Description	Age (Range)	Age (M)	Age (SD)	Sex (% Male)
149	Marselle	206	England	Longitudinal	127	Regular walkers	55-64			44.5%
150	McIntyre	2019	Australia	Cross Sectional	1,932	Women with chronic illness	53-95	68.7	8.8	0.0%
151	McNeil	2022	Australia	Cross sectional	359	Adults	18-80	40.2	16.2	35.9%
152	McPhie	2012	United States	Cross Sectional	4,204	Adolescents		14.7 (early adolescents) 16.2 late adolescents		49.8%
153	Meadows	2017	United States	Cross Sectional	135	Breast cancer survivors	55+	63.0		0.0%
154	Meckes	2020	United States	Cross Sectional	164	First responders	18+			72.6%
155	Meyer	2021	44 countries	Longitudinal	727,865	Teenagers	10-17	13.6		48.7%
156	Meyer	2023	Switzerland	Longitudinal	864	Adolescents	16-25	17.9		57.0%
157	Miller	2005	United States	Experimental	34	Female undergraduate students		20.6	2.5	0.0%
158	Moya	2021	Brazil	Cross Sectional	397	Older adults		72.8	8.1	22.7%
159	Mu	2024	China	Cross sectional	30475	College students				
160	Mumba	2021	United states	Cross Sectional	450016	Adults	18+			44.2%
161	Mumba	2024	United States	Cross sectional	450016	Adults	18+		11.5	44.2%
162	Nezlek	2018	Poland	Weekly diary study	244	Regular runners		32.5	7.9	52.0%
163	Oberle	2019	Canada	Longitudinal	3,045	Elementary and secondary school students		9.2 (grade 4) 12.3 (grade 7)	0.5 (grade 4) 0.5 (grade 7)	50.0%
164	Olson	2017	United States	Experimental	30	Adults with major depressive disorder		21.1	2.0	20.0%
165	O'Rourke	2023	Canada	Cross sectional	100	Adults with a disability	18-61	36.6	10.7	46.0%
166	Oshimi	2022	Japan	Cross Sectional	946	Adults		0.5	16.9	47.4%
167	Pacewicz	2022	United States	Cross sectional	163	Athletic trainers	22-60	30.5	6.7	22.1%
168	Pan	2022	China	Cross sectional	9,672	Early adolescents		14.5	0.7	
169	Pascoe	2023	Australia	Cross sectional	552	Adults with osteoarthritis-related pain		62.6	0.5	45.3%
170	Paxton	2010	United States	Cross Sectional	196	Older adults		74.0	8.3	25.0%
171	Perez-Sousa	2020	Spain	Experimental	2,768	Middle aged and older adults with depression		70.2 (control) 68.4 (intervention)	8.3 (control), 7.0 (intervention)	Control: 13.5%, Intervention: 7.6%
172	Perez-Sousa	2023	Spain	Experimental	43	Women with Rheumatoid Arthritis	18-80			0.0%
173	Petruzzello	1997	United States	Experimental	20	University students		22.6	3.3	75.0%
174	Phillips	2013	United States	longitudinal	321	Community-dwelling adults		63.8		19.9%
175	Pickett	2012	United Kingdom	Cross Sectional	164	Adults with depression or low mood	18-65			36.0%
176	Precht	2021	Germany	Cross Sectional	568	University students	16-66	19.9	4.5	31.0%
177	Precht	2022	Germany	Longitudinal	356	Adults		27.5	6.6	

Study ID	First Author	Year	Country	Study Design	Sample Size	Participant Description	Age (Range)	Age (M)	Age (SD)	Sex (% Male)
178	Quarta	2022	Italy	Cross Sectional	1,777	Adults	18+			29.3%
179	Riddervold	2023	Norway	Cross sectional	541	Late adolescents	16-19	16.9	1.1	56.0%
180	Roppolo	2013	Italy	Experimental	35	Women with multiple sclerosis	22-50	40.0	5.0	0.0%
181	Rutherford	2022	United States	Cross Sectional	10,047	Adults	20+	46.4		49.2%
182	Ryan	2008	United States	Cross Sectional	381	Undergraduate students	18-25	19.5 (males) 18.9 (females)	1.6 (males) 1.44 (females)	49.3%
183	Santino	2020	Canada	Cross Sectional	170	Community dwelling individuals				80.0%
184	Santino	2022	Canada	Cross sectional	170	Community dwelling individuals	18+			80.0%
185	Sato	2016	United states	Experimental	742	Runners	21-71	38.0		33.0%
186	Shang	2021	China	Cross sectional	671	College students				77.6%
187	Shang	2023	China	Cross sectional	1056	Middle school students	11-15	13.3		48.9%
188	Shin	2014	Korea	Experimental	30	Active college students		21.7	1.6	100.0%
189	Smith	2018	Australia	Experimental	508	Adolescents		14.1	0.5	50.4%
190	Smith	2021	United States	EMA	77	Weight discordant siblings		15.4	1.4	58.4%
191	Solberg	2014	Norway	Longitudinal	62	Older adults		75.0	5.0	39.0%
192	Stuntz	2020	United States	Cross Sectional	122	Athletes	18-22	19.7	1.3	61.5%
193	Sun	2024	China	Cross sectional	236	College students	18-20		13.3	52.6%
194	Syue	2022	Taiwan	Cross Sectional	623	Older adults	65+	73.7	5.9	39.8%
195	Taliaferro	2013	United States	Cross Sectional	450	Undergraduate students	18-24	19.0	1.5	26.0%
196	Tang	2022	China	Cross sectional	479	College students		19.9	1.3	61.2%
197	Tao	2022	China	Cross Sectional	619	Young people	18-35			43.9%
198	Theodoropoulou	2017	Greece	Cross Sectional	684	Adults	18-65	39.2	13.5	30.1%
199	Tian	2022	China	Cross Sectional	1153	College students		20.0	1.0	72.3%
200	Tiggelman	2014	Netherlands	Cross Sectional	253	Adolescents with asthma	10-14	13.9	1.1	58.8%
201	Tihanyi	2015	Hungary	Cross Sectional	203	Advanced yoga participants	19-68	36.8	10.0	9.9%
202	Toups	2017	United States	Experimental	126	Adults with major depressive disorder	18-70	46.9	10.1	18.5%
203	Vandendriessche	2019	12 countries	Cross Sectional	49,403	Children		13.7	1.6	48.1%
204	VanKim	2013	United States	Cross Sectional	14,804	Undergraduate college students	18-24+			36.9%
205	Walsh	2022	United States	EMA	111	Young adults	18-27	22.3	2.2	39.6%
206	Walsh	2023	United States	EMA	112	Young adults		22.0	2.2	39.0%
207	Wang	2020	China	Cross Sectional	1,608	Middle school students	11-19	14.5	1.8	43.9%
208	Wang	2022	China	Cross Sectional	826	College students		20.1	1.1	46.1%
209	Wassink-Vossen	2018	Netherlands	Longitudinal	1,079	Patients with major depressive disorder	18-88			

Study ID	First Author	Year	Country	Study Design	Sample Size	Participant Description	Age (Range)	Age (M)	Age (SD)	Sex (% Male)
210	Watt	2022	Australia	Cross sectional	394	Psychology university students				40.1%
211	Wen	2018	United States	longitudinal observational EMA study	180	Children	8-12	9.6	0.9	48.3%
212	Werneck	2020	Brazil	Cross Sectional	45,161	Adults	18+			
213	Werneck	2022	UK	Longitudinal	5,197	Adults		34 (T1), 42 (T2)		51.7%
214	Werneck (a)	2023	Europe	Longitudinal	10,569	Middle aged and older adults		67.5	8.3	43.4%
215	Werneck (b)	2023	Brazil	Cross sectional	51,895	Adults		40.7		55.2%
216	White	2020	Australia	Cross Sectional	1,080	Women	18-65	41.2	11.7	0.0%
217	White	2018	Australia	Cross Sectional	1,632	Adolescents	11-15	12.9	0.5	55.0%
218	Wichers	2012	Belgium	Time-lagged momentary assessment strategy	504	Female twins	18-46	27.0	7.6	0.0%
219	Wilson	2016	United states	Cross Sectional	298	Fist year university students	18-20	18.3	0.5	0.0%
220	Wu	2024	China	Cross sectional	1027	Older adults	60+		8.2	43.1%
221	Wut	2023	Hong Kong	Cross Sectional	109	Working adults	18+	46.0		40.0%
222	Xin	2023	China	Cross Sectional	451	Older adults	60+	64.0	1.3	46.3%
223	Xiong	2023	China	Cross sectional	974	Older adults	60+			50.3%
224	Yang	2022	China	Cross sectional	586	College students		20.3	1.8	57.0%
225	Yao	2022	China	Cross sectional	2,390	Older adolescents and adults	16-64	23.7	8.8	31.1%
226	Yildirim	2023	Turkey	Cross sectional	334	Adults	18-45	29.4	1.7	47.9%
227	Yoshikawa	2016	Japan	Cross Sectional	715	Employees		39.9	9.4	83.4%
228	You	2021	South Korea	Longitudinal	2,092	Middle school students	12-14	13.0	0.8	52.5%
229	You	2022	United States	Cross Sectional	11,078	Adults		44.0		51.38%
230	You	2024	China	Cross-sectional	25747	Adults		49.7		49.3%
231	Zeibig	2023	Germany	Experimental	72	Adults	18-65			27.8%
232	Zhang	2020	China	Longitudinal	66	College students		20.7	2.1	37.9%
233	Zhang	2021	United States	Longitudinal	4,567	Female cancer survivors	66-98	78.4	5.7	0.0%
234	Zhang, Z.	2022	China	Cross sectional	1,117	College students	18-25	18.9	1.3	49.6%
235	Zhang, B.	2022	China	Cross sectional	2,084	University students	18-35	22.4		61.1%
236	Zhang, J.	2022	China	Cross Sectional	1200	College students	17-24	21.5	0.8	41.0%
237	Zhang, X. (a)	2022	China	Cross sectional	660	Tik tok users				55.5%
238	Zhang, X. (b)	2022	China	Cross sectional	4,031	Adults				46.4%
239	Zhang	2023	China	Cross sectional	3143	Adolescents		12.9		47.2%
240	Zhao	2021	China	Cross Sectional	366	College students				51.1%

Study ID	First Author	Year	Country	Study Design	Sample Size	Participant Description	Age (Range)	Age (M)	Age (SD)	Sex (% Male)
241	Zhao	2024	China	Cross sectional	954	College students				53.0%
242	Zheng	2024	China	Cross sectional	325					55.8%
243	Zhou	2023	China	Cross Sectional	526	College students	17-19	18.8	1.7	38.4%
244	Zhu	2023	China	Cross sectional	442	College Students	18-26		1.2	
245	Zhuo	2023	China	Cross sectional	1605	College students	18-23	20.8		50.3%
246	Zou	2023	United States	Cross sectional	31302	Adults		Without depression = 46.9; with depression = 46.4	Without depression = 0.3; with depression = 0.4	49.3%
247	Zuo	2021	China	Cross Sectional	505	Adults	18-60			44.4%
