# Supplementary material for: Physical activity and mental health: a systematic review and best-evidence synthesis of mediation and moderation studies
Source: Int J Behav Nutr Phys Act. 2024 Nov 28;21:134. doi: 10.1186/s12966-024-01676-6 (PMC11603721; doi:10.1186/s12966-024-01676-6)
Supplement: Supplementary file 4 — Additional file 4. Results tables 16AUG24. Results tables. A file including the individual results extracted for each included study. [file 12966_2024_1676_MOESM4_ESM.pdf]

Table S2

Mediators in the relationship between physical activity and mental health.

	First Author	Year	Participants	Physical activity		Mental Health		Mediator		Mediation Results	Coefficients			Sig. med
				Variable	Measure	Variable	Measure	Variable	Measure		x → m	m → y	x → y	
**PSYCHOLOGICAL MEDIATORS**														
**Affect, wellbeing, stress, and depression or anxiety symptomology or sensitivity**	You [228]	2021	Middle school students	PA during PE (MVPA)	One item – extent of sweating during PE	Life satisfaction	Student Life Satisfaction Survey (SLSS)	Internalizing problems	Korean Emotional Behavioral Problem scale	Females: indirect effect: β = 0.02, *p* < 0.05	β = -.11, *p* <.05	β = -.21, *p* <.05		Yes
	You [228]	2021	Middle school students	PA during PE (MVPA)	One item – extent of sweating during PE	Life satisfaction	Student Life Satisfaction Survey (SLSS)	Internalizing problems	Korean Emotional Behavioral Problem scale	Males: indirect effect: β = 0.02, *p* < 0.05	β = -.16, *p* < .05	β = -.20, *p* < .05	β = .10, *p* < .05	Yes
	You [228]	2021	Middle school students	PA during PE (MVPA)	One item – extent of sweating during PE	Life satisfaction	Student Life Satisfaction Survey (SLSS)	Externalising problems	Korean Emotional Behavioral Problem scale	Females: indirect effect: β = 0.02, *p* < 0.05	β = -.10, *p* < .05	β = -.22, *p* < .05		Yes
	You [228]	2021	Middle school students	PA during PE (MVPA)	One item – extent of sweating during PE	Life satisfaction	Student Life Satisfaction Survey (SLSS)	Externalising problems	Korean Emotional Behavioral Problem scale	Males: indirect effect: β = 0.02, *p* < 0.05	β = -.10, *p* <.05	β = -.18, *p* <.05	β = .10, *p* <.05	Yes
	Broman-Fulks [26]	2018	Adults	Physical activity	One item rating the frequency of exercise	Psychological symptoms	Brief Symptom Inventory-18 anxiety subscale	Anxiety sensitivity	AS Index-3	Indirect effect: β = -.19, *p* <.001, Direct effect: β = -.28, *p* <.001	B = -1.50, *p* <.001	B = .12, *p* <.001	B = -.28, *p* <.001	Yes
	Broman-Fulks [26]	2018	Adults	Physical activity	One item rating the frequency of exercise	Psychological symptoms	Brief Symptom Inventory-18 somatization subscale	Anxiety sensitivity	AS Index-3	Indirect effect: β = -.10, *p* <.001, direct effect β = -.41, *p* <.001	B = -1.50, p <.001	B = .07, p <.001	B = -.41, p <.001	Yes
	Broman-Fulks [26]	2018	Adults	Physical activity	One item rating the frequency of exercise	Psychological symptoms	Brief Symptom Inventory-18 depression subscale	Anxiety sensitivity	AS Index-3	Indirect effect: β = -.18, *p* <.001, Direct effect β = -.39, *p* <.001	B = -1.50, *p* <.001	B = .12, *p* <.001	B = -.39, *p* <.001	Yes
	Awick [8]	2017	Older adults	Physical activity (MVPA)	Actigraph accelerometer	Δ Life Satisfaction	SWLS	Δ Psychological distress	Perceived stress scale (PSS)	Indirect effect: *B* = .05, 90% CI = .005, .125, *p* <.05, Direct effect ns (*p* = .28)	B = -.10, *p* = .05	B = -.51, *p* = .001	ns	Yes
	Elavsky [58]	2009	Peri- and post-menopausal women	Physical activity	Average mins per week	Stress	4-item version of the Perceived Stress Scale	Depressive symptoms	One item - "Feeling depressed/having 'the blues"	CFI = 1.000, SRMR = .012, RMSEA = .000, direct path of PA on depressed mood: β = −.15, *p* <.05, direct path of depressed mood on perceived stress: β = .30, *p* <.05	*r* = .18, *p* < .05	*r* = .46, *p* < .01	*r* = -.15, *p* < .05	Yes
	Watt [210]	2022	Psychology university students	Physical activity	Self-report	Psychological distress	Kessler 10 (K10)	Behavioural activation	Behavioral Activation for Depression Scale – Short Form (BADS-SF).		β = .027, CI = .018, .037	β = -.564, CI = -.634, -.493	β = -.018, CI = -.026, -.009	Yes
	Watt [210]	2022	Psychology university students	Physical activity	Asked how often they undertook physical activity	Depression	Kessler 10 (K10)	Behavioural activation	Behavioral Activation for Depression Scale – Short Form (BADS-SF).		β = 0.03, t (393) = 5.86, *p* <0.01	β = -0.33, t (388) = -12.55, *p* <0.01		Yes
	Watt [210]	2022	Psychology university students	Physical activity	Asked how often they undertook physical activity	Anxiety	Kessler 10 (K10)	Behavioural activation	Behavioral Activation for Depression Scale – Short Form (BADS-SF).		β = 0.03, t (393) = 5.86, *p* <0.01	β = -0.15, t (388) = -7.70, *p* <0.01		Yes
	Zhou [243]	2023	College students	Physical activity	Physical Activity Rating Scale	Life Satisfaction	Life Satisfaction Scale	Psychological distress	Depression Anxiety Stress Scale	β = .03, CI = .01, .04	β = -.07, *p* >.05	β = -.25, *p* <.05	β = -.01, *p* >.05	Yes
	Latimer [120]	2004	Individuals with traumatic spinal cord injury	Exercise	Intervention vs control	Depression	CES-D	Stress	14-item Perceived Stress Scale (PSS)	*F* (4,17) = 7.68, *p* < 0.01.	β = .50, t = 3.03, *p* <.01	β = .47, *t* = 3.01, *p* <.05	β = .70, *t* = 3.50, *p* <.05	Yes
	Zhang, X. [234]	2022	Tik tok users	Physical activity	2 items	Mental health issues (depression & anxiety)	Chinese version of the Patient Health Questionnaire (PHQ-9) + Chinese version of Generalized Anxiety Disorder (GAD-7)	Stress	Chinese version of the Perceived Stress Scale (PSS-10)	Indirect effect β= −0.079 (95% CI = −0.131, −0.032), p <.001	β = -.12, *p* <.01	β = .67, *p* <.001		Yes
	Yang [224]	2022	College students	Physical activity	Physical Activity Rating Scale (PARS-3)	Depressive symptoms	Patient Health Questionnaire	Perceived stress	Perceived Stress Scale (PSS-10)	Indirect effect = -.058 (-.086, -.032), effect size 20.2%	β = -.185, *p* <.001	β = .211, *p* <.001	β = -.198, *p* <.001	Yes

Pacewicz [167]	2022	Athletic trainers	Walking	IPAQ	Life satisfaction	Satisfaction with Life Scale	Stress and then depersonalisation	Perceived stress scale (PSS) + Maslach Burnout Inventory	Indirect path = -.02, p >.07				No
Pacewicz [167]	2022	Athletic trainers	Walking	IPAQ	Life satisfaction	Satisfaction with Life Scale	Stress and then exhaustion	Perceived stress scale (PSS) + Maslach Burnout Inventory	Indirect path =.09, p <.05				Yes
Pacewicz [167]	2022	Athletic trainers	Walking	IPAQ	Life satisfaction	Satisfaction with Life Scale	Stress and then personal accomplishment	Perceived stress scale (PSS) + Maslach Burnout Inventory	Indirect path = .04, p >.06				no
Wang [207]	2020	Middle school students	Sport Participation	Extracurricular sports subscale of the Middle School Student School Sports Assessment Scale	Life satisfaction	The Satisfaction with Life Scale for Chinese Adolescents	Academic stress	Academic stress subscale of the Middle School Student Psychological Health Questionnaire	Indirect effect = .10, SE = .01, 95% CI = .079, .124	*r* = -.29, *p* <.001	*r* = -.48, *p* < .001	*r* = .37, *p* < .001	Yes
Maher [146]	2015	Adults	Physical activity	Modified version of the GLTEQ	Life satisfaction	One item from the Satisfaction with Life Scale	Mental health	One item - "Today, my mental health was . . .,					Yes
Levante [124]	2024	University students	Physical activity	In the last months, how often have you done physical activity?	Psychological distress	DASS-21	Self-Reported Psychological Health Status	WHOQoL-Bref questionnaire	β = 0.130; Boot LLCI = 0.190; Boot ULCI = 0.072				Yes
Paxton [170]	2010	Older adults	Physical activity	Modified version of the GLTEQ	Life satisfaction	SWLS and the World Health Organization Well-Being Index (WHO-5)	Mental health difficulties	K-10		β = -.25, *p* <.05	β = -.36, *p* < .05	β = -.28, ns	Yes
Pickett [175]	2012	Adults with depression or low mood	Leisure time PA	Leisure-Time Exercise Questionnaire (LTEQ)	Depression	BDI-II	Affect	PANAS	Total indirect effect: B = -.74, 95% CI = -1.18, -.32				Yes
Elavsky (b) [57]	2005	Sedentary older adults	Physical activity	Physical Activity Survey for the Elderly (PASE)	Life Satisfaction	SWLS	Affect	Memorial University of New Foundland Scale of Happiness (MUNSH)	PA to affect β = .18, *p* < .05, affect to life satisfaction β = .59, *p* <.05, PA to life satisfaction direct effect = ns				Yes
Joseph [107]	2013	Undergraduate students	Physical activity	GLTEQ	Life satisfaction	SWLS	Positive affect	10-item Bradburn Affect Balance Scale	β = .27, *p* <.001	β =.10, *p* < .01)	β =0.27, *p* < .001		Yes
Kratz [115]	2014	Multiple sclerosis patients	Physical activity	7-Day Physical Activity Recall Interview (PAR)	Depressive symptoms	HAM-D	Positive affect	PANAS	PA to positive affect: β = .31, *p* <.01, Positive affect to depression: β = -.53, *p* <.01, PA to depression: β =.06, *p* >.10				Yes
Herzog [93]	2022	People with moderate or high anxiety	Physical activity	Physical Activity, Exercise, and Sport Questionnaire (BSA-F)	Anxiety	Anxiety Sensitivity Index 3	Positive affect	PANAS	a x b = 2.65, z = 3,76, p < .001				Yes
Joseph [107]	2013	Undergraduate students	Physical activity	GLTEQ	Life satisfaction	SWLS	Negative affect	10-item Bradburn Affect Balance Scale	β = .14, *p* <.001	β =.08, *p* < .01	β =0.27, *p* < .001		Yes
Kratz [115]	2014	Multiple sclerosis patients	Physical activity	7-Day Physical Activity Recall Interview (PAR)	Depressive symptoms	HAM-D	Negative affect	PANAS	PA to negative affect: β = -.16, *p* >.10, negative affect to depression: β = .38, *p* <.01, PA to depression: β =.06, *p* >.10				No
Hogan [96]	2015	Adults	Physical activity	IPAQ	Mental health	SF-36 MCS	Positive emotions	Modified Differential Emotions Scale	Cross sectional: indirect point estimate = .015 95% CI = .01, .02 Longitudinal: indirect point estimate = .058 95% CI = .01, .13	*r* = .33, *p* <.01	*r* = .44, *p* <.01	*r* = .24, *p* <.01	Yes
Hogan [96]	2015	Adults	Physical activity	IPAQ	Mental health	SF-36 MCS	Negative emotions	Modified Differential Emotions Scale	Cross sectional: indirect point estimate = .003 95% CI = .001, .004 Longitudinal: indirect point estimate = .009 95% CI = -.01, .06	*r* = -.10, ns	*r* = -.20, *p* <.01	*r* = .24, *p* <.01	Yes
Watt [210]	2022	Psychology university students	Physical activity	Asked how often they undertook physical activity	Psychological distress	Kessler 10 (K10)	Flow states	Short Dispositional Flow Scale (SDFS)		β = .018, CI = .012, .024	β = -.413, CI = -.554, -.271	β = -.018, CI = -.026, -.009	Yes

	Watt [210]	2022	Psychology university students	Physical activity	Asked how often they undertook physical activity	Depression	Kessler 10 (K10)	Flow states	Short Dispositional Flow Scale (SDFS)		ns	ns		No
	Watt [210]	2022	Psychology university students	Physical activity	Asked how often they undertook physical activity	Anxiety	Kessler 10 (K10)	Flow states	Short Dispositional Flow Scale (SDFS)		ns	ns		No
	Herzog [93]	2022	People with moderate or high anxiety	Physical activity	Physical Activity, Exercise, and Sport Questionnaire (BSA-F)	anxiety	Anxiety Sensitivity Index 3	Subjective vitality	Subjective Vitality Scale	a x b = 2.16, *z* = 3.38, *p* = .001				Yes
	Tian [199]	2022	College students	Physical activity	IPAQ-SF	Anxiety	Generalized Anxiety Disorder Scale (GAD-7)	Subjective wellbeing	General Well-Being Schedule (GWB)		β = .12, *p* < .01	β = -.71, *p* < .01	β = .0045, *p* > .05	Yes
	Tian [199]	2022	College students	Physical activity	IPAQ-SF	Anxiety	Generalized Anxiety Disorder Scale (GAD-7)	Subjective wellbeing	General Well-Being Schedule (GWB)					Yes
	Zhuo [245]	2023	College students	Physical activity	Physical Activity Rating Scale	Depression	Short-version Depression Scale compiled by Andresen et al. (1994)	Subjective wellbeing	Revised Subjective Well-Being Scale	Indirect effect = -.03, 95% CI = -.05, -.02	-.14, *p* <.01	.20, *p* <.01	-.03, *p* <.01	Yes
	Oshimi [166]	2022	Adults	MPA		Life satisfaction	Satisfaction with Life Scale (SWLS;	PERMA wellbeing	Mirehie and Gibson's (2020) 15-item scale	Indirect effect β = .10, CI = .07, .14				Yes
	Oshimi [166]	2022	Adults	VPA		Life satisfaction	Satisfaction with Life Scale (SWLS;	PERMA wellbeing	Mirehie and Gibson's (2020) 15-item scale	Indirect effect β = .01, CI = -.03, .04				No
	Görgülü [79]	2021	Patients with major depressive disorder	MVPA	Accelerometer	Depression	Beck-Depression-Inventory-II (BDI-II)	Habitual wellbeing	general habitual well-being using the FAHW questionnaire	Indirect effect: ab=− 0.43, 95% confidence interval: b = − 0.67 to b = − 0.14				Yes
	Tao [197]	2022	Young people	Physical activity	One self-report item	Subjective wellbeing	One self-report item	Life satisfaction	One self-report item	mediation effect = 0.0391, CI = .0073, .0711, 34.1%				Yes
	Li [127]	2023	High School students	Physical activity	"Do you participate in physical activity regularly?"	Psychological distress	Symptom Check-List-90	Life satisfaction	One item from the Child and Adolescent Quality of Life Scale	Indirect effect = −0.050 (low frequency of PA), −0.089 (medium frequency of PA), −0.100 (high frequency of PA)				Yes
	Liu, X. [143]	2024	Middle-aged and elderly women	Square dancing	Physical Activity Rating Scale	Affect	PANAS	Life Satisfaction	Satisfaction With Life Scale	Indirect effect = .003, 95% CI = -.002, .007	β = .023, *p* >.05	β = .201, *p* <.001	β = .080, *p* <.001	No
**Global self-esteem, self-worth, body image, appearance**	Ryan [182]	2008	Undergraduate students	Aerobic activity	Six-item Physical Activity subscale of the PSDQ	Depression	items from the SCL90R Depression subscale (SCL90R-D)	Self esteem	Physical Self-Description Questionnaire (PSDQ	PA to perceived endurance: β = .43, *p* < .001 (males), β = .53, *p* < .001 (females), perceived endurance to physical esteem: β = .60, *p* = <.001 (males), β = .54 *p* < .001, physical esteem to global esteem: β = .64, *p* = <.001 (males), β = .63 *p* < .001 (females), global esteem to depression: β = -.51, *p* = <.001 (males), β = -.11 ns (females), direct path: β = .03, ns (males) β = .00, ns (females).				Yes
	McPhie [152]	2012	Adolescents	Physical activity	Three items assessed the frequency of participation in activities during the past 7 days	Depressive symptoms	19-item version of the CES-D	Self esteem	Items modified from, or similar to items in, the Rosenberg Self-Esteem Inventory	Early adolescents: Boys - Sobel *z* = 8.05, *p* < .001, girls ns. Late adolescents: Boys - Sobel *z* = 8.33, *p* < .001, Girls Sobel *z* = 5.88, *p* < .001				Yes
	Wut [221]	2023	working adults	Moderate physical activity	IPAQ-SF	Depression	CES-D 20	Self-esteem			β = .195, *p* = .047	β = -.320, *p* = .006		Yes
	Wut [221]	2023	working adults	Moderate physical activity	IPAQ-SF	Anxiety	GAD-7	Self-esteem			β = .195, *p* = .047	β = -.298, *p* = .041		Yes
	Wut [221]	2023	working adults	Moderate physical activity	IPAQ-SF	Mental wellbeing	Warwick–Edinburgh Mental Well-being Scale	Self-esteem			β = .195, *p* = .047	β = .746, *p* = <.001		Yes
	Shang [186]	2021	College students	Physical activity	Physical activity rating scale (PARS-3)	Subjective wellbeing	General well-being schedule	Self esteem	Self-esteem scale (SES) compiled by Rosenberg	Single mediating path via body image [indirect effect = 0.011, 95% CI: (-0.003, 0.028)]				Yes
	Chen [36]	2022	Older adults	Physical activity	Physical Activity Rating Scale (PARS-3)	Subjective wellbeing	Subjective Well-Being Scale (SWB)	Self esteem	Rosenberg Self-Esteem Scale (SES)	Indirect effect β = .0692, CI = .0374, .1071	.056, *p* <.001	.108, *p* < .001	.031, *p* < .001	Yes

Görgülü [79]	2021	Patients with major depressive disorder	MVPA	Actigraph	Depression	Beck-Depression-Inventory-II (BDI-II)	Self esteem	Multidimensional Self-Value Scale (MSWS)	Indirect effect ab = − 0.22, 95% confidence interval b = − 0.41 to b = − 0.04				Yes
Zhang, J. [238]	2022	College students	Physical activity	Physical Activity Scale	Depression	Self-Rating Depression Scale	Self-concept	Self-concept scale	β = -.103, CI = -.18, -.05				Yes
Buffart [27]	2014	Cancer survivors	Exercise intervention	Intervention vs control	Depression and anxiety	14-item HADS	Self-efficacy	General self-efficacy scale	Indirect effect: β = -.04, p <.05	β = .13, p < .05	β - .30, p < .05	β -.09, p = .05	Yes
Barr [14]	2010	Physically active men	Aerobic exercise	Intervention vs control	Affect	Activation Deactivation Adjective Checklist	Self-efficacy	Eight items assessing belief in ability for the task	β = -.52, p < .05				Yes
Ho [94]	2015	School students	Physical activity	Physical Activity Rating Questionnaire for Children and Youth (PARCY)	Mental health	SF-36 MCS	Self-efficacy	General Self-Efficacy Scale	b = 0.19, p < .001, mediation proportion = 36%	B = .41, p < .001	B = 0.10, ns	B = .16, ns	Yes
Wang [208]	2022	College students	Physical activity	Physical Activity Rating Scale (PARS-3)	Subjective Well-Being	Subjective Well-Being Scale (SWS)	Self-efficacy	General Self-Efficacy Scale (GSES)	ES = 0.057				Yes
Xiong [223]	2023	Older adults	Physical activity	The Physical Activity Rating Scale (PARS-3)	Depression	Center for Streaming Depression Scale (CES-D)	Self-efficacy	Self-Efficacy Scale (GSES)	β = −0.101, 95%CI −0.149, −0.058				Yes
Kong [112]	2022	Older adults	Physical activity	Self-report	Depressive symptoms	K-GDS-SF	Self-efficacy	Chinese version of the general self-efficacy scale, developed by Zhang and Schwarzer	B = 0.3407, 95%CI = 0.1190, 0.5625				Yes
Zhao [240]	2021	College students	Physical activity	PARS-3	Psychological distress	Symptom self-rating scale	Self-efficacy	General self-efficacy scale					Yes
Baker [11]	2023	Women	Leisure-time physical activity	IPAQ	Depressive symptoms	CES-D 11	Self-efficacy	adapted from other measures	β = -0.018 (-0.033, -0.003)				Yes
Baker [11]	2023	Women	Transport physical activity	IPAQ	Depressive symptoms	CES-D 20	Self-efficacy	adapted from other measures	β = -0.011 (-0.020, -0.004)				Yes
Baker [11]	2023	Women	Occupational physical activity	IPAQ	Depressive symptoms	CES-D 29	Self-efficacy	adapted from other measures	β = -0.008 (-0.016, -0.002)				Yes
Baker [11]	2023	Women	Domestic physical activity	IPAQ	Depressive symptoms	CES-D 38	Self-efficacy	adapted from other measures	β = -0.004 (-0.009, -0.0002)				Yes
Dong [50]	2023	Adolescents	Physical activity	Physical Activity Questionnaire for Children (PAQ-C)	Mental health	Mental Health Inventory of Middle School Students (MMHI-60)	Self-efficacy	General self-efficacy scale	−0.198 × (-0.258) =−0.059				No
Li, B. [129]	2024	College students	Physical activity	IPAQ-SF	Depression	CES-D	Self-efficacy	General Perceived Self-efficacy Scale	Total effect of this indirect pathway was 0.065				Yes
Guo [84]	2024	College students	Physical activity	Physical Activity Scale	Life satisfaction	SWLS + PANAS	Self-efficacy	Self-Efficacy Scale	Indirect effect = 0.091, 95% CI = 0.067, 0.122	β = .358, p <.01	β = .3255, p <.01	β = .369, p <.01	Yes
Castan [28]	2024	Adults with spinal cord injury	Leisure-time PA	Physical Activity Recall Assessment for People with SCI (PARA-SCI)	Psychological distress	Hospital Anxiety and Depression Scale (HADS-D)	Self-efficacy	General Self-Efficacy Scale		β = .25, p <.05	β = -.52, p <.05	β = .037, p >.05	Yes
Phillips [174]	2013	Community-dwelling adults	Physical activity	Physical Activity Scale for the Elderly (PASE)	Life satisfaction	5-item Satisfaction with Life Scale	Self-efficacy and then physical self-worth	6-item Exercise Self-efficacy Scale, 6-item Physical Self-Worth Scale (PSW) of the physical self-perception profile (PSPP)	Indirect effect: p <.05				Yes
Phillips [174]	2013	Community-dwelling adults	Physical activity	Physical Activity Scale for the Elderly (PASE)	Life satisfaction	5-item Satisfaction with Life Scale	Self-efficacy and then via disability limitations	6-item Exercise Self-efficacy Scale, eight-item disability limitations subscale of the abbreviated Late-Life Function and Disability Instrument (LL-FDI)	Indirect effect: ns				Yes
Joseph [107]	2013	Undergraduate students	Physical activity	GLTEQ	Life satisfaction	SWLS	Physical self-esteem	6-item Physical Self-Worth Scale (PSWS)	β = .30, p <.001	β =.10, p < .001	β =0.30, p < .001		Yes
Elavsky (a) [56]	2005	Women	Physical activity	Aerobics Center Longitudinal Study Physical Activity Survey (ACLS)	Life Satisfaction	SWLS	Physical self-worth	Physical Self-Description Questionnaire (PSDQ)	PA to PSW β = .28, p < 0.05, PSW to SWL β = .48, p < 0.05	r = .40, p < .001	r = .55, p < .001	r = .20, p < .05	Yes

Aguinaga [2]	2018	Low-active older adults	Leisure-time PA	GLTEQ	Depression	HADS	Physical Self-Worth.	Subscale from the Physical Self-Perception Profile	Indirect effect: β = −.05; 95% confidence interval = -.11, -.01, $p <.05$, direct effect β = 0.13, $p = .02$	$r = .15$, $p = .02$	$r = -.36$, $p = .001$	$r = -.19$, $p = .003$	Yes
Aguinaga [2]	2018	Low-active older adults	Leisure-time PA	GLTEQ	Anxiety	HADS	Physical Self-Worth.	Subscale from the Physical Self-Perception Profile	Indirect effect: β = −.04; 95% confidence interval = −.09, -.01, $p <.05$, direct effect ns	$r = .15$, $p = .02$	$r = -.30$, $p = .001$	$r = -.14$, $p = .03$	Yes
Elavsky (b) [57]	2005	Sedentary older adults	Physical activity	Physical Activity Survey for the Elderly (PASE)	Life Satisfaction	SWLS	Physical self-worth	The Physical Self-Worth scale (PSW) of the Physical Self-Perception Profile	PA to physical self-worth: β = .38, $p < .05$, physical self-worth to life satisfaction = ns, PA to life satisfaction direct effect = ns				No
Herring [92]	2021	Young adults	Physical activity	7-day PA recall	Generalised anxiety disorder	10-item GAD subscale of the Psychiatric Diagnostic Screening Questionnaire	Social Physique anxiety	12-item Social Physique Anxiety Scale (SPAS)	Indirect effect β = -.21, $p = .021$, direct effect β = -.14, $p = .338$				Yes
Herring [92]	2021	Young adults	Physical activity	7-day PA recall	Social anxiety disorder	15-item SAD subscale of the Psychiatric Diagnostic Screening Questionnaire	Social Physique anxiety	12-item Social Physique Anxiety Scale (SPAS)	Indirect effect: β = -.27, $p = .012$, direct effect: β = -.09, $p = .395$				Yes
Herring [92]	2021	Young adults	Physical activity	7-day PA recall	Panic disorder	8-item PD subscale of the Psychiatric Diagnostic Screening Questionnaire	Social Physique anxiety	12-item Social Physique Anxiety Scale (SPAS)	Indirect effect: β = -.11, $p = .170$, direct effect: β = -.46, $p = .024$				No
Greenleaf [80]	2009	Undergraduate students	School sport	High School Sport Participation questionnaire	Psychological wellbeing	5-item SWLS + 10-item Self-Esteem Scale (SES)	Body image	8-item Body factor from the Body Parts Satisfaction Scale-Revised (BPSS-R) + The 10-item Body Shape Questionnaire-Revised (BSQR-10)	Sport to body image: β = .06, $p > .05$. Not a direct mediator but significant in multiple mediation model.				Yes
Shang [186]	2021	College students	Physical activity	Physical activity rating scale (PARS-3)	Subjective wellbeing	general well-being schedule	Body image	Multidimensional body self-relations questionnaire (MBSRQ)	Single mediating path via self-esteem [indirect effect = 0.087, 95% CI: (0.037, 0.141)]				No
Jia [104]	2023	Obese primary school students	Physical activity	Liang Deqing physical activity rating Scale	Psychological distress	DASS-c21	Body image	Body image State Scale	Indirect effect = 0.10, 95% CI = 0.04, 0.16				Yes
Chae [30]	2016	High School students	Physical activity	Physical Activity Questionnaire - Adolescent	Depression and anxiety	Korean version of the Children's Depression Inventory	Body esteem - appearance	The Body-Esteem Scale for Adolescents and Adults	BOYS: total indirect effect through appearance, weight, and attribution: β = -.07, $p < .05$, GIRLS: total indirect effect through appearance, weight, and attribution: β = -.11, $p < .05$	β = .17, $p <.01$ (girls) β = .11, $p <.05$ (boys)	β = -.49, $p <.05$ (girls) β = -.44, $p <.05$ (boys)		Yes
Chae [30]	2016	High School students	Physical activity	Physical Activity Questionnaire - Adolescent	Depression and anxiety	Korean version of the Children's Depression Inventory	Body esteem - weight	The Body-Esteem Scale for Adolescents and Adults	BOYS: total indirect effect through appearance, weight, and attribution: β = -.07, $p < .05$, GIRLS: total indirect effect through appearance, weight, and attribution: β = -.11, $p < .05$	β = .01, $p >.05$ (girls) β = .13, $p <.05$ (boys)	β = -.02, $p >.05$ (girls) β = -.03, $p > .05$ (boys)		Yes
Chae [30]	2016	High School students	Physical activity	Physical Activity Questionnaire - Adolescent	Depression and anxiety	Korean version of the Children's Depression Inventory	Body esteem - attribution	The Body-Esteem Scale for Adolescents and Adults	BOYS: total indirect effect through appearance, weight, and attribution: β = -.07, $p < .05$, GIRLS: total indirect effect through appearance, weight, and attribution: β = -.11, $p < .05$	β = .21, $p < .01$ (girls) β = .23, $p <.05$ (boys)	β = -.11, $p < .01$ (girls) β = -.10, $p > .05$ (boys)		Yes
Feuerhahn [64]	2012	Adults	Exercise after work	Self-reported duration of exercise that evening	Positive affect (in the evening)	6 items from the PANAS positive affect scale	Body attractiveness	Physical appearance subscale from the Physical Self-Description Questionnaire (PSDQ) German measure	$z = 3.37$, $p < .01$	$r = .18$, $p < .01$	$r = .09$, $p < .05$	$r = .22$, $p < .01$	Yes
Tihanyi [201]	2015	Advanced yoga participants	Yoga	Self-reported yoga frequency	Psychological wellbeing	WHO-5	Body awareness	Body Awareness Questionnaire	Indirect effect: β = .18, $p < .05$	$r = .24$, $p < .001$	$r = .39$, $p < .001$	$r = .26$, $p < .001$	Yes
Tihanyi [201]	2015	Advanced yoga participants	Yoga	Self-reported yoga frequency	Psychological wellbeing	WHO-5	Body image dissatisfaction	Body Image Ideals Questionnaire	Indirect effect: β = .20, $p < .01$	$r = -.21$, $p < .01$	$r = -.34$, $p < .001$	$r = .26$, $p < .001$	Yes

	Condello [41]	2016	Adults	Physical activity	American Alliance for Health, Physical Education, Recreation, and Dance exercise/medical history questionnaire	Mental health	SF-12 MCS	Body dissatisfaction	Body dissatisfaction index	Indirect effects:<br>PA to BMI = 3.50, p < .01,<br>BMI to BDI = .97, p <.01,<br>BDI to MCS = .20, p = .03,<br>Direct effect = 4.97, p = .04				Yes
	Zhang [233]	2021	Female cancer survivors	Physical activity	Derived from the WHI Physical Activity Questionnaire	Depressive symptoms	Yale Depression Screener	Body image concerns	Likert scale self-report 3 items	RR = 0.934, CI = 0.904, 0.966, mediated 7%				Yes
	Zhang [233]	2021	Female cancer survivors	Physical activity	Derived from the WHI Physical Activity Questionnaire	Anxiety	Self-report Likert scale items	Body image concerns	Likert scale self-report 3 items	RR = 0.929, CI = 0.879, 0.982, mediated 8.8%				Yes
	Zhang [233]	2021	Female cancer survivors	Physical activity	Derived from the WHI Physical Activity Questionnaire	Psychological distress	Self-report Likert scale items	Body image concerns	Likert scale self-report 3 items	RR = 0.934, CI = 0.881, 0.990, mediated 14.5%				Yes
	Gomez-Baya [77]	2017	Adolescents	Sport participation	One item – 'How often do you practice some sports or gymnastics during out-of-school time?'	Depressive symptoms	Children's Depression Inventory	Body satisfaction	One item – 'Do you like your physical appearance?'	Indirect effect: k2 = .08,<br>SE = .01, 95% CI = .06, .10, indirect effect was moderated by gender, as indicated by the index of moderated mediation, B = .04, SE = .02, 95% CI = −.01, .08				Yes
	Chu [39]	2023	Breast cancer survivors	Walking	IPAQ-L	Depression	Chinese version of the Hospital Anxiety and Depression Scale (HADS)	Body image satisfaction	body image subscale of the EORTC Quality of Life Questionnaire-breast cancer module (QLQ-BR23)	Standardized estimate = −0.07, 95% CI = −0.11 to −0.04 (indirect effect)	β = .27, p <.001	β = -.28, p <.001	β = -.14, p <.01	Yes
	Chu [39]	2023	Breast cancer survivors	Walking	IPAQ-L	Anxiety	Chinese version of the Hospital Anxiety and Depression Scale (HADS)	Body image satisfaction	body image subscale of the EORTC Quality of Life Questionnaire-breast cancer module (QLQ-BR23)	Standardized estimate = −0.07, 95% CI = −0.12 to −0.04 (indirect effect)	β = .27, p <.001	β = -.28, p <.001	β = -.11, p >.05	Yes
	Shang [186]	2021	College students	Physical activity	physical activity rating scale (PARS-3)	Subjective wellbeing	general well-being schedule	Body image and self esteem	Multidimensional body self-relations questionnaire (MBSRQ) + self-esteem scale (SES) compiled by Rosenberg	Serial mediating path via body image and self-esteem [indirect Effect = 0.038, 95% CI = 0.021, 0.158				Yes
	Riddervold [179]	2023	Late adolescents	Sport Participation	How often do you usually participate in these types of organised activities in your spare time?'	Life satisfaction	Huebner's Students' Life Satisfaction Scale	Body appreciation	Body Appreciation Scale 2	β = 0.120, 95% CI = 0.017, 0.147	β = .224, p = .001	β = .533, p = <.001	B = .026, p = .609	Yes
**Esteem and confidence related constructs but in relation to physical activity**	Ryan [182]	2008	Undergraduate students	Aerobic activity	Six-item Physical Activity subscale of the PSDQ	Depression	Items from the SCL90R Depression subscale (SCL90R-D)	Task efficacy	Developed by the author	PA to Task efficacy: β = .55, p < .001 (males), β = .51, p < .001 (females),<br>task efficacy to Depression β = .31, p < .05 (males), β = .36, p < .001 (females),<br>direct path from PA to depression: β = .03, ns (males) β = .00, ns (females)				Yes
	Goldstein [76]	2020	Adults	Exercise	Intervention vs control	Stress	10-item Perceived Stress Scale (PSS)	Exercise self-efficacy	Exercise Self-Efficacy Scale (ESES)	Indirect effect: β = -.01, p = .464,<br>direct effect β = -.12, p = .004				No
	Theodoropoulou [198]	2017	Adults	Physical activity	IPAQ-SF	Mental health	SF-36 MCS	Exercise self-efficacy	Self-efficacy scale	χ^2 = 517.029, df = 479, p = 0.001, χ^2/df = 1.079, CFI = 0.958, GFI = 0.924, IFI = 0.958, TLI = 0.953, RMSEA = 0.037	β = .34, p < .01	β = .19, p < .01	β = .07, p >.05	Yes
	Chair [31]	2020	Cardiac event or coronary heart disease patients	Leisure-time PA	Godin–Shephard Leisure-Time Physical Activity Questionnaire (GSLTPAQ)	Depressive symptoms	Chinese version of HADS (HADS-C)	Physical activity self-efficacy	Self-Efficacy for Exercise (SEE-C) Cantonese version	Indirect effect: β = −0.044, 95% CI: −.064, −.027,<br>direct effect: β = .003 (-.038, .044), p = .894				Yes
	Konopack [113]	2012	Adults	Physical activity	Actigraph accelerometer	Mental health	SF-36 MCS	Physical activity self-efficacy	Lifestyle Physical Activity Self-Efficacy Scale	PA to self-efficacy: β = .34,<br>self-efficacy to MH: β = .10				No

Guicciardi [82]	2019	Adults with multiple sclerosis	Leisure-time PA	GLTEQ	Mental health	SF-36 MCS	Exercise self-efficacy	Exercise Self-Efficacy	PA to exercise self-efficacy: β = .46, exercise self-efficacy to mental health: ns				No
Elavsky (b) [57]	2005	Sedentary older adults	Physical activity	Physical Activity Survey for the Elderly (PASE)	Life Satisfaction	SWLS	Exercise self-efficacy	Exercise Self-Efficacy scale	PA to self-efficacy β = .29, p < .05, self-efficacy to life satisfaction β = .17, p <.05, PA to life satisfaction direct effect = not significant				Yes
Joseph [107]	2013	Undergraduate students	Physical activity	GLTEQ	Life satisfaction	SWLS	Exercise self-efficacy	Exercise Self-Efficacy Scale		β =.28, p < .001	ns		No
Paxton [170]	2010	Older adults	Physical activity	Modified version of the GLTEQ	Life satisfaction	SWLS and the World Health Organization Well-Being Index (WHO-5)	Physical activity Self-efficacy	Modified version of the barrier self-efficacy scale and the self-efficacy for walking scale		β = .61, p <.05	β = .27, p <.05	β = -.28, ns	Yes
Baker [11]	2023	Women	Leisure-time physical activity	IPAQ	Depressive symptoms	CES-D 13	Behavioural intentions	Adapted from other measures	β = -0.012 (-0.024, -0.0002)				Yes
Baker [11]	2023	Women	Transport physical activity	IPAQ	Depressive symptoms	CES-D 22	Behavioural intentions	Adapted from other measures	β = -0.001 (-0.004, 0.001)				No
Baker [11]	2023	Women	Occupational physical activity	IPAQ	Depressive symptoms	CES-D 31	Behavioural intentions	Adapted from other measures	β = 0.002 (-0.001, 0.007)				No
Baker [11]	2023	Women	Domestic physical activity	IPAQ	Depressive symptoms	CES-D 40	Behavioural intentions	Adapted from other measures	β = -0.001 (-0.004, 0.001				No
Baker [11]	2023	Women	Leisure-time physical activity	IPAQ	Depressive symptoms	CES-D 14	Behavioural skills	Adapted from other measures	β = 0.005 (-0.007, 0.016)				No
Baker [11]	2023	Women	Transport physical activity	IPAQ	Depressive symptoms	CES-D 23	Behavioural skills	Adapted from other measures	β = 0.001 (-0.001, 0.004)				No
Baker [11]	2023	Women	Occupational physical activity	IPAQ	Depressive symptoms	CES-D 32	Behavioural skills	Adapted from other measures	β = -0.002 (-0.008, 0.003)				No
Baker [11]	2023	Women	Domestic physical activity	IPAQ	Depressive symptoms	CES-D 41	Behavioural skills	Adapted from other measures	β = -0.001 (-0.003, 0.001)				No
Greenleaf [80]	2009	Undergraduate students	School sport	High School Sport Participation questionnaire	Psychological wellbeing	5-item SWLS + 10-item Self-Esteem Scale (SES)	Physical competence	The Physical Self-Description Questionnaire (PDSQ)	Sport to competence: β = .60, p < .05, competence to body image: β = .25, p <.05, body image to wellbeing: β = .27, p <.05				Yes
Feuerhahn [64]	2012	Adults	Exercise after work	Self-reported duration of exercise that evening	Positive affect (in the evening)	6 items from the PANAS positive affect scale	Sports competence	Sports competence subscale from the Physical Self-Description Questionnaire (PSDQ)	z = 3.12, p < .01	r = .19, p < .01	r = .22, p < .01	r = .22, p < .01	Yes
Buffart [27]	2014	Cancer survivors	Exercise intervention	Intervention vs control	Depression and anxiety	14-item HADS	Mastery	Perlin Mastery Scale	Indirect effect: β = -.03, p <.05	β = .19, p < .05	β = -.19, p < .05	β -.09, p = .05	Yes
Deng [46]	2016	Young adults	Physical activity (MVPA)	Self-reported questionnaire	Depressive symptoms	20-item CES-D	Sense of mastery	Pearlin Mastery Scale	Indirect path through master (β = –.043, p < .001)	r = .14 (p value not reported)	r = -.35 (p value not reported)	r = -.07 (p value not reported)	Yes
Stuntz [192]	2020	Athletes	Exercise Intensity	Ratings of Perceived Exertion (RPE) scale	Positive affect	Feeling Scale	Accomplishment	One item – "How accomplished do you feel right now	Indirect effect: β = .19, p < .001, direct effect: β = -.14, p = .06	r = .61, p < .01	r = .26, p <. .01	r = -.05	Yes

**Personality**	Wilson [219]	2016	Fist year university students	Physical activity (MVPA)	Global Physical Activity Questionnaire (GPAQ), Godin Leisure Time Exercise Questionnaire (GLTEQ), and the International Physical Activity Questionnaire (IPAQ).	Mental health	SF-36 MCS	Neuroticism and Extraversion	International Personality Item Pool (IPIP)	CFI = .84, RMSEA = .07, SRMR = .12	Extraversion *r* =.22, *p* <.001, Neuroticism = -.11, *p* >.05	Extraversion *r* =.32, *p* <.001, Neuroticism = -.66, *p* <.001	*r* = .17, *p* <.05	No
	Wilson [219]	2016	Fist year university students	Physical activity (MVPA)	Actigraph accelerometer	Mental health	SF-36 MCS	Neuroticism and Extraversion	International Personality Item Pool (IPIP)	CFI = .86, RMSEA = .06, SRMR = .11	Extraversion *r* =.04, *p* >.05, Neuroticism *r* = -.27, *p* <.001	Extraversion *r* =.32, *p* <.001, Neuroticism *r* = -.66, *p* <.001	*r* = .22, *p* < .05	No
	Sato [185]	2016	Runners	Running	Participation in a running event	Life satisfaction	5-item SWLS	Pleasure	Nine involvement items of pleasure, centrality, and sign	Indirect path: β = .00 ns, direct path: β = .04 ns, indirect path through behavioural loyalty and then through pleasure: β = .01, *p* < .01				No
	Sato [185]	2016	Runners	Running	Participation in a running event	Life satisfaction	5-item SWLS	Centrality	Nine involvement items of pleasure, centrality, and sign	Indirect path β = -.00 ns, direct path β = .04 ns, indirect path through behavioural loyalty and then through pleasure: β = -.0011 ns				No
	Sato [185]	2016	Runners	Running	Participation in a running event	Life satisfaction	5-item Satisfaction with Life Scale	Sign	Nine involvement items of pleasure, centrality, and sign	Indirect path: β = .00 ns, direct path: β = .04 ns, indirect path through behavioural loyalty and then through pleasure: β = .01, *p* < .05				No
	González-Hernández [78]	2019	Adolescents	Physical activity	Global Physical Activity Questionnaire (GPAQv2)	Psychological Wellbeing	Psychological flourishing scale	Perfectionism	Multidimensional Perfection Scale	Vigorous PA indirect effect: β = .58; *p* < .01), Non-vigorous PA indirect effect: β = −.26; *p* < .001				Yes
	Greenleaf [80]	2009	Undergraduate students	School sport	High School Sport Participation questionnaire	Psychological wellbeing	SWLS + 10-item Self-Esteem Scale (SES)	Instrumentality	20-item Instrumentality Scale + 8-item Masculinity scale from the Personal Attributes Questionnaire—Short Form (PAQ)	Sport to instrumentality: β = .60, *p* < .05, instrumentality to body image: β = .25, *p* <.05, body image to wellbeing: β = .27, *p* <.05				Yes
	Kukihara [118]	2018	Older adults	Physical activity	No physical activity; physically active 3–4 days a week; or active 30-min every day	Depression	Geriatric Depression Scale Short Form	Morale	Philadelphia Geriatric Center Morale Scale	*z* = −2.34, *p* = .01	β = .13, *p* = .009	B = -.754, *p* = .0001	β = -.17, *p* = .001	Yes
	Kukihara [118]	2018	Older adults	Physical activity	No physical activity; physically active 3–4 days a week; or active 30-min every day	Mental health	The Medical Outcomes Short Form 8 (SF-8) mental health subscale	Morale	Philadelphia Geriatric Center Morale Scale	*z* = 2.32, *p* = .01	β = .13, *p* = .009	B = .612, *p* = .0001	β = .09, *p* = .043	Yes
	Cecchini [29]	2019	College students	Vigorous PA	IPAQ	Depressive symptoms	Depressive Symptoms Scale (DSS)	Approach-avoidance contrast temperament	Approach-Avoidance Temperament Questionnaire	Total effect through approach-avoidance contrast temperament β = -.015, *p* < .001, direct effect β = .05, *p* > .05	β = .31, *p* < .001	β = -.28, *p* < .001	β = -.15, *p* < .001	Yes
	Chen [36]	2022	Older adolescents and adults	Exercise	Self-designed exercise questionnaire	Psychological distress (Depression and Anxiety)	Generalized Anxiety Disorder Scale-7 and the Patient Health Questionnaire-9	Hope	Adult Dispositional Hope Scale	β = −0.046, 95% CI = −0.064, −0.027, *p* < .001				Yes
	Chen [36]	2022	Older adolescents and adults	Exercise	self-designed exercise questionnaire	Psychological distress (Depression and Anxiety)	Generalized Anxiety Disorder Scale-7 and the Patient Health Questionnaire-9	Hope	Adult Dispositional Hope Scale	β = −0.060, 95% CI = −0.081, −0.039, *p* < .001				Yes
	Zhang, X. [237]	2022	Adults	Exercise	Self-report	Subjective wellbeing	Brief Subjective Well-Being Scale for Chinese Citizens	Sense of hope	State Hope Scale	Indirect effect = 0.131, CI = 0.094, 0.185				Yes

Psychological factors related to physical activity

Jenkins [102]	2022	Adults	Nature-based PA	IPAQ-SF	Psychological wellbeing	Questionnaire for Eudaimonic Wellbeing (QWEB)	Motivation	BREQ-3		β = .29, *p* < .05	β = .13, *p* > .05	β = -.05, *p* > .05	Yes
Jenkins [102]	2022	Adults	Nature-based PA	IPAQ-SF	Hedonic Wellbeing	WHO-5	Motivation	BREQ-3		β = .29, *p* < .05	β = .09. *p* < .05	β = .04, *p* > .05	Yes
Jenkins [101]	2021	Adults	Weekly physical activity	IPAQ-SF	Psychological wellbeing	WHO-5	Intrinsic motivation	BREQ-3 PA	0.06 (0.01–0.12)	β = .43 *p* < .05	β = .14 *p* < .05	β = .17 *p* < .05	Yes
Smith [189]	2018	Adolescents	Resistance training	Intervention vs control	Subjective wellbeing	Psychological flourishing scale	Intrinsic motivation	Subscale from the Physical Self-Perception Profile	Indirect effect: β = .05, 95% CI = −.101, .190				No
Smith [189]	2018	Adolescents	Resistance training	Intervention vs control	Subjective wellbeing	Psychological flourishing scale	Identified regulation	Subscale from the Physical Self-Perception Profile	Indirect effect: β = -.02, 95% CI = −.183, .125				No
Jenkins [101]	2021	Adults	Weekly physical activity	IPAQ-SF	Psychological wellbeing	WHO-5	Introjected regulation	BREQ-3 PA	−0.03 (−0.05 to −0.01)	β = .18, *p* < .05	β = .17 *p* < .05	β = .17 *p* < .05	Yes
Kruk [116]	2019	Adults	Leisure time PA T1	GLTEQ	Depression T2	CES-D	PA liking T2	Liking of vigorous PA, liking sports, and PA enjoyment subscales derived from the Child Attraction to Physical Activity Scale (CAPA)	Indirect effect B = <.01, 95% CI = <-.01, <.01, *p* > .05, direct effect: B = <.01, 95% CI = <-.01, <.01, *p* > .05				No
Lin (b) [133]	2022	Undergrad students	Exercise	Self-report	Subjective wellbeing	Satisfaction with Life Scale (SWLS) + PANAS	Psychological need satisfaction	Psychological Need Satisfaction in Exercise Scale	Indirect effect = 0.17, 95% CI = 0.12, 0.23				Yes
Mack [145]	2013	Female breast cancer survivors	Leisure time PA	GLTEQ	Positive affect	PANAS	Autonomy	18-item Psychological Need Satisfaction in Exercise Scale (PNSE;	Indirect effect: β = .03, 95% CI = -.03, .12, ns	*r* = .07	*r* = .24**	*r* = .20*	No
Mack [145]	2013	Female breast cancer survivors	Leisure time PA	GLTEQ	Negative affect	PANAS	Autonomy	18-item Psychological Need Satisfaction in Exercise Scale (PNSE;	Indirect effect: β = .01, 95% CI = -.02, .09, ns	*r* = .07	*r* = −.14	*r* = −.18*	No
Mack [145]	2013	Female breast cancer survivors	Leisure time PA	GLTEQ	Stress	10-item Perceived Stress Scale (PSS)	Autonomy	18-item Psychological Need Satisfaction in Exercise Scale (PNSE;	Indirect effect: β = -.001, 95% CI = -.07, .05, ns	*r* = .07	*r* = −.22**	*r* = −.06	No
Mack [146]	2013	Female breast cancer survivors	Leisure time PA	GLTEQ	Depression	CES-D	Autonomy	18-item Psychological Need Satisfaction in Exercise Scale (PNSE;	Indirect effect: β = .01, 95% CI = -.02, .08, ns	*r* = .07	*r* = −.13	*r* = −.21**	No
Doré [52]	2020	Children	Leisure-time PA	Free-time engagement in PA via a 36-item PA and sport checklist.	Mental health	Mental Health Continuum-Short Form (MHC-SF)	Autonomy	7-item autonomy subscale of the Basic Psychological Needs in Life Scale	Indirect effect β = 1.06, *p* < .05, direct effect β = -.22, *p* > .05				Yes
O'Rourke [165]	2023	Adults with a disability	Leisure-time physical activity	Leisure-Time Physical Activity Questionnaire for People with Spinal Cord Injury (LTPAQ-SCI)	Mental health	Mental Health Continuum-Short Form (MHC-SF)	Autonomy	Basic Psychological Need Satisfaction Scale in General (BNSG-S)	β = 0.05, 95% CI = 0.00, 0.12	b = .001, *p* = .01	b = 2.93, *p* = .02	b = .019, *p* = .01	Yes
Mack [145]	2013	Female breast cancer survivors	Leisure time PA	GLTEQ	Depression	CES-D	Competence	18-item Psychological Need Satisfaction in Exercise Scale (PNSE;	Indirect effect: β = - .003, 95% CI = -.07, .02, ns	*r* = .21*	*r* = −.09	*r* = −.21**	No
Mack [145]	2013	Female breast cancer survivors	Leisure time PA	GLTEQ	Negative affect	PANAS	Competence	18-item Psychological Need Satisfaction in Exercise Scale (PNSE;	Indirect effect: β = -.02, 95% CI = -.11, .01, ns	*r* = .21*	*r* = −.07	*r* = −.18*	No
Mack [145]	2013	Female breast cancer survivors	Leisure time PA	GLTEQ	Positive affect	PANAS	Competence	18-item Psychological Need Satisfaction in Exercise Scale (PNSE;	Indirect effect β = .02, 95% CI = -.01, .10, ns	*r* = .21*	*r* = .23**	*r* = .20*	No
Mack [145]	2013	Female breast cancer survivors	Leisure time PA	GLTEQ	Stress	10-item Perceived Stress Scale (PSS)	Competence	18-item Psychological Need Satisfaction in Exercise Scale (PNSE;	Indirect effect: β = -.01, 95% CI = -.09, .02, ns	*r* = .21*	*r* = −.14	*r* = −.06	No
Doré [52]	2020	Children	Leisure-time PA	Free-time engagement in PA via a 36-item PA and sport checklist.	Mental health	Mental Health Continuum-Short Form (MHC-SF)	Competence	Intrinsic Motivation Inventory	Indirect effect: β = -.67, *p* > .05, direct effect: β = -.22, *p* > .05				Yes
O'Rourke [165]	2023	Adults with a disability	Leisure-time physical activity	Leisure-Time Physical Activity Questionnaire for People with Spinal Cord Injury (LTPAQ-SCI)	Mental health	Mental Health Continuum-Short Form (MHC-SF)	Competence	Basic Psychological Need Satisfaction Scale in General (BNSG-S)	β = 0.16, 95% CI = 0.08, 0.25	b = .002, *p* <.001	b = 5.40, *p* <.001	b = .019, *p* = .01	Yes

Mack [145]	2013	Female breast cancer survivors	Leisure time PA	GLTEQ	Negative affect	PANAS	Relatedness	18-item Psychological Need Satisfaction in Exercise Scale (PNSE;	Indirect effect: β = -.05, 95% CI = -.22, .02, ns	*r* = .13	*r* = −.24**	*r* = −.18*	No
Mack [145]	2013	Female breast cancer survivors	Leisure time PA	GLTEQ	Positive affect	PANAS	Relatedness	18-item Psychological Need Satisfaction in Exercise Scale (PNSE;	Indirect effect: β = .04, 95% CI = .01, .16, p <.01	*r* = .13	*r* = .30**	*r* = .20*	Yes
Mack [145]	2013	Female breast cancer survivors	Leisure time PA	GLTEQ	Stress	10-item Perceived Stress Scale (PSS)	Relatedness	18-item Psychological Need Satisfaction in Exercise Scale (PNSE;	Indirect effect: β = -.04, 95% CI = -.15, .01, ns	*r* = .13	*r* = −.24**	*r* = −.06	No
Mack [145]	2013	Female breast cancer survivors	Leisure time PA	GLTEQ	Depression	CES-D	Relatedness	18-item Psychological Need Satisfaction in Exercise Scale (PNSE;	Indirect effect: β = -.02, 95% CI = -.11, .01, ns	*r* =.13	*r* = −.20*	*r* = −.21**	No
Doré [52]	2020	Children	Leisure-time PA	Free-time engagement in PA via a 36-item PA and sport checklist.	Mental health	Mental Health Continuum-Short Form (MHC-SF)	Relatedness	Relatedness to Others in Physical Activity Scale	Indirect effect: β = 1.45, *p* < .05,<br>direct effect β = -.22, *p* > .05				Yes
O'Rourke [165]	2023	Adults with a disability	Leisure-time physical activity	Leisure-Time Physical Activity Questionnaire for People with Spinal Cord Injury (LTPAQ-SCI)	Mental health	Mental Health Continuum-Short Form (MHC-SF)	Relatedness	Basic Psychological Need Satisfaction Scale in General (BNSG-S)	β = 0.08, 95% CI = 0.02 to 0.17	b = .001, *p* = .02	b = 4.94, *p* <.001	b = .019, *p* = .01	Yes
Chen [35]	2019	Older adults	Physical activity	33 items that captured a wide range of activities (reading, walking, volunteering, visiting with friends, etc.)	Depressive symptoms	Modified version of the CES-D	Nature of engagement	Two questions asking how often respondents use their body and mind when they were doing activities	Full engagement mediated activity and depressive association when comparing moderate activity vs passive activity (95% CI = -.20, -.01).<br>Full engagement mediated activity and depressive association when comparing high activity vs passive activity (95% CI = -.56, -.04).<br>Full engagement did not mediate activity and depressive association when comparing low activity vs passive activity (95% CI = -.15, .001).				Yes
Ryan [182]	2008	Undergraduate students	Aerobic activity	Six-item Physical Activity subscale of the PSDQ	Depression	items from the SCL90R Depression subscale (SCL90R-D)	Scheduling efficacy	Developed by the author	PA to scheduling efficacy:<br>β = .54, *p* < .001 (males)<br>β = .52, *p* < .001 (females)<br>Scheduling efficacy to Depression: β = -.43, *p* = .015 (males) β = -.30, *p* = .02 (females), direct path from PA to depression β = .03, ns (males) β = .00, ns (females)				No
Nezlek [162]	2018	Regular runners	Running	Indicated if they had ran the previous day	Life satisfaction	three items based on the SWLS	Satisfaction with perceived progress	One item - “How satisfied were you with this progress?”	Indirect effect: β = .10, *p* <.001				Yes
Nezlek [162]	2018	Regular runners	Running	Indicated if they had ran the previous day	Positive activated affect	Based on the circumplex mode	Satisfaction with perceived progress	One item - “How satisfied were you with this progress?”	Indirect effect: β = .12, *p* <.001				Yes
Nezlek [162]	2018	Regular runners	Running	Indicated if they had ran the previous day	Positive deactivated affect	Based on the circumplex mode	Satisfaction with perceived progress	One item - “How satisfied were you with this progress?”	Indirect effect: β = .09, *p* <.001				Yes
Nezlek [162]	2018	Regular runners	Running	Indicated if they had ran the previous day	Negative activated affect	Based on the circumplex mode	Satisfaction with perceived progress	One item - “How satisfied were you with this progress?”	Indirect effect: β = -.07, *p* <.001				Yes
Nezlek [162]	2018	Regular runners	Running	Indicated if they had ran the previous day	Negative deactivated affect	Based on the circumplex mode	Satisfaction with perceived progress	One item - “How satisfied were you with this progress?”	Indirect effect: β = -.10, *p* <.001				Yes
Sato [185]	2016	Runners	Running	Participation in a running event	Life satisfaction	5-item Satisfaction with Life Scale	Behavioural loyalty	the average number of days and miles run per week during the last 3 months	Indirect path: β = .01 ns, direct path β = .04 ns				No
Zhang, Z. [235]	2022	College students	Physical activity	IPAQ-SF	Psychological distress (Depression and Anxiety)	Chinese version of Depression Anxiety Stress Scale (DASS-2	Exercise intensity-tolerance and preference	Chinese version (under review) of the Preference for and Tolerance of the Intensity of Exercise	Indirect effect = 0.006714, 95% CI = 0.000032, 0.000128				Yes

									Questionnaire (PRETIE-Q					
	Zeibig [231]	2023	Adults	Exercise	Group allocation to intervention vs control	Psychological distress	Symptom Checklist-90, SCL-90-R	exercise-specific affect regulation	Physical Activity-related Health Competence Questionnaire)	ß = 0.28, p = .037				Yes
	Baker [11]	2023	Women	Leisure-time physical activity	IPAQ	Depressive symptoms	CES-D 10	Enjoyment of PA	Adapted from other measures	β = -0.016 (-0.030, -0.001)				Yes
	Baker [11]	2023	Women	Transport physical activity	IPAQ	Depressive symptoms	CES-D 19	Enjoyment of PA	Adapted from other measures	β = -0.003 (-0.008, -0.0001)				No
	Baker [11]	2023	Women	Occupational physical activity	IPAQ	Depressive symptoms	CES-D 28	Enjoyment of PA	Adapted from other measures	β = 0.000 (-0.004, 0.005)				No
	Baker [11]	2023	Women	Domestic physical activity	IPAQ	Depressive symptoms	CES-D 37	Enjoyment of PA	Adapted from other measures	β = -0.001 (-0.004, 0.001)				No
**Other psychological factors**	Feuerhahn [64]	2012	Adults	Exercise after work	Self-reported duration of exercise that evening	Positive affect (in the evening)	6 items from the PANAS positive affect scale	Psychological detachment		z = 2.74, p < .01	r = .10, p < .01	r = .20, p < .01	r = .22, p < .01	Yes
	Ginoux [75]	2021	Adults (during Covid lockdown)	Physical activity (MVPA)	IPAQ	Wellbeing (i.e., vitality, stress, and psychological need satisfaction)	French versions of the of the Subjective Vitality Scale, four-item Perceived Stress Scale (PSS), and Basic Psychological Need Satisfaction and Frustration Scale (BPNSFS)	Recovery experience (i.e., psychological detachment, relaxation, mastery, control)	French version of the Recovery Experience Questionnaire	Indirect effect: β = .19, 95% CI = .01, .02, direct effect β = -.02, 95% CI = -.01, .01				Yes
	Marselle [149]	206	Regular walkers	Walking	Perceived intensity	Positive affect	PANAS	Perceived restorativeness	16-item Perceived Restorativeness Scale	ab = 0.11, p < .001, CI = .06, .16	B = .85, p < .05	B = .13, p < .05	B = .40, p < .001	Yes
	Marselle [149]	206	Regular walkers	Walking	Perceived intensity	Negative affect	PANAS	Perceived restorativeness	16-item Perceived Restorativeness Scale	ab = 0.01, p = .01, CI = .02, .00	B = .96, p <.05	B = -.01, p < .05	B = .04, ns	Yes
	Marselle [149]	2016	Regular walkers	Walking	Duration	Positive affect	PANAS	Perceived restorativeness	16-item Perceived Restorativeness Scale	ab = .12, p = .05, CI = .00, .25	B = .91, ns	B = .13, p < .05	B = .22, ns	No
	Marselle [149]	2016	Regular walkers	Walking	Duration	Negative affect	PANAS	Perceived restorativeness	16-item Perceived Restorativeness Scale		B = 1.13, p <.05	B = -.01, p < .05	B = -.20, ns	No
	Tihanyi [201]	2015	Advanced yoga participants	Yoga	Self-reported yoga frequency	Psychological wellbeing	WHO-5	Mindfulness	Mindful Attention and Awareness Scale	Indirect effect: β = .20, p < .01	r = .22, p < .01	r = .33, p < .001	r = .26, p < .001	No
	Goldstein [76]	2020	Adults	Exercise	Intervention vs control	Stress	10-item Perceived Stress Scale (PSS)	Mindful attention awareness	Mindful Attention Awareness Scale (MAAS)	Indirect effect β = -.02, p = .03, direct effect β = -.12, p = .004				Yes
	Goldstein [76]	2020	Adults	Exercise	Intervention vs control	Stress	10-item Perceived Stress Scale (PSS)	Mindfulness self-efficacy	Mindfulness-Based Self-Efficacy Scale - Revised (MSES-R)	Indirect effect β = -.06, p = .04, direct effect β = -.12, p = .004				Yes
	La Rocque [119]	2021	Women	Yoga	Intervention vs control	Depression	17-item HAM-D	Mindfulness acceptance	20-item Philadelphia Mindfulness Scale	Indirect effect: Coef = -1.67, 95% CI = -4.09, .11				No
	La Rocque [119]	2021	Women	Exercise	Intervention vs control	Depression	17-item HAM-D	Mindfulness acceptance	20-item Philadelphia Mindfulness Scale	Indirect effect: Coef = -2.67, 95% CI = -5.26, -.55				Yes
	La Rocque [119]	2021	Women	Yoga	Intervention vs control	Depression	17-item HAM-D	Rumination	Ruminative Responses Scale	Indirect effect: Coef = -3.02, 95% CI = -5.67, -.50				Yes
	La Rocque [119]	2021	Women	Exercise	Intervention vs control	Depression	17-item HAM-D	Rumination	Ruminative Responses Scale	Indirect effect: Coef = -4.43, 95% CI = -6.99, -1.83				Yes
	Kukihara [118]	2018	Older adults	Physical activity	No physical activity; physically active 3–4 days a week; or active 30-min every day	Depression	Geriatric Depression Scale Short Form	Sense of coherence	The SOC Scale	not reported	β = .05, p = .183	B = -4.994, p = .000	β = -.17, p = .001	No
	Kukihara [118]	2018	Older adults	Physical activity	No physical activity; physically active 3–4 days a week; or active 30-min physical activity every day	Mental health	The Medical Outcomes Short Form 8 (SF-8) mental health subscale	Sense of coherence	The SOC Scale	not reported	β = .05, p = .183	B = 0.427, p = .0001	β = .09, p = .043	No
	Chen [36]	2022	Older adults	Physical activity	Physical Activity Rating Scale (PARS-3)	Subjective wellbeing	e Subjective Well-Being Scale (SWB)	Sense of meaning in life	Meaningful Life Scale (MLQ)	indirect effect β = .0854, CI = 0.0474, 0.1331	.119, p<.001	.063, p<.001	.031, p<.001	Yes
	Chen [36]	2022	Older adults	Physical activity	Physical Activity Rating Scale (PARS-3)	Subjective wellbeing	e Subjective Well-Being Scale (SWB)	Sense of meaning in life + self esteem	Meaningful Life Scale (MLQ) + Rosenberg Self-Esteem Scale (SES)	indirect effect β = .0237, CI = .0116, .0396				Yes

Precht [176]	2021	University students	Physical activity	One item measure	Mental health	Positive mental health scale	Sense of control	Two items	Indirect effect = .42, 95%CI = .207, .628	β = -.28	β = -1.46	β = .89	Yes
Precht [176]	2021	University students	Physical activity	One item measure	Depression	DASS-21	Sense of control	Two items	Indirect effect = -.42, 95%CI = -.207, -.629				Yes
Precht [176]	2021	University students	Physical activity	One item measure	Anxiety	DASS-22	Sense of control	Two items	Indirect effect = -.30, 95%CI = -.457, -.154				Yes
Precht [176]	2021	University students	Physical activity	One item measure	Stress	DASS-23	Sense of control	Two items	Indirect effect = -.35, 95%CI = -.527, -.173				Yes
Zhou [243]	2023	College students	Physical activity	Physical Activity Rating Scale	Life Satisfaction	Life Satisfaction Scale	Self-control	self-control scale	β = .06, CI = .04, .10	β = .21, $p < .05$	β = .30, $p < .05$	β = -.01, $p > .05$	Yes
Kayani [109]	2021	University students	Physical activity	Self-report	Anxiety	State-Trait Anxiety Inventory (STAI Y-6)	Self-criticism	Level of SC scale (LOSC)	−0.0498 (−0.1018, −0.0099)	β = -.133	β = .372	β = -.176	Yes
Tang [196]	2022	College students	Physical activity	Physical activity rating scale (PARS-3)	Depression	The "Depression Self-Rating Scale (SDS)"	Emotional regulation self-efficacy	The "Emotional Regulation Self-Efficacy Scale (RES)"	Indirect effect = -.031, CI = -.072, -.001				Yes
Tang [196]	2022	College students	Physical activity	Physical activity rating scale (PARS-3)	Anxiety	The "Anxiety Scale (SAS-CR)"	Emotional regulation self-efficacy	The "Emotional Regulation Self-Efficacy Scale (RES)"	Indirect effect = -.104, CI = -.160, -.057				Yes
Wang [208]	2022	College students	Physical activity	Physical Activity Rating Scale (PARS-3)	Subjective Well-Being	Subjective Well-Being Scale (SWS)	Emotional intelligence	Emotional Intelligence Scale (EIS)	ES = 0.077				Yes
Fernandes [62]	2024	Adolescents	Sport Participation	Single-item question	Life satisfaction	Satisfaction with Life Scale	Emotional intelligence - self-emotional appraisal	Portuguese version of the Wong and Law Emotional Intelligence Scale	Indirect effect = .03, 95% CI = .01, .07				Yes
Fernandes [62]	2024	Adolescents	Sport Participation	Single-item question	Life satisfaction	Satisfaction with Life Scale	Emotional intelligence - use of emotion	Portuguese version of the Wong and Law Emotional Intelligence Scale	Indirect effect = .06, 95% CI = .02, .12				Yes
Kozielly [114]	2022	Adults	Physical activity	Stanford Brief Activity Survey (SBAS)	Psychological distress	21-item Depression, Anxiety, and Stress Scale (DASS21)	Stress appraisal	Stress Appraisal Measure	Effect = –0.28 (se = 0.13), CI.95 [–0.56, –0.06]				Yes
Kozielly [114]	2022	Adults	Physical activity	Stanford Brief Activity Survey (SBAS)	Psychological distress	21-item Depression, Anxiety, and Stress Scale (DASS21)	Stress appraisal	Stress Appraisal Measure	Effect = –0.16 (se = 0.11), CI.95 [–0.39, 0.04]				No
Kozielly [114]	2022	Adults	Physical activity	Stanford Brief Activity Survey (SBAS)	Psychological distress	21-item Depression, Anxiety, and Stress Scale (DASS21)	Stress appraisal	Stress Appraisal Measure	Effect = –0.11 (se = 0.10), CI.95 [–0.32, 0.07]				No
Ho [94]	2015	School students	Physical activity	Physical Activity Rating Questionnaire for Children and Youth (PARCY)	Mental health	SF-36 MCS	Resilience	Connor-Davidson Resilience Scale (CD-RISC)	Single mediation model: b = .35, $p < .001$, mediation proportion = 68% Model including self-efficacy, resilience, and social connectedness: indirect effect b = .31, $p < .001$, direct effect = 16, $p = .18$	B = 1.60, $p < .001$	B = .19, $p < .001$	B = .16, ns	Yes
Kukihara [118]	2018	Older adults	Physical activity	No physical activity; physically active 3–4 days a week; or active 30-min every day	Depression	Geriatric Depression Scale Short Form	Resilience	The Resilience Scale	$z = −3.10$, $p = .0001$	β = .18, $p = .001$	β = -.50, $p = .0001$	β = -.17, $p = .001$	Yes
Kukihara [118]	2018	Older adults	Physical activity	No physical activity; physically active 3–4 days a week; or active 30-min every day	Mental health	The Medical Outcomes Short Form 8 (SF-8) mental health subscale	Resilience	The Resilience Scale	$z = 2.63$, $p = .004$	β = .18, $p = .001$	β = .30, $p = .0001$	β = .09, $p = .043$	Yes
Yoshikawa [227]	2016	Employees	Exercise Frequency	One item – In the last six months, how often did you do relatively hard exercise for more than 20 min?	Depression	CES-D	Resilience	Resilience Scale	BCACI = −1.89 to −.094	B = 3.20, SE = 1.30, $p = .01$	B = −0.30, SE = .02, $p < .01$	B = −.17, SE = .72, $p = .81$	Yes
Zhang, Z. [235]	2022	College students	Physical activity	IPAQ-SF	Psychological distress (Depression and Anxiety)	Chinese version of Depression Anxiety Stress Scale (DASS-2	Resilience	Chinese version of Connor-Davidson resilience scale (CD-RISC)	Indirect effect = -.024934, 95% CI = -0.000406, -.000048				Yes
Liang [131]	2022	Children with ADHD	MVPA	Accelerometer	Psychological distress	Depression Anxiety Stress Scale 21 (DASS 21)	Resilience	Conner-Davidson Resilience Scale (CD-RISC- 25)	−0.006%, 95% confidence interval [CI] = [−0.060, −0.020]				Yes
Xin [222]	2023	Older adults	Exercise	Self-report	Anxiety	generalized anxiety disorder 7-item (GAD-7)	Psychological resilience	Conner–Davidson resilience scale (CD-RISC)					Yes

Jia [104]	2023	Obese primary school students	Physical activity	Liang Deqing physical activity rating Scale	Psychological distress	DASS-c21	Resilience	Connor-Davidson resilience Scale	indirect effect = 0.02, 95% CI = 0.01, 0.03				Yes
Liu, X. [143]	2024	Middle-aged and elderly women	Square dancing	Physical Activity Rating Scale	Affect	PANAS	Resilience	Connor-Davidson Resilience Scale	indirect effect = .029, 95% CI = .017, .042	β = .102, p <.001	β = .483, p <.001	β = .080, p <.001	Yes
Zheng [242]	2024		Physical activity	Physical Activity Readiness Scale	Anxiety	Self-Rating Anxiety Scale	Resilience	Connor–Davidson Resilience Scale	indirect effect = -.20, 95% CI = -.31, -.10	β = .39, p <.01	β = -.20, p <.01	β = -.37, p <.01	Yes
Liu, M. [142]	2024	Undergraduate students	Physical activity	PARS-3	Psychological distress	DASS-21	Resilience	Connor-Davidson resilience scale (CD-RISC)	indiect effect = −0.0324, 95% CI = −0.0503, −0.0173				Yes
Bae [10]	2024	Elementary school students	Physical activity	Self-report	Life satisfaction	Satisfaction Life Satisfaction Scale	Grit	Children's Grit Scale	B = 0.083, 95% CI = 0.064, 0.106				Yes
Li [127]	2023	College students	Physical activity	IPAQ-SF	Depression	BDI-II	Drive	Behavioral Inhibition/Activation System Scale	B = -.028, 95% CI = -043, -.016	0.08	-0.33	-0.08	Yes
Li [127]	2023	College students	Physical activity	IPAQ-SF	Depression	BDI-II	Reward responsiveness	Behavioral Inhibition/Activation System Scale	B = -.004, 95% CI = -.011, 0	0.07	-0.06	-0.08	No
Li [127]	2023	College students	Physical activity	IPAQ-SF	Depression	BDI-II	Fun seeking	Behavioral Inhibition/Activation System Scale	B = .005, 95% CI = .001, .011	0.06	0.07	-0.08	Yes
Chu [39]	2023	Breast cancer survivors	Walking	IPAQ-L	Depression	Chinese version of the Hospital Anxiety and Depression Scale (HADS)	Posttraumatic Growth	Posttraumatic Growth Inventory-Short Form	Standardized estimate = −0.08, 95% CI = −0.12 to −0.03 (indirect effect)	β = .19, p < .01	β = -.40, p < .001	β = -.14, p < .01	Yes
Chu [39]	2023	Breast cancer survivors	Walking	IPAQ-L	Anxiety	Chinese version of the Hospital Anxiety and Depression Scale (HADS)	Posttraumatic Growth	Posttraumatic Growth Inventory-Short Form	Standardized estimate = −0.06, 95% CI = −0.10 to −0.03 (indirect effect)	β = .19, p < .01	β = -.31, p < .001	β = -.11, p > .05	Yes
Kayani [109]	2021	University students	Physical activity	Self-report	Anxiety	State-Trait Anxiety Inventory (STAI Y-6)	Self enhancement	Short form of SE Strategies scale (SFSESS)	−0.0474 (−0.0918, −0.0058)	β = .275	β = -.172	β = -.176	Yes
McIntyre [150]	2019	Women with chronic illness	Physical activity	Adapted from the Active Australia Survey	Depressive symptoms	CESD-10	Health related quality of life	EQ-5D-3L	Indirect effect = −.0009, 95%CI = −.0012, −.0006, R^2 = .39, F (7,1684) = 153.61, p <.0001				Yes
Zuo [247]	2021	Adults	Physical activity	Physical Activity Rating Scale (PARS-3)	Subjective wellbeing	subjective well-being scale and the satisfaction with life scale	Health values	4 item health values scale	B = .358, CI = .296, .420				Yes
Santino [183]	2020	Community dwelling individuals	Leisure time PA	Leisure-Time Physical Activity Questionnaire	Life satisfaction	Life Satisfaction Questionnaire-11	Loneliness	UCLA Loneliness Scale-3	Indirect effect = .003, 95% bootstrap CI = .0004 to .0062, direct effect = .002, -.001 to .0052	r = –.15, p =.05	r = –.69, p <.001	r = .18, p = .02	Yes
Santino [184]	2022	Community dwelling individuals	Leisure time physical activity	Leisure Time Physical Activity Questionnaire-SCI	Life satisfaction	Life Satisfaction Questionnaire-11	Loneliness	UCLA Loneliness Scale-3	Indirect effect=.003, 95% bootstrap CI=.0004 to .0062, CSI=.113				Yes
Guicciardi [82]	2019	Adults with multiple sclerosis	Leisure-time PA	GLTEQ	Mental health	SF-36 MCS	Self-efficacy related to the management of multiple sclerosis - goal setting	Self-Efficacy for Multiple Sclerosis Scale (SEMS)	PA to symptom management self-efficacy: β = .32, symptom management to MH = ns				No
Guicciardi [82]	2019	Adults with multiple sclerosis	Leisure-time PA	GLTEQ	Mental health	SF-36 MCS	Self-efficacy related to the management of multiple sclerosis - symptom management	Self-Efficacy for Multiple Sclerosis Scale	PA to goal setting self-efficacy: β = .37, goal setting to mental health: β = .61, p < .001				Yes
Pickett [175]	2012	Adults with depression or low mood	Leisure time PA	Leisure-Time Exercise Questionnaire (LTEQ)	Depression	BDI-II	Coping self-efficacy and PA self-efficacy	Stop unpleasant emotions and thoughts subscale of the Coping Self-Efficacy Scale (CSE)	Total indirect effect B = -.36, 95% CI = -.74, .05				No
Pickett [175]	2012	Adults with depression or low mood	Leisure time PA	Leisure-Time Exercise Questionnaire (LTEQ)	Positive affect	PANAS	Coping self-efficacy	Stop unpleasant emotions and thoughts subscale of the Coping Self-Efficacy Scale (CSE)	Total indirect effect B = .43, 95% CI = .19, .70				Yes
Pickett [175]	2012	Adults with depression or low mood	Leisure time PA	Leisure-Time Exercise Questionnaire (LTEQ)	Negative affect	PANAS	Coping self-efficacy and PA self-efficacy	Stop unpleasant emotions and thoughts subscale of the Coping	Total indirect effect B = -.15, 95% CI = -.40, .11				No

								Self-Efficacy Scale (CSE)					
MalekRivan [148]	2021	middle aged and older adults	Physical activity	IPAQ-SF	Psychological distress	GHQ-12	Coping strategies (positive reinterpretation, instrument support, denial, self-blame)	Brief COPE inventory	Total effect = -.1448, p <.05				yes
Dahlstrand [44]	2023	Adolescents	Leisure time PA (VPA)	Accelerometer (ActiGraph GT3X+)	Stress	The 10-item version of the perceived stress scale (PSS10)	Shift persist coping style (tendency to shift oneself in response to stressors)	Positive Thinking scale of the Responses to Stress questionnaire	−.0040 (−.0056, −.0023)	.0144*** (.0085, .0203)	−.2743*** (−.303, −.2456)	−.0022 (−.0061, .0016)	Yes
Liu, M. [142]	2024	Undergraduate students	Physical activity	PARS-3	Psychological distress	DASS-21	Coping styles	Simplified coping style questionnaire (SCSQ)	Indirect effect = −0.0002, 95% CI = −0.0033, 0.0026				No
Baker [11]	2023	Women	Leisure-time physical activity	IPAQ	Depressive symptoms	CES-D 12	Outcome expectancies	adapted from other measures	β = -0.002 (-0.008, 0.004)				No
Baker [11]	2023	Women	Transport physical activity	IPAQ	Depressive symptoms	CES-D 21	Outcome expectancies	adapted from other measures	β = -0.000 (-0.002, 0.002)				No
Baker [11]	2023	Women	Occupational physical activity	IPAQ	Depressive symptoms	CES-D 30	Outcome expectancies	adapted from other measures	β = -0.001, (-0.005, 0.002)				No
Baker [11]	2023	Women	Domestic physical activity	IPAQ	Depressive symptoms	CES-D 39	Outcome expectancies	adapted from other measures	β = -0.001 (-0.003, 0.001)				No
Dong [49]	2022	College students	Physical activity	IPAQ	Trait anxiety	State-trait Anxiety Inventory (STAI)	shifting function	more-odd shifting task	Indirect effect β = −0.034, *p* <.01, CI = −0.071, −0.00				Yes
Li [126]	2021	Adolescents	Exercise	Physical Exercise Questionnaire	Psychological distress	Depression Anxiety Stress Scale-21	Learning burnout	Learning Burnout Questionnaire	Indirect effect β = −0.096, CI = −0.115 to −0.077				Yes

SOCIAL MEDIATORS

Baker [11]	2023	Women	Leisure-time physical activity	IPAQ	Depressive symptoms	CES-D 15	Social context of PA	adapted from other measures	β = -0.008 (-0.018, 0.002)				No
Baker [11]	2023	Women	Transport physical activity	IPAQ	Depressive symptoms	CES-D 24	Social context of PA	adapted from other measures	β = -0.001 (-0.004, 0.001)				No
Baker [11]	2023	Women	Occupational physical activity	IPAQ	Depressive symptoms	CES-D 33	Social context of PA	adapted from other measures	β = 0.003 (-0.001, 0.008)				No
Baker [11]	2023	Women	Domestic physical activity	IPAQ	Depressive symptoms	CES-D 42	Social context of PA	adapted from other measures	β = 0.001 (-0.001, 0.004)				No
Taliaferro [195]	2013	Undergraduate students	Sport Participation	Two items assessing the number of sports teams	Depression	CES-D 20	Social support	25 item Personal Resource Questionnaire	F (2, 447) = 111.53, *p* < .001, AR2 = .33, Indirect effect: β = -.57, *p* < .001	β = .15, *p* < .001	β = -.58, *p* <.001	β = -.11, *p* < .05	Yes
Yoshikawa [227]	2016	Employees	Exercise Frequency	One item – In the last six months, how often did you do relatively hard exercise for more than 20 min?	Depression	CES-D	Social support	Social Support Questionnaire	bias-corrected and accelerated confidence interval (BCACI) = −.61 to −.0350, 95% confidence interval (CI) and SSQ Satisfaction score (BCACI) = −.92 to −.18	SSQ Number score: B = 0.60, SE = .28, *p* = .03, SSQ Satisfaction score: B = .29, SE = .11, *p* < .01	SSQ Number score: B = .60, SE = .28, *p* = .03, SSQ Satisfaction score: B = −1.63, SE = .26, *p* < .01	B = −.17, SE = .72, *p* = .81	Yes
Zhang, J. [238]	2022	College students	Physical activity	Physical Activity Scale	Depression	Self-Rating Depression Scale	social support	Social Support Comprehension Scale	β = -.07, CI = -.10, -.03				Yes
Zheng [242]	2024		Physical activity	Physical Activity Readiness Scale	Anxiety	Self-Rating Anxiety Scale	Social support	Social Support Rating Scale	Indirect effect = -.23, 95% CI = -.43, -.03	β = .14, *p* <.01	β = -.23, *p* <.01	β = -.37, *p* <.01	Yes
Guo [84]	2024	College students	Physical activity	Physical Activity Scale	Life satisfaction	SWLS + PANAS	Social support	Social Support Scale	Indirect effect = 0.128, 95% CI = 0.979, 0.164	β = .386, *p* <.01	β = .332, *p* <.01	β = .369, *p* <.01	Yes
Zhang, X. [237]	2022	Adults	Exercise	Self-report	Subjective wellbeing	Brief Subjective Well-Being Scale for Chinese Citizens	Social network	three Likert self-report questions	Indirect effect = 0.011, CI = 0.004, 0.021				Yes

Werneck (a) [215]	2023	Middle aged and older adults	Physical activity	Self-report	Depressive symptoms	URO-D scale.	Social network quality	self-report Likert					No
Werneck (a) [215]	2023	Middle aged and older adults	Physical activity	Self-report	Depressive symptoms	URO-D scale.	Social network size	self-report Likert	Indirect effect β = -.054, 95% CI = -.008, -.028	β = .050, CI = .026, .075	β = -.013, CI = -.021, -.005	β = -.052, CI = -.078, -.026	Yes
Conley [42]	2020	Children	Social based - physical activities	Sports Activities Involvement Questionnaire (SAIQ)	Depression	Child Behavior Checklist (CBCL)	Social connections	Measured by number of close friends	Indirect effect = − .0003, 95% CI − .001, − .0001, *p* < .001; Direct effect = − .01, 95% CI − .008, − .003, *p* < .001				Yes
Ho [94]	2015	School students	Physical activity	Physical Activity Rating Questionnaire for Children and Youth (PARCY)	Mental health	SF-36 MCS	Social connectedness	Resnick School Connectedness Scale (RSC)	Family connectedness: b = .08, *p* = .06, mediation proportion = 16%, School connectedness: b = .07, *p* = .10, mediation proportion = 15%				Yes
McNeil [152]	2022	Adults	Nature based physical activity	Self-report	Wellbeing	The World Health Organization—5 (WHO-5)	Social connectedness	Social Connectedness Scale Revised (SCS-R	b = 0.07, SE = 0.03, 95% CI = 0.01–0.14, β = 0.04	.12, *p* < .05	.32, *p* < .05	.19, *p* < .05	No
Zhang [239]	2023	Adolescents	Physical activity	Self-report	Subjective wellbeing	World Health Organization Well-Being Index (WHO-5)	School connectedness	5 items from the National Longitudinal Study of Adolescent Health	Indirect effect = 0.083, 95% CI = 0.066, 0.103	β = .155, *p* <.001	β = .254, *p* <.001	β = .114, *p* <.001	Yes
Liu, N. [139]	2023		Sport Participation	"In the past year, did you regularly engage in physical activity in your free time?"	Subjective wellbeing	Self-report	Subjective class identity	Self-report	Indirect effect = 0.009, 95% CI = 0.003, 0.016				Yes
Wu [220]	2024	Older adults	Tai chi	Physical Activity Rating Scale (PARS-3)	Psychological distress	Geriatric Health Questionnaire (GHQ-12)	Social participation	social participation index system	Indirect effect = -0.12, 95% CI = -0.212, -0.005				Yes
Wu [220]	2024	Older adults	Ba duan jin	Physical Activity Rating Scale (PARS-3)	Psychological distress	Geriatric Health Questionnaire (GHQ-12)	Social participation	social participation index system	Indirect effect = -0.049, 95% CI = -0.093, -0.023				Yes
Wu [220]	2024	Older adults	Walking	Physical Activity Rating Scale (PARS-3)	Psychological distress	Geriatric Health Questionnaire (GHQ-12)	Social participation	social participation index system	Indirect effect = -0.057, 95% CI = -0.136, -0.011				Yes
Wu [220]	2024	Older adults	Tai chi	Physical Activity Rating Scale (PARS-3)	Psychological distress	Geriatric Health Questionnaire (GHQ-12)	Social participation	social participation index system	$t = -5.981$, $p < 0.05$				Yes
Wu [220]	2024	Older adults	Ba duan jin	Physical Activity Rating Scale (PARS-3)	Psychological distress	Geriatric Health Questionnaire (GHQ-12)	Social participation	social participation index system	$t = -4.544$, $p < 0.05$				Yes
Wu [220]	2024	Older adults	Walking	Physical Activity Rating Scale (PARS-3)	Psychological distress	Geriatric Health Questionnaire (GHQ-12)	Social participation	social participation index system	$t = -5.481$, $p < 0.05$				Yes
VanKim [204]	2013	Undergraduate college students	Vigorous PA	One item – On how many days did you exercise or participate in PA for at least 20 minutes that made you sweat or breathe hard?	Perceived stress	Cohen Perceived Stress Scale	Socialising	Number of close friends and time spent socialising	B = -.57, *p* <.0001				Yes
VanKim [204]	2013	Undergraduate college students	Vigorous PA	One item – On how many days did you exercise or participate in PA for at least 20 minutes that made you sweat or breathe hard?	Perceived stress	Cohen Perceived Stress Scale	Socialising	Number of close friends and time spent socialising	B = -.80, *p* <.0001				Yes
Feuerhahn [64]	2012	Adults	Exercise after work	Self-reported duration of exercise that evening	Positive affect (in the evening)	6 items from the PANAS positive affect scale	Sense of belonging	Eight items adapted from the Sense of Belonging Instrument	z = 3.08, *p* < .001	*r* = .24, *p* < .01	*r* = .11, *p* < .01	*r* = .22, *p* < .01	Yes
Watt [210]	2022	Psychology university students	Physical activity	Asked how often they undertook physical activity	Psychological distress	Kessler 10 (K10)	Sense of belonging	Community Integration Measure (CIM		β = .022, CI = .015, .029	β = -.502, CI = -.608, -.397	β = -.018, CI = -.026, -.009	Yes
Watt [210]	2022	Psychology university students	Physical activity	Asked how often they undertook physical activity	Depression	Kessler 10 (K10)	Sense of belonging	Community Integration Measure (CIM		β = 0.02, t (393) = 6.12, *p* < .01	β = -0.16, t (388) = -4.32, *p* < .01		Yes
Watt [210]	2022	Psychology university students	Physical activity	Asked how often they undertook physical activity	Anxiety	Kessler 10 (K10)	Sense of belonging	Community Integration Measure (CIM		β = 0.02, t (393) = 6.12, *p* < .01	β = -0.94, t (388) = -3.25, *p* < .01		Yes

Oberle [163]	2019	Elementary and secondary school students	Sport	No. of days in the past week	Life satisfaction	5item Satisfaction with Life Scale-Adapted for Children	Peer belonging	Adapted 3-item version of the Relational Provisional Loneliness Questionnaire	Indirect effect est = .07, *p* = <.001, direct effect = .18, *p* <.001				Yes
Oberle [163]	2019	Elementary and secondary school students	Sport	No. of days in the past week	Depression	Seattle Personality Questionnaire depression subscale	Peer belonging	Adapted 3-item version of the Relational Provisional Loneliness Questionnaire	Indirect effect est = .08, *p* = <.001, direct effect = .19, *p* <.001				Yes
Oberle [163]	2019	Elementary and secondary school students	Sport	No. of days in the past week	Anxiety	Seattle Personality Questionnaire anxiety subscale	Peer belonging	Adapted 3-item version of the Relational Provisional Loneliness Questionnaire	Indirect effect est = .06, *p* = <.001, direct effect = .18, *p* <.001				Yes
Bang [12]	2024	Adolescents	Team sports (school)	Self-report participation groups	Depression	7 self-report symptoms	School belonging	Self-report items	Indirect effect = 0.058***				Yes
Bang [12]	2024	Adolescents	Individual sports (school)	Self-report participation groups	Depression	8 self-report symptoms	School belonging	Self-report items	Indirect effect = 0.009				No
Bang [12]	2024	Adolescents	Team and individual sports (school)	Self-report participation groups	Depression	9 self-report symptoms	School belonging	Self-report items	Indirect effect = 0.053***				Yes
Quarta [178]	2022	Adults	Sport	One item frequency measure	Subjective wellbeing	9-item subjective wellbeing scale	Quality of social relationship	WHOQoL Brief social relationships items	β = .02, 95% CI = .003, .009	β = .07, *p* < .001	β = .40, *p* <.001	β = -.05, *p* > .05	Yes
Hou [97]	2024	Older adults	Physical activity	In the past year, how often have you participated in physical exercise in your free time?	Depression	1-item self-report	Social competence	Self-reported time spent with others	Indirect effect = 0.013, p = .004				Yes
Zhuo [245]	2023	College students	Physical activity	Physical Activity Rating Scale	Depression	Short-version Depression Scale compiled by Andresen et al. (1994)	Interpersonal relationship difficulties	Interpersonal Relationship Comprehensive Diagnostic Scale	Indirect effect = -0.12, 95% CI = -.14, -.09	.11, *p* <.01	-.85, *p* <.01	-.03, *p* <.01	Yes
Watt [210]	2022	Psychology university students	Physical activity	Asked how often they undertook physical activity	Psychological distress	Kessler 10 (K10)	Coach Relationship	Coach Relationship Questionnaire (CRQ)		β = .020, CI = .006, .034	β = -.077, CI = -.137, -.018	β = -.018, CI = -.026, -.009	Yes
Watt [210]	2022	Psychology university students	Physical activity	Asked how often they undertook physical activity	Depression	Kessler 10 (K10)	Coach Relationship	Coach Relationship Questionnaire (CRQ)		ns	ns		No
Watt [210]	2022	Psychology university students	Physical activity	Asked how often they undertook physical activity	Anxiety	Kessler 10 (K10)	Coach Relationship	Coach Relationship Questionnaire (CRQ)		ns	ns		No

BEHAVIOURAL MEDIATORS

Doré [52]	2020	Children	Leisure-time PA	Free-time engagement in PA via a 36-item PA and sport checklist.	Mental health	Mental Health Continuum-Short Form (MHC-SF)	MVPA	2-item self-report measure	Indirect effect β = 1.07, *p* < .05, direct effect β = -.22, *p* > .05				Yes
Levante [124]	2024	University students	Physical activity	In the last months, how often have you done physical activity?	Psychological distress	DASS-21	Time Spent on Leisure Activities	In the last month, how often have you spent time in nature? With friends? With family?	(β = 0.012; Boot LLCI = 0.028; Boot ULCI = 0.001				Yes
Dong [50]	2023	Adolescents	Physical activity	Physical Activity Questionnaire for Children (PAQ-C)	Mental health	Mental Health Inventory of Middle School Students (MMHI-60)	Sleep	Self-report	0.319 × (-0.164) =−0.052				No
Choi [38]	2024	Older adults	Physical activity	ever went walking for exercise (yes or no), and ever spent time on vigorous activities (yes or no)	Psychological distress	Patient Health Questionnaire-4 (PHQ-4)	Sleep	Three questions about sleep	Indirect effect (0.01, 95% CI = [−0.02, 0.09], z = 5.05, p = .67)				No
Mu [159]	2024	College students	Physical activity	Physical activity rating scale (PARS-3)	Psychological distress	Depression anxiety and stress scale (DASS-21)	Sleep	Pittsburgh sleep quality index (PSQI)	Indirect effect = -.408, 95% CI = -.523, -.287	-.044, *p* <.001	.389, *p* <.001	-.018, *p* <.01	Yes
Zhang [232]	2020	College students	Physical activity	IPAQ-SF	Stress	DASS-21	Sleep Quality	Pittsburgh Sleep Quality Index (PSQI)	Indirect effect = .003, *p* = .552				No
Zhang [232]	2020	College students	Physical activity	IPAQ-SF	Anxiety	DASS-21	Sleep Quality	Pittsburgh Sleep Quality Index (PSQI)	Indirect effect = .002, *p* = .504				No
Zhang [232]	2020	College students	Physical activity	IPAQ-SF	Depression	DASS-21	Sleep Quality	Pittsburgh Sleep Quality Index (PSQI)	Indirect effect = .001, *p* = .536				No

Fontana [66]	2022	teachers	Physical activity	IPAQ-SF	Stress	Perceived Stress Scale (PSS-10)	Sleep quality	Short Pittsburgh Sleep Quality Index	Indirect effects: OR = 0.62; 95%CI = 0.42–0.82; p < 0.001; mediation proportion = 72.1%				Yes
Lin (b) [133]	2022	Undergrad students	Exercise	Self-report	Subjective wellbeing	Satisfaction with Life Scale (SWLS) + PANAS	Sleep quality	Chinese version of the Pittsburgh Sleep Quality Index	Indirect effect = 0.06, 95% CI [0.03, 0.09])				Yes
Barham [13]	2022	Adults	Physical activity change	Single item	Depression	Hospital Anxiety and Depression Scale (HADS-D)	Sleep health	six sleep dimensions including regularity, satisfaction, alertness, timing, efficiency, and duration	Indirect effect: β= −.022, 95% CI [−.036 to−.008]	β = .08, *p* < .05	β = -.27, *p* < .05	β = -.14, *p* < .05	Yes
Lewis [125]	2021	adults	Physical activity	IPAQ	Anxiety symptoms	Generalized Anxiety Disorder 7-item (GAD-7)	Sleep regularity	self-report	Indirect effect β = − 0.002, CI = − 0.008, 0.005				No
Vandendriessche [203]	2019	Children	Physical activity	One item – Over the past 7 days, on how many days were you physically active for a total of at least 60 min per day?”	Psychological distress	Three items from the Health Behaviour in School-Aged Children (HBSC) Symptom Check List (HBSC-SCL)	Sleep duration - weekdays	When do you usually go to bed? When do you usually wake up?	Indirect effect β = .001, *p* < .001, direct effect β = -.03, *p* < .001				Yes
Vandendriessche [203]	2019	Children	Physical activity	One item – Over the past 7 days, on how many days were you physically active for a total of at least 60 min per day?”	Psychological distress	Three items from the Health Behaviour in School-Aged Children (HBSC) Symptom Check List (HBSC-SCL)	Sleep duration - weekends	When do you usually go to bed? When do you usually wake up?	Indirect effect β = .001, *p* < .001, direct effect β = -.03, *p* < .001				Yes
Leahy [123]	2023	Adolescents	School PA	Intervention versus control	Perceived stress	10-item perceived stress scale	Sleep duration (weekday)	Participants reported the time they usually went to bed and woke up on school	Indirect effect = 0.04, 95% CI = -0.10, 0.18				No
Leahy [123]	2023	Adolescents	School PA	Intervention versus control	Perceived stress	10-item perceived stress scale	Sleep duration (weekend)	Participants reported the time they usually went to bed and woke up on school	Indirect effect = -0.06, 95% CI = −0.23, 0.11				No
Leahy [123]	2023	Adolescents	School PA	Intervention versus control	Perceived stress	10-item perceived stress scale	Sleep latency	In the last two weeks, after you go to bed, how long does it usually take you to fall asleep?	Indirect effect = -0.01, 95% CI = −0.12, 0.11				No
Leahy [123]	2023	Adolescents	School PA	Intervention versus control	Perceived stress	10-item perceived stress scale	Awakenings	In the last two weeks, how many times a night do you usually wake up at night for longer than five minutes?	Indirect effect = -0.03, 95% CI = −0.15, 0.10				No
Leahy [123]	2023	Adolescents	School PA	Intervention versus control	Perceived stress	10-item perceived stress scale	Daytime sleepiness	During your daytime activities, how much of a problem do you have with sleepiness?	Indirect effect = -0.19, 95% CI = −0.44, 0.06				No
Vandendriessche [203]	2019	Children	Physical activity	One item – Over the past 7 days, on how many days were you physically active for a total of at least 60 min per day?”	Psychological distress	Three items from the Health Behaviour in School-Aged Children (HBSC) Symptom Check List (HBSC-SCL)	Sleep difficulties	Sleep item from the HBSC-SCL	Indirect effect β = -.01, *p* < .001, direct effect β = -.02, *p* < .001				Yes
Kaseva [108]	2019	Children and adolescents	Leisure time PA	Five questions assessing the intensity and frequency of leisure-time physical activity	Depressive symptoms	BDI-II	Sleep difficulties	Four items reflecting participants’ experiences of sleep problems	Indirect effect: B = -.00, *p* = .29, direct effect B = .00, *p* = .90	*r* = -.04, ns	*r* = .21, *p* <.01	*r* = -.09, *p* <.01	No
Zhang, B. [236]	2022	University students	Physical activity	IPAQ Chinese version	Psychological distress	General Health Questionnaire 12-item (GHQ-12)	Sleep disorder	Athens Insomnia Scale (CAIS)	Indirect effect β = −0.042, CI = −0.214, −0.056				No
Werneck [212]	2020	Adults	Physical inactivity	Meeting guidelines yes/no	Anxiety	One item - In the period of the pandemic, how often have you felt worried, anxious, or nervous?	Worsening sleep quality	One item – Has the pandemic affected the quality of your sleep?	Indirect effect OR: 1.16, 95% CI = 1.06, 1.26, 21.9% mediated, direct effect OR = 1.68, 95% CI = 1.31, 2.15				Yes
Lewis [125]	2021	adults	Physical activity	IPAQ	Anxiety symptoms	Generalized Anxiety Disorder 7-item (GAD-7)	Insomnia severity	Insomnia Severity Index (ISI)	Indirect effect β = 0.014, CI = − 0.022, 0.050				No
Baker [11]	2023	Women	Leisure-time physical activity	IPAQ	Depressive symptoms	CES-D 17	Sitting time	Adapted from other measures	β = 0.001 (-0.001, 0.003)				No

Baker [11]	2023	Women	Transport physical activity	IPAQ	Depressive symptoms	CES-D 26	Sitting time	Adapted from other measures	β = -0.001 (-0.003, 0.004)				No
Baker [11]	2023	Women	Occupational physical activity	IPAQ	Depressive symptoms	CES-D 35	Sitting time	Adapted from other measures	β = 0.006 (-0.006, 0.018)				No
Baker [11]	2023	Women	Domestic physical activity	IPAQ	Depressive symptoms	CES-D 44	Sitting time	Adapted from other measures	β = 0.001 (-0.003, 0.006)				No
Baker [11]	2023	Women	Leisure-time physical activity	IPAQ	Depressive symptoms	CES-D 18	Screen time	Adapted from other measures	β = 0.000 (-0.001, 0.002)				No
Baker [11]	2023	Women	Transport physical activity	IPAQ	Depressive symptoms	CES-D 27	Screen time	Adapted from other measures	β = -0.001 (-0.004, 0.002)				No
Baker [11]	2023	Women	Occupational physical activity	IPAQ	Depressive symptoms	CES-D 36	Screen time	Adapted from other measures	β = -0.001 (-0.012, 0.009)				No
Baker [11]	2023	Women	Domestic physical activity	IPAQ	Depressive symptoms	CES-D 45	Screen time	Adapted from other measures	β = 0.004 (-0.009, 0.0001)				No
Li [126]	2021	Adolescents	Exercise	Physical Exercise Questionnaire	Psychological distress	Depression Anxiety Stress Scale-21	Problematic phone use	Self-rating Questionnaire for Adolescent Problematic Mobile Phone Use	Indirect effect β = −0.059, CI = −0.077 to −0.044				Yes
Precht [177]	2022	Adults	Physical activity	Liker scale self-report	Mental health	unidimensional Positive Mental Health Scale (PMH-Scale)	Addictive social media use	Brief version of the Bergen Social Media Addiction Scale (BSMAS)		β = -.465, CI = -.854, -.077	β = -.222, CI = -.376, -.068	β = .597, CI = .014, 1.180	Yes
Precht [177]	2022	Adults	Physical activity	Liker scale self-report	Stress	Depression Anxiety Stress Scales 21 (DASS-2)	Addictive social media use	Brief version of the Bergen Social Media Addiction Scale (BSMAS)		β = -.465, CI = -.854, -.077	β = .235, CI = .111, .360	β = -.370, -.843, .102	Yes
Ji [103]	2024	Secondary school students	Physical activity	2 self-report items	Depressive symptoms	CESD	Internet Addiction	Adapted from Young's Diagnostic Questionnaire (YDQ)	Indirect effect = -0.114, 95% CI = -.148, -.089, *p* <.001				Yes
Baker [11]	2023	Women	Leisure-time physical activity	IPAQ	Depressive symptoms	CES-D 16	Fruit and veg intake	Adapted from other measures	β = -0.007 (-0.018, 0.002)				No
Baker [11]	2023	Women	Transport physical activity	IPAQ	Depressive symptoms	CES-D 25	Fruit and veg intake	Adapted from other measures	β = -0.000 (-0.002, 0.004)				No
Baker [11]	2023	Women	Occupational physical activity	IPAQ	Depressive symptoms	CES-D 34	Fruit and veg intake	Adapted from other measures	β = -0.000 (-0.002, 0.002				No
Baker [11]	2023	Women	Domestic physical activity	IPAQ	Depressive symptoms	CES-D 43	Fruit and veg intake	Adapted from other measures	β = 0.000 (-0.002, 0.003)				No
You [230]	2024	Adults	Leisure-time PA	Global Physical Activity Questionnaire	Depressive symptoms	Patient's Health Questionnaire (PHQ-9)	Intake of medium live microbe-containing foods	24 h dietary recall data	Indirect effect = −0.0003 (−0.0005, −0.0001), *p* < 0.001				Yes
You [230]	2024	Adults	Leisure-time PA	Global Physical Activity Questionnaire	Depressive symptoms	Patient's Health Questionnaire (PHQ-9)	Intake of high live microbe-containing foods	24 h dietary recall data	Indirect effect = −0.0001 (−0.00015, −0.00001)				Yes
You [230]	2024	Adults	Leisure-time PA	Global Physical Activity Questionnaire	Depressive symptoms	Patient's Health Questionnaire (PHQ-9)	Intake of low live microbe-containing foods	25 h dietary recall data	βindirect = 0.0001, *p* = 0.216				No
Levante [124]	2024	University students	Physical activity	In the last months, how often have you done physical activity?	Psychological distress	DASS-21	Adherence to the Mediterranean Diet	14-MEDAS questionnaire	β = 0.000; Boot LLCI = 0.020; Boot ULCI = 0.018				No

PHYSIOLOGICAL MEDIATORS

Fitness related	Lindwall [135]	2012	Adults	Physical activity	Adapted version of the widely used four-level scale originally developed by Saltin and Grimby	Depression	Hospital and Anxiety Depression Scale (HADS)	Aerobic fitness	Åstrand indirect test of maximal oxygen uptake (VO2max)	Indirect effect B = -.19, SE = 0.19, 95% CI = 0.54, 0.17, direct effect B = -.89, SE = 0.4, p <.05	B = 3.22, p <.01	B = -.05, ns	B = -.89, p <.05	No
	Lindwall [135]	2012	Adults	Physical activity	Adapted version of the widely used four-level scale originally developed by Saltin and Grimby	Anxiety	Hospital and Anxiety Depression Scale (HADS)	Aerobic fitness	Åstrand indirect test of maximal oxygen uptake (VO2max)	Indirect effect B = .07, SE = .07, 95% CI = -0.04, 0.24, direct effect B = -.84, SE = 0.22, p <.01	B = 3.22, p <.01	B = .02, ns	B = -.84, p < .01	No
	Adams [1]	2018	Testicular cancer survivors	HIIT exercise intervention	Intervention vs control	Δ Mental health	SF-36 MCS	Cardiorespiratory fitness	VO2peak	Indirect effect B = 2.6, CI = 0.2; 5.8, p <.05, direct effect B = 1.1, CI = -3.4; 5.6, p >.05	B = 3.9, CI = 2.5; 5.4, p <.05	B = 0.7, CI = -.003; 1.3, p <.10	B = 1.1, CI = -3.4; 5.6, p >.05	Yes
	Perez-Sousa [171]	2020	Middle aged and older adults with depression	Physical activity	Intervention vs control	Depression	Geriatric Depression Scale (GDS) short form	Physical fitness	Physical fitness test battery including functional reach [16], chair sit-and-reach [17], the 3-metre version of the Timed Up and Go Test (TUG) [18], bi-handgrip strength [19], back scratch [20] and the 6-min walk test along a 20-metre corridor [20].	Indirect effects:<br>Flexibility: β − 0.04 (− 0.07 to − 0.01)<br>back scratch: β − 0.06 (− 0.12 to − 0.02)<br>cardiorespiratory fitness: β − 0.09 (− 0.15 to − 0.04)<br>Direct effect: β − 0.44 (− 0.60 to − 0.28)<br>functional reach, timed Up and go test, handgrip strength, and 6-min walk test were ns indirect effects.				Yes
	Syue [194]	2022	Older adults	Physical activity	IPAQ-SF	Life satisfaction	A single item with an 11-point scale	Functional fitness	A test battery					No
Health and disability related	Backström-Eriksson [9]	2016	Patients with Cystic Fibrosis	Exercise	Retrieved from medical records	Anxiety	Hospital Anxiety and Depression Scale (HADS)	Medical status	Forced expiratory volume, BMI, immunoglobulin G, physical working capacity	Direct effect β = .06, p = .12, indirect effect β = .05, p = .07				No
	Backström-Eriksson [9]	2016	Patients with Cystic Fibrosis	Exercise	Retrieved from medical records	Depression	Hospital Anxiety and Depression Scale (HADS)	Medical status	Forced expiratory volume, BMI, immunoglobulin G, physical working capacity	Direct effect β = .16, p = .17, indirect effect β = .18, p = .11				No
	Liu, N. [139]	2023		Sport Participation	“In the past year, did you regularly engage in physical activity in your free time?”	Subjective wellbeing	self-report	Health	Self-report	Indirect effect = 0.065, 95% CI = 0.042, 0.089	β = .101, p <.001	β = .356, p <.001	β = .099, p <.001	Yes
	Li, B. [129]	2024	College students	Physical activity	IPAQ-SF	Depression	CES-D	Self-rated health	36-item Short Form Health Survey (SF-36)	Indirect pathway was −0.043				Yes
	Mu [159]	2024	College students	Physical activity	Physical activity rating scale (PARS-3)	Psychological distress	Depression anxiety and stress scale (DASS-21)	Self-rated health	Short Form Health Survey-12	Indirect effect = -.829, 95% CI = -.907, -.751	.225, p <.001	-.154, p <.001	-.018, p <.02	Yes
	Levante [124]	2024	University students	Physical activity	In the last months, how often have you done physical activity?	Psychological distress	DASS-21	Self-Reported Physical Health Status	WHOQoL-Bref questionnaire	β = 0.034; Boot LLCI = 0.001; Boot ULCI = 0.072				Yes
	Li [127]	2023	High School students	Physical activity	“Do you participate in physical activity regularly?"	Psychological distress	Symptom Check-List-90	Satisfaction with health	One item from the Child and Adolescent Quality of Life Scale	Indirect effect = −0.059 (low frequency of PA), −0.124 (medium frequency of PA), −0.146 (high frequency of PA)				Yes
	Deng [47]	2018	Middle-aged and older adults	Leisure-time PA	How much time they spent performing activities for exercise, entertainment, job demand, and other purposes	Depressive symptoms	10-item CES-D	Functional health status	Instrumental Activities of Daily Living (IADLs)	Indirect effect: β = -.02, 95% CI =-.03, -.01,<br>direct effect: β = .02, 95% CI = -.03, .06				Yes
	Deng [47]	2018	Middle-aged and older adults	Total physical activity	How much time they spent performing activities for exercise, entertainment, job demand, and other purposes	Depressive symptoms	10-item CES-D	Functional health status	Instrumental Activities of Daily Living (IADLs)	RURAL: indirect effect β = -.07, 95% CI = -.08, -.06, direct effect β = .12, 95% CI = .09, .15<br>URBAN: indirect effect β = -.06, 95% CI = -.07, -.05, direct				Yes

									effect β = .10, 95% CI = .08, .13				
Meadows [153]	2017	Breast cancer survivors	Physical activity	Nine items that assess recreational walking and light, moderate, and vigorous PA using a frequency and duration item forma	Life satisfaction	SWLS	Function	Late-Life Function and Disability Instrument	Indirect effect estimate = .10, $p = .08$) Total indirect effects (fatigue and function) estimate = .161, $p = .012$.	β = -.42, $p < .05$	β = -.25, $p < .05$	β = .14, ns	Yes
Castan [28]	2024	Adults with spinal cord injury	Leisure-time PA	Physical Activity Recall Assessment for People with SCI (PARA-SCI)	Psychological distress	Hospital Anxiety and Depression Scale (HADS-D)	Functionality	Functional Independence Measure		β = .25, $p <.05$	β = -.14, $p >.05$	β = .037, $p >.05$	Yes
Baruth [16]	2016	Adults	Leisure-time PA	Community Health Activities Model Program for Seniors (CHAMPS) questionnaire	Depression	CES-D 10	Disability	Health Assessment Questionnaire (HAQ) Disability Index	Indirect effect: β = .001, $p >.05$, direct effect β =.15, $p >.05$	β = -.07, $p <.05$	β = .55, $p <.05$	β = -.05, $p >.05$	No
Meadows [153]	2017	Breast cancer survivors	Physical activity	Nine items that assess recreational walking and light, moderate, and vigorous PA using a frequency and duration item forma	Life satisfaction	SWLS	Disability	Late-Life Function and Disability Instrument		β = -.02, ns	β = -.12, ns	β = .14, ns	No
Baruth [16]	2016	Adults	Leisure-time PA	Community Health Activities Model Program for Seniors (CHAMPS) questionnaire	Depression	CES-D 10	Stiffness	Participants rated their arthritis symptoms using a Visual Numeric Scale.	Indirect effect β = .02, $p >.05$, direct effect β = .14, $p >.05$	β = -.24, $p >.05$	β = .09, $p <.05$	β = -.07, $p >.05$	No
Baruth [16]	2016	Adults	Leisure-time PA	Community Health Activities Model Program for Seniors (CHAMPS) questionnaire	Depression	CES-D 10	Pain	Participants rated their arthritis symptoms using a Visual Numeric Scale.	Indirect effect β = .02, $p >.05$, direct effect β = .13, $p >.05$	β = -.34, $p <.05$	β = .11, $p <.05$	β = -.05, $p >.05$	No
Barr [14]	2010	Physically active men	Aerobic exercise	Intervention vs control	Affect	Activation Deactivation Adjective Checklist (AD-ACL)	Pain	One item from the Brief Pain Inventory	β = -.44, $p < .05$				Yes
Latimer [120]	2004	Individuals with traumatic spinal cord injury	Exercise	Intervention vs control	Stress	14-item Perceived Stress Scale (PSS)	Pain	Two-item pain subscale from the 36-Item Short-Form Health Survey (SF-36)	F [4,17] = 7.72, $p <.01$	β = .54, $t = 3.04$, $p <.01$	β = .59, $t = 2.99$, p <.05	β = .47, $t = 3.01$, $p <.05$	Yes
Choi [38]	2024	Older adults	Physical activity	ever went walking for exercise (yes or no), and ever spent time on vigorous activities (yes or no)	Psychological distress	Patient Health Questionnaire-4 (PHQ-4)	Pain	Asked if they were bothered by pain in the past month (yes = 1, no = 0).	Indirect effect (−0.06, 95% CI = [−0.09, −0.02], z = −3.18, p = .001)				Yes
Perez-Sousa [172]	2023	Women with Rheumatoid Arthritis	Aquatics exercise	Group allocation to intervention or control	Depression	e Portuguese version of the Beck Depression Inventory-II	Pain	Visual analog scale (VAS)	β: −9.36, 95%CI (−17.68, −2.66)				Yes
Ginis [74]	2003	Individuals with spinal cord injury	Aerobic and resistance training twice weekly	Intervention vs control	Depression	CES-D	Perceived Pain	Two pain items from the Short-Form 36-Item Health Survey (SF-36)	Variance explained by pain $R^2 = .19$, $p < .01$				Yes
Baruth [16]	2016	Adults	Leisure-time PA	Community Health Activities Model Program for Seniors (CHAMPS) questionnaire	Depression	CES-D 10	Fatigue	Participants rated their arthritis symptoms using a Visual Numeric Scale.	Indirect effect β = -.04, $p >.05$, direct effect β =.19, $p <.05$	β = -.20, $p > .05$	β = .15, $p < .05$	β = -.06, $p > .05$	No
Annesi [5]	2019	People participating in Parkinson Disease tailored physical activity classes	Δ Leisure-Time PA	Leisure-Time Physical Activity Questionnaire	Δ Depression	Profile of Mood States depression subscale	Δ Fatigue	Profile of Mood States fatigue subscale	Indirect effect: β = -.02, 95% CI = -.065, -.001 Direct effect: β = -.03, $p = .169$	β = -.09, $p = .01$	β = -.27, $p = .045$	β = -.06, $p = .05$	Yes
Meadows [153]	2017	Breast cancer survivors	Physical activity	Nine items that assess recreational walking and light, moderate, and vigorous PA using a frequency and duration item forma	Life satisfaction	SWLS	Fatigue	Functional Assessment of Cancer Therapy instrument (FACIT	Indirect effect estimate = .06, $p = .09$) Total indirect effects (fatigue and function) estimate = .161, $p = .012$	β = -.23, $p < .05$	β = -.21, $p < .05$	β = .14, ns	Yes
Roppolo [180]	2013	Women with multiple sclerosis	Aerobic and Strength training	Intervention vs control	Depression	BDI	Fatigue	Modified Fatigue Impact Scale (MFIS)	Mediation effect = 4.52, $p <.05$				Yes

Fisher [65]	2017	Women with non-metastatic stage 0-III breast cancer	Physical activity change	Seven-Day Physical Activity Recall Questionnaire	Depressive symptoms	Hamilton Rating Scale for Depression (HRSD)	Fatigue related daily interference	7-item Perceived Interference subscale of the 12-item Fatigue Symptom Inventory (FSI)	Indirect effect β = −.02, *p* = .04	β = -.05, *p* < .01	β = .40, *p* > .05		Yes
Fisher [65]	2017	Women with non-metastatic stage 0-III breast cancer	Physical activity change	Seven-Day Physical Activity Recall Questionnaire	Depressed mood	Depression subscale of the 40-item Affect Balance Scale (ABS)	Fatigue related daily interference	7-item Perceived Interference subscale of the 12-item Fatigue Symptom Inventory (FSI)	Indirect effect β = −.02, *p* = .13	β = -.05, *p* < .01	β = .50, *p* < .01		No
Condello [41]	2016	Adults	Physical activity	AAHPERD (American Alliance for Health, Physical Education, Recreation, and Dance) exercise/medical history questionnaire	Mental health	SF-12 MCS	BMI	Height and weight derived	Indirect effects: PA to BMI = 3.50, *p* < .01, BMI to BDI = 0.97, *p* <.01, BDI to MCS = 0.20, *p* = .03; Direct effect = 4.97, *p* = .04				Yes
Eddolls [55]	2018	Children	Vigorous PA	Actigraph accelerometer	Depressive symptoms	Center for Epidemiologic Studies Depression Scale for Children (CES-DC)	BMI	Stature and body mass were measured, using a stadiometer and body mass scale	Indirect effect: β = − 0.03, *p* < .001	β = − .29, *p* < .01	β = .12, *p* < .05	β = − .03, *p* < .001	Yes
Werneck [213]	2022	Adults	Leisure time physical activity	Self-report	Well-being	Warwick-Edinburgh scale	BMI		0.003 (0.001 to 0.005)*				Yes
Werneck [213]	2022	Adults	Leisure time physical activity	Self-report	Psychological distress	Malaise Inventory	BMI		−0.001 (−0.002 to 0.001)				no
Meyer [155]	2021	Teenagers	Physical activity	Over the past 7 days, on how many days were you physically active for a total of at least 60 min per day?	Life satisfaction	Single item – where on the ladder do you feel you stand at the moment?"	Weight perception - underweight	One item - "Do you think your body is ...?" much too thin, a bit too thin, about right, a bit too fat, much too fat?	Indirect effect: b = .004, 95% MC CI = .0035, .0045	*r* = -.04, *p* < .001	*r* = -.10, *p* < .001	*r* = .15, *p* < .001	Yes
Meyer [155]	2021	Teenagers	Physical activity	Over the past 7 days, on how many days were you physically active for a total of at least 60 min per day?	Life satisfaction	Single item – where on the ladder do you feel you stand at the moment?"	Weight perception - overweight	One item - "Do you think your body is ...?" much too thin, a bit too thin, about right, a bit too fat, much too fat?	Indirect effect: b = .010, 95% MC CI = .0090, .011	*r* = -.11, *p* < .001	*r* = -.22, *p* < .001	*r* = .15, *p* < .001	Yes
Quarta [178]	2022	Adults	Sport	One item frequency measure	subjective wellbeing	9-item subjective wellbeing scale	Energy levels	three items adapted from the Lee's Fatigue scale	β = .09, 95% CI = .058, .129	β = .21, *p* < .001	β = .64, *p* <.001	β = -.05, *p* > .05	Yes
Zou [246]	2023	Adults	Physical activity	Global Physical Activity Questionnaire	Depressive symptoms	9-item Patient Health Questionnaire	Glycemic control	Blood sample	*p* = .48				No

NEUROBIOLOGICAL MEDIATORS

Booij [22]	2014	Adolescents	Exercise	Self-reported hours per week across a range of exercise types at Time 2	Depression	Affective Problem Scale at time 3	HPA axis functioning	AUCgCort	Indirect effect B = -.00, CI = -.02, .00				No
Booij [22]	2014	Adolescents	Exercise	Self-reported hours per week across a range of exercise types at Time 2	Depression	Affective Problem Scale at time 3	Cortisol reactivity	AUCiCort	Indirect effect B = .01, CI = -.00, .04				No
Booij [22]	2014	Adolescents	Exercise	Self-reported hours per week across a range of exercise types at Time 2	Depression	Affective Problem Scale at time 3	Heart rate during social stress	AUCgHR	Indirect effect B = -.03, CI = -.07, -.01				Yes
Booij [22]	2014	Adolescents	Exercise	Self-reported hours per week across a range of exercise types at Time 2	Depression	Affective Problem Scale at time 3	Heart rate reactivity	AUCiHR	Indirect effect B = -.00, CI = -.02, .00				No
Booij [22]	2014	Adolescents	Exercise	Self-reported hours per week across a range of exercise types at Time 2	Depression	Affective Problem Scale at time 3	High sensitivity C-reactive protein	HsCRP	Indirect effect B = .00				No
Olson [164]	2017	Adults with major depressive disorder	Aerobic exercise	Intervention vs control	Depressive symptoms	BDI-II	Changes in N2 TFSF amplitude	EEG	Indirect effect B = -3.72, 95% CI = -12.02, 0.91				No
Huang [99]	2022	Older diabetes patients	Physical activity	Self-report	Depression	DSM-IV	Inflammation	High-sensitivity C-reactive protein (HsCRP)	*z* = −2.01, *p* = 0.045				Yes
You [229]	2022	Adults	Leisure time physical activity	Self-report	Depressive symptoms	Patient Health Questionnaire-9 (PHQ9)	inflammatory potential of the diet	dietary inflammatory index (D	Direct effect = -.0076, *p* <.001, Indirect effect = -.0003, *p* = .046				Yes

Huang [98]	2021	Adults	Physical activity	Self-reported against guidelines	Depression	Patient Health Questionnaire-9 (PHQ-9)	Triglycerides		Mediation effect = 0.06(0.01,0.10), *p* =.014, proportion attributable = 6.28%				Yes
Gujral [83]	2024	Older adults	Physical activity	self-reported and calculated as METs	Psychological distress	DASS	APOE ε4 carrier status	TaqMan genotyping assays on DNA extracted from whole blood samples					Yes
Gujral [83]	2024	Older adults	Physical activity	self-reported and calculated as METs	Psychological distress	DASS	BDNF Val66Met polymorphism	TaqMan genotyping assays on DNA extracted from whole blood samples					No
Donyaei [51]	2023	Women with type 2 diabetes	Exercise training program	Group allocation to intervention or control	Depression	BDI	BDNF	Blood sample	Indirect effect *B* = −57, p = 04	b = 1.10, *p* = .001	b = -.57, *p* = .04	b=-11.25, *p* = .0006	Yes
**COGNITIVE MEDIATORS**													
Birch [20]	2016	Older adults	Physical activity	Rapid Assessment of Physical Activity (RAPA 1)	Depression	CES-D	Cognitive functioning	Montreal Cognitive Assessment (MoCA)	*p* = .04	*r* = .15, *p* = .006	*r* = .15, *p* < .001	*r* = -.23, *p* < .001	Yes
Werneck [213]	2022	Adults	Leisure time physical activity	Self-report	well-being	Warwick-Edinburgh scale	Cognition	vocabulary test	0.000 (−0.001 to 0.001)				no
Werneck [213]	2022	Adults	Leisure time physical activity	Self-report	Psychological distress	Malaise Inventory	Cognition	vocabulary test	0.000 (−0.002 to 0.002)				no
Dong [49]	2022	College students	Physical activity	IPAQ	Trait anxiety	State-trait Anxiety Inventory (STAI)	inhibition function	Stroop task	Indirect effect β = −0.018, *p* <.05, CI = -0.047, 0				Yes
**ENVIRONMENTAL MEDIATORS**													
Jenkins [102]	2022	Adults	Nature-based PA	IPAQ-SF	Psychological wellbeing	Questionnaire for Eudaimonic Wellbeing (QWEB)	Nature relatedness	Nature Relatedness Scale		β = .36, *p* < .05	β = .22, *p* < .05	β = -.05, *p* > .05	Yes
Jenkins [102]	2022	Adults	Nature-based PA	IPAQ-SF	Hedonic Wellbeing	WHO-5	Nature relatedness	Nature Relatedness Scale		β = .36, *p* <.05	β = .08, *p* > .05	β = .04, *p* > .05	Yes
Quarta [178]	2022	Adults	Sport	One item frequency measure	subjective wellbeing	9-item subjective wellbeing scale	Time spent in nature	During the last month, how often did you spend time in nature?	β = .007, 95% CI = -.001, .015	β = .22, *p* < .001	β = .04, *p* > .05	β = -.05, *p* > .05	No
McNeil [151]	2022	Adults	Nature based physical activity	Self-report	Wellbeing	The World Health Organization—5 (WHO-5;	Nature connectedness	Nature Relatedness Scale (NRS)	b = 0.03, SE = 0.03, 95% CI = -0.02 to 0.05, β = 0.01.	.30, *p* < .05	.05, *p* >. 05	.19, *p* < .05	Yes
McNeil [151]	2022	Adults	Nature based physical activity	Self-report	Wellbeing	The World Health Organization—5 (WHO-5;	Nature connectedness + social connectedness	Nature Relatedness Scale (NRS) + Social Connectedness Scale Revised (SCS-R					Yes

Note: CESD = Center for Epidemiologic Studies Depression Scale; DASS = Depression Anxiety Stress Scale; GHQ = General Health Questionnaire; GLTEQ = Godin Leisure-time Exercise Questionnaire; HADS = Hospital Anxiety and Depression Scale; IPAQ = International Physical Activity Questionnaire; IPAQSF = International Physical Activity Questionnaire Short Form; PA = physical activity, PANAS = Positive and Negative Affect Schedule; MVPA = Moderate to Vigorous Physical Activity; Only the first author is listed for brevity; the numbers in parentheses following the first authors' names refer to the individual study IDs of the 247 included studies.

Table S3

Moderators of the relationship between physical activity and mental health.

	First Author	Year	Participants	Physical activity		Mental health		Moderator		Moderation Results	Sig. mod
				Variable	Measure	Variable	Measure	Variable	Measure		
**PSYCHOLOGICAL MODERATORS**											
**Affect, wellbeing, stress, and depression or anxiety symptomology or sensitivity**	Brière [25]	2018	Adolescents	Sport participation	One item – Do you regularly take part in an organised sport?	Depression	CES-D	Pre-existing depressive symptoms	CES-D	β = - .04, p < .05	Yes
	Wichers [218]	2012	Female twins	Daily Life Physical Activity	Experience Sampling Method	Positive affect	PANAS	Depression Risk	Structured Clinical Interview for Diagnostic and Statistical Manual for Mental Health (DSM)–IV axis I disorders	B = 0.05, p = .254, Bt = 10.07, p = .138, Bt = 20.13, p = .012).	Yes
	Annesi [4]	2018	Adolescent candidates for Bariatric Surgery	Leisure-time PA	Leisure-Time Physical Activity Questionnaire	Negative mood	Profile of Mood States-Brief Form (depression, fatigue, tension, vigour, anger, and confusion subscales)	Depressive symptoms	Quick Inventory of Depressive Symptomology (QIDS)	Interaction term β = .01, p = .45	No
	Walsh [206]	2023	Young adults	Physical activity	Actigraph accelerometer	Depression	BDI-II	Risk of bipolar spectrum disorder	Structured clinical diagnostic interview		Yes
	Walsh [205]	2022	Young adults	Physical activity	Actigraph accelerometer	Depressive symptoms	BDI-II	Bipolar disorder	Psychiatric diagnoses (DSM-IV-TR and RDC)	B = − 0.145, SE = 0.066, p = .002	Yes
	Walsh [205]	2022	Young adults	Physical activity	Actigraph accelerometer	Depressive symptoms	BDI-II	Bipolar disorder	Psychiatric diagnoses (DSM-IV-TR and RDC)		No
	Lin (a) [132]	2022	University students	Sport	Sport Participation Level Scale	Depression	Depression Level Scale	Flow experiences	Flow experience scale	β = -.02, p >.05	No
	Johnson [106]	2022	University students	Exercise	Single bout of aerobic exercise at a self-selected intensity compared to studying for class (control)	Positive affect	PANAS	Positive affect	PANAS	Group × Time, F(1, 78) = 7.08, p ≤ .01, ηp2 = 0.08	Yes
**Global self-esteem, self-worth, body image, and appearance**	Han [90]	2023	College students	Physical activity	IPAQ-SF	Depression	Self-rating Depression Scale (SDS)	Body image	Body Image Questionnaire (BIQ)	β = .17, CI =-.05, .389	No
	Han [90]	2023	College students	Physical activity	IPAQ-SF	Anxiety	Self-rating Anxiety Scale (SAS)	Body image	Body Image Questionnaire (BIQ)	β = .270, CI = .054, .485	Yes

**Esteem and confidence in relation to physical activity**	Cho [37]	2017	College students	Weekend physical activity	Number of weekend hours spent in physical activities	Negative affect	Negative affect subscale from PANAS	Mastery	Four types of weekend recovery experiences using Sonnentag and Fritz's (2007) scale	γ = 0.03, SE = 0.03, p > 0.05	No
**Personality**	Chan [32]	2018	Older adults	Leisure-time PA	Godin–Shephard Leisure-Time Physical Activity Questionnaire (GSLTPAQ)	Subjective wellbeing (i.e., life satisfaction and affect)	SWLS and PANAS	Extraversion	The Big Five Inventory	Interaction β = .14, p < .001	Yes
	Giacobbi [72]	2006	Individuals with physical disabilities with experience playing wheelchair sports	Leisure time PA	Leisure-Time Exercise Questionnaire (LTEQ)	Positive affect	PANAS	Extroversion	NEO-Five Factor Inventory	Coef = -.00, t = -.01, p >.05	No
	Giacobbi [72]	2006	Individuals with physical disabilities with experience playing wheelchair sports	Leisure time PA	Leisure-Time Exercise Questionnaire (LTEQ)	Negative affect	PANAS	Extroversion	NEO-Five Factor Inventory	Coef = .00, t = .52, p >.05	No
	Wang [207]	2020	Middle school students	Sport Participation	Extracurricular sports subscale of the Middle School Student School Sports Assessment Scale	Life satisfaction	The Satisfaction with Life Scale for Chinese Adolescents	Extraversion	Extraversion subscale of the NEO Personality Inventory	High extraversion: b simple slope = -.32, p < .001<br>Low extraversion: b simple slope = -.15, p < .01	Yes
	Chan [33]	2024	Older adults	Physical activity	one -item measure	Subjective wellbeing	Warwick-Edinburgh Mental Wellbeing Scale (WEMWBS)	Extraversion	The 50-item International Personality Item Pool	β = -.02, p >.05	No
	Chan [32]	2018	Older adults	Leisure-time PA	Godin–Shephard Leisure-Time Physical Activity Questionnaire (GSLTPAQ)	Subjective wellbeing (i.e., life satisfaction and affect)	SWLS and PANAS	Agreeableness	The Big Five Inventory	Interaction β = .02, p > .05	No
	Giacobbi [72]	2006	Individuals with physical disabilities with experience playing wheelchair sports	Leisure time PA	Leisure-Time Exercise Questionnaire (LTEQ)	Positive affect	PANAS	Agreeableness	NEO-Five Factor Inventory	Coef = -.01, t = -2.02, p <.05	Yes
	Giacobbi [72]	2006	Individuals with physical disabilities with experience playing wheelchair sports	Leisure time PA	Leisure-Time Exercise Questionnaire (LTEQ)	Negative affect	PANAS	Agreeableness	NEO-Five Factor Inventory	Coef = .01, t = 1.78, p >.05	No
	Chan [33]	2024	Older adults	Physical activity	One -item measure	Subjective wellbeing	Warwick-Edinburgh Mental Wellbeing Scale (WEMWBS)	Agreeableness	The 50-item International Personality Item Pool	β = -.02, p >.06	No
	Chan [32]	2018	Older adults	Leisure-time PA	Godin–Shephard Leisure-Time Physical Activity Questionnaire (GSLTPAQ)	Subjective wellbeing (i.e., life satisfaction and affect)	SWLS and PANAS	Conscientiousness	The Big Five Inventory	Interaction β = .08, p >.05	No
	Giacobbi [72]	2006	Individuals with physical disabilities with experience playing wheelchair sports	Leisure time PA	Leisure-Time Exercise Questionnaire (LTEQ)	Positive affect	PANAS	Conscientiousness	NEO-Five Factor Inventory	Coef = -.01, t = -2.16, p < .05	Yes
	Giacobbi [72]	2006	Individuals with physical disabilities with experience playing wheelchair sports	Leisure time PA	Leisure-Time Exercise Questionnaire (LTEQ)	Negative affect	PANAS	Conscientiousness	NEO-Five Factor Inventory	Coef = .00, t = .41, p >.05	No

	Chan [32]	2018	Older adults	Leisure-time PA	Godin–Shephard Leisure-Time Physical Activity Questionnaire (GSLTPAQ)	Subjective wellbeing (i.e., life satisfaction and affect)	SWLS and PANAS	Neuroticism	The Big Five Inventory	Interaction β = -.03, *p* > .05	No
	Giacobbi [72]	2006	Individuals with physical disabilities with experience playing wheelchair sports	Leisure time PA	Leisure-Time Exercise Questionnaire (LTEQ)	Positive affect	PANAS	Neuroticism	NEO-Five Factor Inventory	Coef = .01, *t* = 3.27, *p* < .01	Yes
	Giacobbi [72]	2006	Individuals with physical disabilities with experience playing wheelchair sports	Leisure time PA	Leisure-Time Exercise Questionnaire (LTEQ)	Negative affect	PANAS	Neuroticism	NEO-Five Factor Inventory	Coef = -.01, *t* = -3.66, *p* < .01	Yes
	Chan [32]	2018	Older adults	Leisure-time PA	Godin–Shephard Leisure-Time Physical Activity Questionnaire (GSLTPAQ)	Subjective wellbeing (i.e., life satisfaction and affect)	SWLS and PANAS	Openness to experience	The Big Five Inventory	Interaction β = .16, *p* < .001	Yes
	Giacobbi [72]	2006	Individuals with physical disabilities with experience playing wheelchair sports	Leisure time PA	Leisure-Time Exercise Questionnaire (LTEQ)	Positive affect	PANAS	Openness	NEO-Five Factor Inventory	Coef = -.00, *t* = -.80, *p* >.05	No
	Giacobbi [72]	2006	Individuals with physical disabilities with experience playing wheelchair sports	Leisure time PA	Leisure-Time Exercise Questionnaire (LTEQ)	Negative affect	PANAS	Openness	NEO-Five Factor Inventory	Coef = -.00, *t* = -.37, *p* >.05	No
	Cho [37]	2017	College students	Weekend physical activity	Number of weekend hours spent in physical activities	Negative affect	Negative affect subscale from PANAS	Control	Four types of weekend recovery experiences using Sonnentag and Fritz's (2007) scale	γ = 0.02, SE = 0.06, *p* > 0.05 for control	No
	Annesi [4]	2018	Adolescent candidates for Bariatric Surgery	Leisure-time PA	Leisure-Time Physical Activity Questionnaire	Negative mood	Profile of Mood States-Brief Form (depression, fatigue, tension, vigour, anger, and confusion subscales)	Preoccupation with food	Modified Trait and State Food-Cravings Questionnaire (trait version) preoccupation with food subscale (MTFCQ-PR)	Interaction term β = -.01, *p* = .35	No
	Annesi [4]	2018	Adolescent candidates for Bariatric Surgery	Leisure-time PA	Leisure-Time Physical Activity Questionnaire	Negative mood	Profile of Mood States-Brief Form (depression, fatigue, tension, vigour, anger, and confusion subscales)	Emotional cravings	Modified Trait and State Food-Cravings Questionnaire (trait version) emotional cravings subscale (MTFCQ-EM)	Interaction term β = .00, *p* = .48	No
Psychological factors related to physical activity	White [216]	2018	Adolescents	Leisure-time PA (MVPA)	Adapted version of the WHO Health Behavior in School-aged Children	Positive Affect	Short version of the PANAS-C	Motivation	BREQ-2		No
	White [216]	2018	Adolescents	Leisure-time PA (MVPA)	Adapted version of the WHO Health Behavior in School-aged Children	Negative Affect	Short version of the PANAS-C	Motivation	BREQ-2	β = −.03, *p* < .05	Yes
	White [216]	2018	Adolescents	Leisure-time PA (MVPA)	Actigraph accelerometer	Positive Affect	Short version of the PANAS-C	Motivation	BREQ-2		No
	White [216]	2018	Adolescents	Leisure-time PA (MVPA)	Actigraph accelerometer	Negative Affect	Short version of the PANAS-C	Motivation	BREQ-2	β = −.003, *p* < .001	Yes
	White [216]	2018	Adolescents	Active travel PA (MVPA)	48-h recall travel diary	Positive Affect	Short version of the PANAS-C	Motivation	MATSS		No
	White [216]	2018	Adolescents	Active travel PA (MVPA)	48-h recall travel diary	Negative Affect	Short version of the PANAS-C	Motivation	MATSS		No
	White [216]	2018	Adolescents	Active travel PA (MVPA)	Actigraph accelerometer	Positive Affect	Short version of the PANAS-C	Motivation	MATSS	β = .09, *p* < .05	Yes
	White [216]	2018	Adolescents	Active travel PA (MVPA)	Actigraph accelerometer	Negative Affect	Short version of the PANAS-C	Motivation	MATSS	β = −.01, *p* < .001	Yes
	Lau [121]	2023	Community dwelling stroke survivors	Physical activity	Accelerometer	Affect (positive affect)	Adapted from validated measures	Motivation	Adapted from BREQ-3	Aut mot = .01 (−0.09 0.21), Con mot = −0.02 (−0.20 0.06)	No
	Lau [121]	2023	Community dwelling stroke survivors	Physical activity	Accelerometer	Affect (depressed affect)	adapted from validated measures	Motivation	Adapted from BREQ-2	Aut mot = .02 (−0.03, 0.15), Con mot = −0.02 (−0.10, 0.06)	No

Shin [188]	2014	Active college students	Exercise	Cycle ergometer and running lab based	Affect	Feeling Scale	Intrinsic motivation	Four items taken from the Intrinsic Motivation Inventory	Standardized factor loading of the path from intrinsic motivation to the slopes was statistically significant (FL = 0.47, *p* = .04)	Yes
Solberg [191]	2014	Older adults	Exercise	Intervention vs control	Life Satisfaction	5-item SWLS	Autonomous motivation towards exercise	15-item BREQ	ES = -.23, ±.4 (sig level not determined)	Yes
Solberg [191]	2014	Older adults	Exercise	Intervention vs control	Positive affect	12-item version of the PANAS	Autonomous motivation towards exercise	15-item BREQ	ES = .12, ±.56 (sig level not determined)	No
Solberg [191]	2014	Older adults	Exercise	Intervention vs control	Negative affect	12-item version of the PANAS	Autonomous motivation towards exercise	15-item BREQ	ES = -.30, ±.49 (sig level not determined)	No
Solberg [191]	2014	Older adults	Exercise	Intervention vs control	Life Satisfaction	12-item version of the PANAS	Introjected regulation towards exercise	15-item BREQ	ES = .17. ±.41 (sig level not determined)	No
Solberg [191]	2014	Older adults	Exercise	Intervention vs control	Positive affect	12-item version of the PANAS	Introjected regulation towards exercise	15-item BREQ	ES = .28, ±.50 (sig level not determined)	No
Solberg [191]	2014	Older adults	Exercise	Intervention vs control	Negative affect	12-item version of the PANAS	Introjected regulation towards exercise	15-item BREQ	ES = -.23, ±.52 (sig level not determined)	No
Solberg [191]	2014	Older adults	Exercise	Intervention vs control	Life Satisfaction	12-item version of the PANAS	External regulation towards exercise	15-item BREQ	ES = .31. ±.41 (sig level not determined)	Yes
Solberg [191]	2014	Older adults	Exercise	Intervention vs control	Positive affect	12-item version of the PANAS	External regulation towards exercise	15-item BREQ	ES = -.05, ±.54 (sig level not determined)	Yes
Solberg [191]	2014	Older adults	Exercise	Intervention vs control	Negative affect	12-item version of the PANAS	External regulation towards exercise	15-item BREQ	ES = -.35. ±.49 (sig level not determined)	Yes
Guérin [81]	2013	Female runners	Preferred exercise	30-minute acute bout of exercise	Positive affect	PANAS	Situational motivation - intrinsic motivation	Situational Motivation Scale (SIMS)	High activation positive affect: F (1,39) = .23, *p* = .34, η2 = .01. Low activation positive affect: ns	No
Guérin [81]	2013	Female runners	Preferred exercise	30-minute acute bout of exercise	Positive affect	PANAS	Situational motivation - identified regulation	Situational Motivation Scale (SIMS)	High activation positive affect: F (1,39) = .92, *p* = .34, η2 = .02 Low activation positive affect: ns	No
Guérin [81]	2013	Female runners	Preferred exercise	30-minute acute bout of exercise	Positive affect	PANAS	Situational motivation - introjected regulation	Situational Motivation Scale (SIMS)	High activation positive affect: F (1, 39) = 4.71, *p* < .05, η2 = .11 Low activation positive affect: ns	Yes
Toups [202]	2017	Adults with major depressive disorder	Exercise intervention	High versus low dose group	Depression symptoms	Quick Inventory of Depression Symptomatology (QIDS)	Motivation and energy	Motivation and Energy Inventory	*p* = .015	Yes

	Gerber [71]	2014	Physically active vocational students	Vigorous PA	Two items from the International Physical Activity Questionnaire	Stress	Adolescent Stress Questionnaire	Exercise self-regulation	Three items assessed the degree to which adolescents form exercise-related plans during leisure time	Cross sectional: low exercise self-regulation β = -.14, $p < .05$, moderate exercise self-regulation β = -.13, $p < .05$, high exercise self-regulation β = -.01, $p > .05$, Longitudinal: low exercise self-regulation β = .02, moderate exercise self-regulation β = .02, high exercise self-regulation β = .03 (all $p > .05$)	Yes
**Other psychological factors**	Maher [147]	2021	Undergraduate students	Physical activity	IPAQ	Positive affect	PANAS	Stressful life events	Social Readjustment Rating Scale	B = 0.00003, ns	No
	Maher [147]	2021	Undergraduate students	Physical activity	IPAQ	Positive affect	PANAS	Stressful life events	Social Readjustment Rating Scale	B = 0.00001, ns	No
	Wen [211]	2018	Children	Physical activity (MVPA)	Actigraph accelerometer	Positive affect	PANAS-C	Mother's perceived stress	Perceived Stress Scale	Within person MVPA 60-mins before recording affect x Mother's stress β = -.01, $p < .05$, 30-mins before β = -.02, $p > .05$	Yes
	Wen [211]	2018	Children	Physical activity (MVPA)	Actigraph accelerometer	Negative affect	PANAS-C	Mother's perceived stress	Perceived Stress Scale	Within person MVPA 60-mins before recording affect x Mother's stress β = .01, $p > .05$, 30-mins before β = .01, $p > .05$	No
	Cho [37]	2017	College students	Weekend physical activity	Number of weekend hours spent in physical activities	Negative affect	Negative affect subscale from PANAS	Psychological detachment	Four types of weekend recovery experiences using Sonnentag and Fritz's (2007) scale	γ = −0.09, SE = 0.03, $p < 0.01$	Yes
	Cho [37]	2017	College students	Weekend physical activity	Number of weekend hours spent in physical activities	Negative affect	Negative affect subscale from PANAS	Relaxation	Four types of weekend recovery experiences using Sonnentag and Fritz's (2007) scale	γ = 0.03, SE = 0.04, $p > 0.05$	No
	Klussman [111]	2021	Adults	Physical activity	IPAQ	Depression	Patient Health Questionnaire (PHQ-4)	Self-connection	12-item Self-Connection Scale	F (3, 109) = 2.00, p =.12, ηp = .05	Yes
	Klussman [111]	2021	Adults	Physical activity	IPAQ	Anxiety	Patient Health Questionnaire (PHQ-4)	Self-connection	12-item Self-Connection Scale	F (3, 109) = 2.26, p =.09, ηp =.06	No
	Klussman [111]	2021	Adults	Physical activity	IPAQ	Positive affect	PANAS	Self-connection	12-item Self-Connection Scale	ns	No
	Klussman [111]	2021	Adults	Physical activity	IPAQ	Negative affect	PANAS	Self-connection	12-item Self-Connection Scale	F (3, 114) = 1.04, p =.38, ηp =.03.	No
	Klussman [111]	2021	Adults	Physical activity	IPAQ	Life satisfaction	Single validated item – In general, how satisfied are you with your life?	Self-connection	12-item Self-Connection Scale	Not reported as ns	No
	Liu, R. [140]	2024	Adults	Physical activity	Self-report Likert scale	Mental health	adapted 5-point Likert scale	Quality of life	Adapted 5-point Likert scale	Interaction effect = −0.054, p = .164	No
	Gyasi [86]	2024	Older adults from low and middle income	physical activity	IPAQ-SF	Depression	CES-D 10	Loneliness	UCLA Loneliness Scale	b = -0.045, 95% CI = -.064, -.025	Yes
	Borges [23]	2022	Smokers	Exercise	Rating of Perceived Exertion during the one-mile Rockport treadmill walk test	Mood	National Institute of Mental Health self-rating scale	Distress tolerance	perceived ability to tolerate distressing affective states	(b = −0.21, SE = 0.07, CI95% = −0.35, −0.06, p < 0.01	Yes
	Borges [23]	2022	Smokers	Exercise	Rating of Perceived Exertion during the one-mile Rockport treadmill walk test	Anxiety	National Institute of Mental Health self-rating scale	Distress tolerance	perceived ability to tolerate distressing affective states	(R2 = 0.02, F(5,187) = 0.81, p = 0.55)	No
	Xin [222]	2023	Smokers	Exercise	Self-report	Anxiety	generalized anxiety disorder 7-item (GAD-7)	Concern about COVID-19	Likert scale self-report		Yes

**SOCIAL MODERATORS**										
Dunton [54]	2015	Adults	Momentary activity level	During each EMA question sequence, participants were asked to indicate their current activity level	Positive affect	items were selected to represent activated (happy, cheerful) and deactivated (calm or relaxed) pleasure	Social context (alone vs with another)	Participants were asked to answer either "yes" or "no" to indicate whether they were alone	β = .30, SE = .13, *p* = .024	Yes
Dunton [54]	2015	Adults	Momentary activity level	During each EMA question sequence, participants were asked to indicate their current activity level	Negative affect	items represented combinations of activated (nervous or anxious, stressed) and deactivated (sad or depressed, frustrated, or angry	Social context (alone vs with another)	Participants were asked to answer either "yes" or "no" to indicate whether they were alone		No
Johansson [105]	2011	University students	Walking	20-minute walk	Anxiety/Depression	Anxiety/Depression subscale from the Negative Mood Scale	Social context	With or without a friend	F (1,18) = 0.36, *p* = 556	No
Gyasi [85]	2019	Older adults	Physical activity	General Physical Activity Questionnaire (GPAQ)	Psychological distress	K10	Social support	Frequency of contact with family/friends, frequency of social events	OR = .65, *p* < .005	Yes
Moya [158]	2021	Older adults	Physical activity	IPAQ	Depressive symptoms	Five-item Geriatric Depression Scale (GDS-5)	Social support	Tilburg Frailty Indicator (TFI)	*p* < .05	Yes
Xiong [223]	2023	Older adults	Physical activity	The Physical Activity Rating Scale (PARS-3)	Depression	Center for Streaming Depression Scale (CES-D)	Social support	Social Support Rating Scale (SSRS)	t = −9.144, *p* < 0.01	Yes
Gyasi [86]	2024	Older adults from low and middle income	physical activity	IPAQ-SF	Depression	CES-D 10	Social isolation	Social Network Index	b = -0.02, 95% CI = -.030, -.011	Yes
Brady [24]	2021	Rheumatoid arthritis patients	Physical activity	National Institutes of Health American Association of Retired Persons (NIH-AARP) Diet and Health Study questionnaire	Anxiety	HADS	Covid isolation	Yes/No	Light PA: β = -.11, 95% CI = -.32, .09<br>Walking: β = .04, 95% CI = -.17, .25<br>Exercise: β = -.08, 95% CI = -.30, .15	No
Brady [24]	2021	Rheumatoid arthritis patients	Physical activity	National Institutes of Health American Association of Retired Persons (NIH-AARP) Diet and Health Study questionnaire	Depression	HADS	Covid isolation	Yes/No	Light PA: β = -.10, 95% CI = -.30, .10<br>Walking β = .05, 95% CI = -.15, .26<br>Exercise β = -.06, 95% CI = -.28, .16	No
**BEHAVIOURAL MODERATORS**										
Asztalos [7]	2015	Adults	Physical activity	IPAQ	Psychological distress	General Health Questionnaire	Sitting Time	Hours	β = -.08, *p* = .019	Yes
Asztalos [7]	2015	Adults	Physical activity	IPAQ	Depression	Symptom Check List depression subscale	Sitting Time	Hours	β = -.10, *p* = .004	Yes
Asztalos [7]	2015	Adults	Physical activity	IPAQ	Anxiety	Symptom Check List anxiety subscale	Sitting Time	Hours	β = -.07, *p* = .038	Yes
Cho [37]	2017	College students	Weekend physical activity	Number of weekend hours spent in physical activities	Negative affect	Negative affect subscale from PANAS	Sleep	Number of hours slept on Saturday and Sunday nights	γ=−0.02, SE=0.01, *p* <.05	Yes
Alfini [3]	2020	Older adults	Acute exercise	Intervention vs control	Positive Affect	PANAS	Sleep Disturbance	Actigraph + sleep diary	β = −.08, 95% CI = −.14 to −.02, *p* = .005	Yes
Gyasi [86]	2024	Older adults from low and middle income	physical activity	IPAQ-SF	Depression	CES-D 10	Sleep problems	Self-report	b = -0.02, 95% CI = -.030, -.010	Yes
Mumba [160]	2021	Adults	Physical activity	Self-report	Psychological distress	Self-report	Alcohol consumption	Self-report	F (4, 160453) = 727.05, *p* < .001	Yes
Mumba [160]	2021	Adults	Physical activity	Self-report	Psychological distress	Self-report	Smoking	Self-report	F (4, 282366) = 1109.07, *p* < .001	Yes
Chang [34]	2020	Adults	Exercise	One question on exercise frequency	Positive Mood	Profile of Mood States (POMS)	Exercise frequency pre-covid	One question on how often people exercised before the pandemic	Exercise during pandemic:<br>β = .14, *p* = .004<br>Exercise pre-pandemic x exercise during pandemic:<br>β =-.48, *p* = .01 (for those in lower pre pandemic exercise category)<br>β = .33, *p* = .02 (for those in higher pre	Yes

										pandemic exercise category)	
	Laurier [122]	2021	Adolescents	Sport	Self-report	Anxiety	Psychological Distress Index	Sport participation prior to lockdown	Self-report	Interaction effect = -0.33, *p* >.05	No
	Laurier [122]	2021	Adolescents	Sport	Self-report	Depression	Psychological Distress Index	Sport participation prior to lockdown	Self-report	Interaction effect = -1.93, *p* <.01	Yes
**PHYSIOLOGICAL MODERATORS**											
**Physical health and disability related**	Giacobbi [72]	2006	Individuals with physical disabilities who have played wheelchair sports	Leisure time PA	Leisure-Time Exercise Questionnaire (LTEQ)	Positive affect	PANAS	Years disabled	Years	Coef = -.00, *t* = -.88, *p* >.05	No
	Giacobbi [72]	2006	Individuals with physical disabilities who have played wheelchair sports	Leisure time PA	Leisure-Time Exercise Questionnaire (LTEQ)	Negative affect	PANAS	Years disabled	Years	Coef = .00, *t* = .65, *p* >.05	No
	Bhandari [18]	2020	Older adults	Physical activity	Self-reported engagement in physical activities	Anxiety	General Health Questionnaire – anxiety items	Disability	Ability to see, hear, walk, chew, speak and remember	β = .004, *p* = .727	No
	Bhandari [18]	2020	Older adults	Physical activity	Self-reported engagement in physical activities	Depression	General Health Questionnaire – depression items	Disability	Ability to see, hear, walk, chew, speak and remember	β = -.02, *p* = .129	No
	Fessler [63]	2023	Adults	Physical activity	2 self-report items	Psychological wellbeing	CASP-19	Multimorbidity	Self-reported chronic conditions	b = 0.36 (0.28; 0.44)***	Yes
	Fessler [63]	2023	Adults	Physical activity	2 self-report items	Depression	EURO-D scale	Multimorbidity	Self-reported chronic conditions	b = 0.28 (0.31; 0.25)***	Yes
	Bhandari [18]	2020	Older adults	Physical activity	Self-reported engagement in physical activities	Anxiety	General Health Questionnaire – anxiety items	Functionality	Number of active daily living activities needing assistance	β = .04, *p* = .086	No
	Bhandari [18]	2020	Older adults	Physical activity	Self-reported engagement in physical activities	Depression	General Health Questionnaire – depression items	Functionality	Number of active daily living activities needing assistance	β = .02, *p* = .269	No
	Gyasi [86]	2024	Older adults from low and middle income	physical activity	IPAQ-SF	Depression	CES-D 10	Functional limitations	Medical Outcomes Study Short Form-36	b = -0.041, 95% CI = -.057, -.026	Yes
	Gyasi [86]	2024	Older adults from low and middle income	physical activity	IPAQ-SF	Depression	CES-D 10	Pain inference	Medical Outcomes Study Short Form-36	b = -0.022, 95% CI = -.033, -.013	Yes
	Gyasi [86]	2024	Older adults from low and middle income	physical activity	IPAQ-SF	Depression	CES-D 10	Pain severity	Medical Outcomes Study Short Form-36	b = -0.016, 95% CI = -.025, -.008	Yes
	Kong [112]	2022	Older adults	Physical activity	Self-report	Depressive symptoms	K-GDS-SF	Lower body muscle strength	5 times sit-to-stand test (5STST)	β = .3407, 95%CI = .1190, .5625	Yes
	Bhandari [18]	2020	Older adults	Physical activity	Self-reported engagement in physical activities	Depression	General Health Questionnaire – depression items	Non-communicable diseases	Self-reported presence of non-communicable diseases	β = -.11, *p* <.001	Yes
	Bhandari [18]	2020	Older adults	Physical activity	Self-reported engagement in physical activities	Anxiety	General Health Questionnaire – anxiety items	Non-communicable diseases	Self-reported presence of non-communicable diseases	β = -.15, *p* <.001	No
	Smith [190]	2021	Weight discordant siblings	Within person physical activity	Actigraph accelerometer	Negative affect	EMA reported current sad, frustrated, angry, and/or stress	BMI	Height and weight were measured with an electronic scale and digital stadiometer	Between participants B = -.02, *p* = .637, within participants B = -.02, *p* = .01	Yes
	Smith [190]	2021	Weight discordant siblings	Between person physical activity	Actigraph accelerometer	Negative affect	EMA reported current sad, frustrated, angry, and/or stress	BMI	Height and weight were measured with an electronic scale and digital stadiometer	Between participants B = -.09, *p* = .661, within participants B = -.04, *p* = .071	No
	Rutherford [181]	2022	Adults	Total physical activity	Global Physical Activity Questionnaire (GPAQ)	Depression	Patient Health Questionnaire (PHQ-9)	BMI	weight in kilograms divided by height in meters squared	*p* = 0.814	No
	Han [90]	2023	College students	Physical activity	IPAQ-SF	Depression	Self-rating Depression Scale (SDS)	BMI		β = .009, CI = -.558, .577	no
	Han [90]	2023	College students	Physical activity	IPAQ-SF	Anxiety	Self-rating Anxiety Scale (SAS)	BMI		β = -.183, CI = -.750, .384	no
	Annesi [4]	2018	Adolescent candidates for Bariatric Surgery	Leisure-time PA	Leisure-Time Physical Activity Questionnaire	Negative mood	Profile of Mood States-Brief Form (depression, fatigue, tension, vigour, anger, and confusion subscales)	Impact of Weight on Quality of Life	Impact of Weight on Quality of Life-Kids (IWQOL)	Interaction term β = .01, *p* = .20	No

**NEUROBIOLOGICAL MODERATORS**										
Dotson [53]	2016	Older adults	Physical activity	Intervention vs control	Depression	CES-D	BDNF	DNA blood test	F (1, 347) = 3.09, *p* = .079	Yes
Ku [117]	2017	Adults	Exercise	One item - "Did you usually engage in physical exercise?"	Depressive symptoms	Chinese version of the CES-D	Apolipoprotein E type 4 allele (APOE-e4)	Genotypes were groups as APOE-e4 carrier and APOE-e4 non-carrier determined via blood sample	The interaction between APOE-e4 carrier status and exercise was also significant (*p* = .046). Compared to high active non carriers (RR = 1.00), high active APOE-e4 carriers had higher risk of depression (RR = 1.66, *p* <.01)	Yes
Petruzzello [173]	1997	University students	Cycling	Submaximal workload tests	State anxiety	State Anxiety Inventory (SAI) short form	Left versus right activated	EEG activity was recorded from the left and right midfrontal (F3. F4) and parietal (P3, P4) regions.	Group x Time interaction F (2.4.28.8) = 4.07, *p* = .028. E = .48.	Yes
Huang [98]	2021	Adults	Physical activity	Self-reported against guidelines	Depression	Patient Health Questionnaire-9 (PHQ-9)	Triglycerides		Interaction effect = 0.17(-0.03,0.36), p =.09, proportion attributable = 18.97%	No
**COGNITIVE MODERATORS**										
Birch [20]	2016	Older adults	Physical activity	Rapid Assessment of Physical Activity (RAPA 1)	Depression	CES-D	Cognitive functioning	Montreal Cognitive Assessment (MoCA)	OR = 0.991, 95% CI [0.985, 0.997], *p* = .005	Yes
**ENVIRONMENTAL MODERATORS**										
Geniole [70]	2016	Young adults	Walking	Experimental (all participants undertook a walk and reported affect after)	Affect	The Affect Grid	Location	Urban vs natural	Location by Time interaction: F [1,30] = 6.38, *p* = .017, Zp =.18	Yes
Dunton [54]	2015	Adults	Momentary activity level	During each EMA question sequence, participants were asked to indicate their current activity level	Positive affect	items were selected to represent activated (happy, cheerful) and deactivated (calm or relaxed) pleasure	Location (indoors vs outdoors)	Participants were asked “where were you just before the beep went off?”		No
Dunton [54]	2015	Adults	Momentary activity level	During each EMA question sequence, participants were asked to indicate their current activity level	Negative affect	Items represented activated (nervous or anxious, stressed) and deactivated (sad or depressed, frustrated, or angry) affect	Location (indoors vs outdoors)	Participants were asked “where were you just before the beep went off?”	β = .206, SE = .097, *p* = .034	Yes
Bodin [21]	2001	Regular runners	Running	All participants engaged in running and reported measures post run	Depression/Anxiety	Anxiety/Depression subscale from the Negative Mood Scale	Environment	Park versus urban	F (1,10) = 0.93, *p* = .36	No
Johansson [105]	2011	University students	Walking	20-minute walk	Anxiety/Depression	Anxiety/Depression subscale from the Negative Mood Scale	Environment	Street versus park	F (1,18) = 1.30, *p* = .270	No
Fauth [60]	2007	Youth	Sport	Self-reported participation in sport	Anxious and depressive symptoms	Child Behavior Checklist (CBCL)	Prescence of neighbourhood resources for youth	Neighbourhood-level measures— both drawn from the Community Survey	Sports x resources: β = .05, *p* > .05	No
Fauth [60]	2007	Youth	Sport	Self-reported participation in sport	Anxious and depressive symptoms	Child Behavior Checklist (CBCL)	Perceived neighbourhood violence	Neighbourhood-level measures— both drawn from the Community Survey	Sports x violence: β = .00, *p* > .05	No
Asiamah [6]	2022	Full-time African academics	Physical activity	IPAQ-SF	Mental health	9-item standard scale	Neighbourhood walkability	NEWS-A (the Australian version of the Neighborhood Environment Walkability Scale)		Yes
**INDIVIDUAL DEMOGRAPHIC MODERATORS**										
Brière [25]	2018	Adolescents	Sport participation	One item "Do you regularly take part in an organized sport?"	Depression	CES-D	Age			No
Wassink-Vossen [209]	2018	Patients with major depressive disorder	Physical activity	IPAQ	Depression	Inventory of Depressive Symptomatology (IDS)	Age		Interaction between PA and age: B = .11, *p* = .015, stratified results <60 years	No

								B = 2.03, p = .022, >60 years B = 2.24, p = .128	
Gomez-Baya [77]	2017	Adolescents	Sport participation	One item - ‘How often do you practice some sports or gymnastics during out-of-school time?’	Depressive symptoms	Children’s Depression Inventory	Age	not reported	No
Hogan [95]	2013	Adults	Single bout of moderate exercise	Intervention vs control	Low arousal positive affect	Modified version of the emotion sampler used by Carstensen and colleagues (2011)	Age	b* = 0.55, p = .028, ΔR2 = .03	Yes
Hogan [95]	2013	Adults	Single bout of moderate exercise	Intervention vs control	High arousal positive affect	Modified version of the emotion sampler used by Carstensen and colleagues (2011)	Age	b* = 0.45, p = .046; ΔR* = .02	Yes
Lindwall [134]	2011	Older adults	Physical activity	“Frequency of moderate physical activity"	Depression	Adapted version of the EURO-D scale	Age	Δ chi square = 106.37	Yes
Maher [146]	2015	Adults	Physical activity	Modified version of the Godin Leisure Time Exercise Questionnaire (GLTEQ)	Life satisfaction	Single item from the SWLS	Age	Age x usual physical activity: Parameter estimate =.12, p <.05; Age x daily physical activity: Parameter estimate = -.01, ns	Yes
Lopes [144]	2023	Older adolescents and adults	Domestic physical activity	Telephone interview questions adapted from IPAQ	Depressive symptoms	Patient Health Questionnaire-9 (PHQ-9	Age		Yes
Lopes [144]	2023	Older adolescents and adults	Transport physical activity	Telephone interview questions adapted from IPAQ	Depressive symptoms	Patient Health Questionnaire-9 (PHQ-10	Age		Yes
Lopes [144]	2023	Older adolescents and adults	Leisure time physical activity	Telephone interview questions adapted from IPAQ	Depressive symptoms	Patient Health Questionnaire-9 (PHQ-11	Age		Yes
Lopes [144]	2023	Older adolescents and adults	Occupational physical activity	Telephone interview questions adapted from IPAQ	Depressive symptoms	Patient Health Questionnaire-9 (PHQ-12	Age		No
Brière [25]	2018	Adolescents	Sport participation	One item "Do you regularly take part in an organized sport?"	Depression	CES-D	Sex		No
Costigan [43]	2019	High School students	Vigorous PA	Actigraph accelerometer	Positive Affect	PANAS-C	Sex		No
Costigan [43]	2019	High School students	Vigorous PA	Actigraph accelerometer	Negative affect	PANAS-C	Sex	β = -.038, SE = .02	Yes
Dotson [53]	2016	Older adults	Physical activity	Intervention vs control	Depression	CES-D	Sex	F (1, 347) = 4.13, p = .043,	Yes
Smith [189]	2018	Adolescents	Resistance training	Intervention vs control	Subjective wellbeing	Psychological flourishing scale	Sex	p = >.10	No
Gianotta [73]	2023	Adolescents	Vigorous PA	How often do you exercise at least 30 min in your spare time so that you get tired/ sweaty?”	Depression	Depression Self-Rating Scale Adolescent version, DSRS-A	Sex	Constrained model: CFI = .96, RMSEA = .06, free model: CFI = .96, RMSEA = .06	No
Evans [59]	2017	Physically active healthy adults	Exercise	Exercise diary	Depressed mood	A subset of items from the original 14 items developed by King, Taylor, Haskell, and DeBusk (1989)	Gender	Exercise duration x gender β = .16, p < .05, effect for males β = -.20, p < .05, effect for females β = -.03, p > .05	Yes
Evans [59]	2017	Physically active healthy adults	Exercise	Exercise diary	Anxiety	A subset of items from the original 14 items developed by King, Taylor, Haskell, and DeBusk (1989)	Gender	Exercise duration x gender β = .18, p < .01 Effect for males β = -.24, p < .05, Effect for females β = -.06, p > .05	Yes
Evans [59]	2017	Physically active healthy adults	Exercise intensity	Exercise diary	Depressed mood	A subset of items from the original 14 items developed	Gender	Exercise intensity x gender β = .24, p < .05	Yes

						by King, Taylor, Haskell, and DeBusk (1989)		Effect for males β = -.33, *p* < .05,<br>Effect for females β = -.09, *p* > .05	
Evans [59]	2017	Physically active healthy adults	Exercise intensity	Exercise diary	Anxiety	A subset of items from the original 14 items developed by King, Taylor, Haskell, and DeBusk (1989)	Gender	Exercise duration x gender *p* > .05	No
Tiggelman [200]	2014	Adolescents with asthma	Habitual PA	Self-reported time spent engaging in activities	Anxiety and Depression (One construct)	HADS	Gender	Total group: β = -.07, *p* = .06<br>Boys: β = -.07, *p* = .07<br>Girls: β = -.11, *p* = .05<br>X2(1) = 8.34, *p* < .01	Yes
Bartholomew [15]	1998	Undergraduate students in exercise science classes	Resistance training	Intervention vs control	Anxiety	State Trait Anxiety Inventory	Gender	Low intensity: F (5,13) = 3.84, *p* <.05<br>Moderate intensity: F (5,13) = 4.61, *p* < .05<br>High intensity: F (5,13) = 3.92, *p* < .05	Yes
Fredericks [69]	2006	Year 11 students	Sport participation	“Were you a member of any athletic or sports team at school?” and “Were you involved in any organized summer or after school sports or recreational program over the past 12 months?”	Depressive symptoms	Reduced version of the Children’s Depression Inventory	Gender	Sport x gender: F = 0.01, *p* >.05	No
Gomez-Baya [77]	2017	Adolescents	Sport participation	One item - ‘How often do you practice some sports or gymnastics during out-of-school time?’	Depressive symptoms	Children’s Depression Inventory	Gender	not reported	No
Halliday [89]	2019	Adolescents	Physical activity	Physical Activity Questionnaire for Adolescents (PAQ-A)	Depression	DASS-21	Gender	2017 wave: F (1,357) =.05, *p* = .831<br>2016 wave: F (1,461) =.47, *p* = .494<br>2015 wave: F (1,412) =.02, *p* = .883<br>2014 wave: F (1,384) =.32, *p* = .573	No
Halliday [89]	2019	Adolescents	Physical activity	Physical Activity Questionnaire for Adolescents (PAQ-A)	Anxiety	DASS-21	Gender	2017 wave: F (1,357) =1.40, *p* = .238<br>2016 wave: F (1,461) =.12, *p* = .728<br>2015 wave: F (1,412) =.06, *p* = .804<br>2014 wave: F (1,384) =.19, *p* = .665	No
Halliday [89]	2019	Adolescents	Physical activity	Physical Activity Questionnaire for Adolescents (PAQ-A)	Stress	DASS-21	Gender	2017 wave: F (1,357) =.85, *p* = .357<br>2016 wave: F (1,441) =.16, *p* = .687<br>2015 wave: F (1,412) =.04, *p* = .842<br>2014 wave: F (1,384) =.68, *p* = .411	No
Hunt-Shanks [100]	2009	Cardiac patients	Leisure time PA	GLTEQ	Depression	HADS	Gender	F (1, 796) = 4.56, *p* = 0.03	Yes
Hunt-Shanks [100]	2009	Cardiac patients	Leisure time PA	GLTEQ	Anxiety	HADS	Gender	F (1, 796) = 4.58, *p* = 0.03	Yes
Hunt-Shanks [100]	2009	Cardiac patients	Leisure time PA	GLTEQ	Negative affect	HADS	Gender	Not reported as ns	No
Lindwall [134]	2011	Older adults	Physical activity	Frequency of moderate physical activity	Depression	Adapted version of the EURO-D scale	Gender		No

Pan [168]	2022	Early adolescents	Weekday physical exercise	Self-reporting of extra-curricular activities	Depression	Items from the Patient Health Questionnaire (PHQ-9) and the Center for Epidemiological Studies-Depression (CES-D).	Gender		β = .025, p >.05	No
Pan [168]	2022	Early adolescents	Weekend physical exercise	Self-reporting of extra-curricular activities	Depression	Items from the Patient Health Questionnaire (PHQ-9) and the Center for Epidemiological Studies-Depression (CES-D).	Gender		β = .026, p >.05	No
Tian [199]	2022	College students	Physical activity	IPAQ-SF	Anxiety	Generalized Anxiety Disorder Scale (GAD-7)	Gender		male: β = −0.03, p > 0.05, female: β = 0.10, p < 0.05	Yes
Fredericks [69]	2006	Year 11 students	Sport participation	"Were you a member of any athletic or sports team at school?" and "Were you involved in any organized summer or after school sports or recreational program over the past 12 months?"	Depressive symptoms	Reduced version of the Children's Depression Inventory	Race		Sport x race: F = 1.45, p > .05	No
Liu, Y. [141]	2024	Adults	Physical activity	Accelerometer	Depressive symptoms	Patient Health Questionnaire	Race	Self-report	Interaction term: p <.05	Yes
Biese [19]	2024	Adolescent athletes	Sport participation	Self-report - categorical played or did not play	Depression	Patient Health Questionnaire-9 Item (PHQ-9)	Race		b = -1.19 ± 0.6, p = 0.05	Yes
Biese [19]	2024	Adolescent athletes	Sport participation	Self-report - categorical played or did not play	Anxiety	General Anxiety Disorder-7 Item (GAD-7)	Race		Interaction estimate [b] = -1.18 ± 0.6, p = 0.04	Yes
Hayes [91]	1986	Adults	Exercise	Self-reported number of activities	Psychological wellbeing	Eight psychological items from the Langner index (1962)	Income		Exercise x income β = -.19, p < .05	Yes
Smith [189]	2018	Adolescents	Resistance training	Intervention vs control	Subjective wellbeing	Psychological flourishing scale	SES	Socio-Economic Indexes for Areas (SEIFA) Index of Relative Socio-economic Disadvantage (IRSD)	p > .10	No
Liu, Y. [141]	2024	Adults	Physical activity	Accelerometer	Depressive symptoms	Patient Health Questionnaire	SES	Self-report data on household income level, education attainment and occupation	Interaction term: p <.06	Yes
Biese [19]	2024	Adolescent athletes	Sport participation	Self-report - categorical played or did not play	Depression	Patient Health Questionnaire-9 Item (PHQ-9)	SES		b = -1.21 ± 0.6, p = 0.03	Yes
Biese [19]	2024	Adolescent athletes	Sport participation	Self-report - categorical played or did not play	Anxiety	General Anxiety Disorder-7 Item (GAD-7)	SES		b = -1.23 ± 0.5, p = 0.02	Yes
Pan [168]	2022	Early adolescents	Weekday physical exercise	Self-reporting of extra-curricular activities	Depression	Items from the Patient Health Questionnaire (PHQ-9) and the Center for Epidemiological Studies-Depression (CES-D).	Family SES		β = -.018, p >.05	No
Pan [168]	2022	Early adolescents	Weekend physical exercise	Self-reporting of extra-curricular activities	Depression	Items from the Patient Health Questionnaire (PHQ-9) and the Center for Epidemiological Studies-Depression (CES-D).	Family SES		β = -.022, p >.05	No
White [217]	2020	Women	Work-related PA	IPAQ	Psychological distress	GHQ-30	Occupation type	International Standard Classification of Occupations (2008)	F [8, 55] = 2.26, p = .036	Yes

Werneck (b) [215]	2023	Adults	Leisure-time PA	Specific questionnaire based on the Surveillance System for Risk and Protective Factors for Chronic Diseases by Telephone Survey from the Brazilian Ministry of Health	Depression	Patient Health Questionnaire-9	Occupation type	REF = Managers/qualified professionals<br>Technicians/associate professionals = 0.073 (0.024; 0.121) *p* = 0.003<br>Clerks/service workers = 0.028 (-0.014; 0.070) *p* = 0.184<br>Qualified manual workers = 0.067 (0.025; 0.110) p = 0.002<br>Elementary manual workers = 0.065 (0.016; 0.115) *p* = 0.010	Yes
Werneck (b) [215]	2023	Adults	Transport physical activity	Specific questionnaire based on the Surveillance System for Risk and Protective Factors for Chronic Diseases by Telephone Survey from the Brazilian Ministry of Health	Depression	Patient Health Questionnaire-10	Occupation type	REF = Managers/qualified professionals<br>Technicians/associate professionals = -0.004 (-0.048; 0.040) *p* =0.857<br>Clerks/service workers = -0.001 (-0.032; 0.031) *p* =0.971<br>Qualified manual workers = -0.001 (-0.031; 0.028) p =0.930<br>Elementary manual workers = -0.007 (-0.039; 0.025) *p* =0.660	No
Werneck (b) [215]	2023	Adults	Occupational physical activity	Specific questionnaire based on the Surveillance System for Risk and Protective Factors for Chronic Diseases by Telephone Survey from the Brazilian Ministry of Health	Depression	Patient Health Questionnaire-11	Occupation type	REF = Managers/qualified professionals<br>Technicians/associate professionals = -0.002 (-0.013; 0.010) *p* =0.798<br>Clerks/service workers = -0.001 (-0.011; 0.011) p =0.964<br>Qualified manual workers = -0.013 (-0.023; -0.003) *p* =0.013<br>Elementary manual workers = -0.008 (-0.018; 0.003) *p* =0.144	Yes
Werneck (b) [215]	2023	Adults	Household PA	Specific questionnaire based on the Surveillance System for Risk and Protective Factors for Chronic Diseases by Telephone Survey from the Brazilian Ministry of Health	Depression	Patient Health Questionnaire-12	Occupation type	REF = Managers/qualified professionals<br>Technicians/associate professionals = 0.001 (-0.032; 0.033) *p* = 0.974<br>Clerks/service workers = 0.010 (-0.009; 0.029) p = 0.302<br>Qualified manual workers = 0.004 (-0.014; 0.022) *p* = 0.638<br>Elementary manual workers = 0.001 (-0.021; 0.021) *p* = 0.977	No

Meckes [154]	2020	First responders	Physical activity	IPAQ-SF	Psychological distress	DASS-21	First responder role	Traditional or emotional support	Service role X PA: β = .24, *p* = .01	Yes
Berger [17]	1987	College students	Swimming	Intervention vs control	State anxiety	State Trait Anxiety Inventory	Swimming experience	Beginner versus intermediate	F (l, 40) = 4.83, *p* < .04	Yes
Forshaw [68]	2023	University students	Physical activity	Accelerometer	Depressive symptoms	CESD-R scale	Physical activity identity	Five items of the validated Exercise Identity Scale	Interaction β = -.03, 95% CI = -.14, -.03, *p* =.03	Yes
Forshaw [68]	2023	University students	Physical activity	Accelerometer	Depressive symptoms	CESD-R scale	Physical activity identity	Five items of the validated Exercise Identity Scale	Interaction β = -.08, 95% CI = -.14, -.03, *p* <.01	Yes
Miller [157]	2005	Female undergraduate students	Aerobic exercise	20-minute experimental exercise condition	Positive affect	PANAS	Mode preference	Stair stepper, treadmill, rower, cycle ergometer, cross-country	Group (mode preference) × time interaction: F (6, 198) = 5.45, *p* < .001	Yes
Miller [157]	2005	Female undergraduate students	Aerobic exercise	20-minute experimental exercise condition	Negative affect	PANAS	Mode preference	Stair stepper, treadmill, rower, cycle ergometer, cross-country	Group (mode preference) × time interaction, F (3, 198) = 6.57, *p* < .001	No

Note: CESD = Center for Epidemiologic Studies Depression Scale; DASS = Depression Anxiety Stress Scale; GHQ = General Health Questionnaire; GLTEQ = Godin Leisure-time Exercise Questionnaire; HADS = Hospital Anxiety and Depression Scale; IPAQ = International Physical Activity Questionnaire; IPAQSF = International Physical Activity Questionnaire Short Form; PA = physical activity, PANAS = Positive and Negative Affect Schedule; MVPA = Moderate to Vigorous Physical Activity; Only the first author is listed for brevity; the numbers in parentheses following the first authors' names refer to the individual study IDs of the 247 included studies.
