# Supplementary material for: Physical activity and mental health: a systematic review and best-evidence synthesis of mediation and moderation studies
Source: Int J Behav Nutr Phys Act. 2024 Nov 28;21:134. doi: 10.1186/s12966-024-01676-6 (PMC11603721; doi:10.1186/s12966-024-01676-6)
Supplement: Supplementary file 5 — Additional file 5. Risk of Bias Table 14AUG24. Risk of Bias table. Results of the risk of bias assessment for each included study. [file 12966_2024_1676_MOESM5_ESM.pdf]

Table S4

Risk of Bias of individual studies.

Study ID	First Author	Year	Risk of bias criteria							Total
			1	2	3	4	5	6	7	
1	Adams	2018	1	1	1	1	1	1	1	7
2	Aguinaga	2018	1	0	1	1	1	1	1	6
3	Alfini	2020	1	0	1	1	1	1	0	5
4	Annesi	2018	1	0	1	1	1	1	0	5
5	Annesi	2019	1	0	1	1	1	1	0	5
6	Asiamah	2022	1	0	1	1	1	0	1	5
7	Asztalos	2015	0	0	1	1	1	1	1	5
8	Awick	2017	1	0	1	1	1	1	1	6
9	Backström-Eriksson	2016	1	0	0	1	1	1	1	5
10	Bae	2024	1	0	1	1	1	1	0	5
11	Baker	2023	1	0	1	1	0	1	1	5
12	Bang	2024	1	0	0	1	1	1	0	4
13	Barham	2022	1	1	0	1	0	1	1	5
14	Barr	2010	1	0	1	1	0	1	0	4
15	Bartholomew	1998	1	0	1	1	1	1	0	5
16	Baruth	2016	1	0	1	1	0	1	1	5
17	Berger	1987	1	0	0	1	1	1	0	4
18	Bhandari	2020	0	0	0	1	1	1	1	4
19	Biese	2024	1	0	0	1	1	1	1	5
20	Birch	2016	1	0	1	1	0	1	0	4
21	Bodin	2001	1	0	0	1	1	1	0	4
22	Booij	2014	1	0	0	1	1	1	1	5
23	Borges	2022	1	0	1	0	0	1	1	4
24	Brady	2021	1	0	1	1	1	1	1	6
25	Brière	2018	1	0	0	1	1	1	1	5
26	Broman-Fulks	2018	1	0	0	1	1	1	0	4
27	Buffart	2014	1	0	1	1	1	1	1	6
28	Castan	2024	1	1	1	1	1	0	0	5
29	Cecchini	2019	1	0	1	1	1	1	0	5
30	Chae	2016	0	0	1	1	1	1	0	4
31	Chair	2020	1	0	1	1	1	1	0	5
32	Chan	2018	1	0	1	1	1	1	1	6
33	Chan	2024	1	0	0	1	1	1	1	5
34	Chang	2020	0	1	0	1	0	1	1	4
35	Chen	2019	1	0	1	1	1	1	0	5
36	Chen	2022	1	0	0	0	0	1	1	3
37	Cho	2017	1	0	0	1	1	1	0	4
38	Choi	2024	1	0	0	1	0	1	0	3
39	Chu	2023	1	0	1	1	1	1	1	6
40	Clement	2024	1	0	0	1	0	1	1	4
41	Condello	2016	1	0	1	1	1	1	0	5
42	Conley	2020	1	0	1	1	1	1	1	6
43	Costigan	2019	0	0	1	1	1	1	0	4
44	Dahlstrand	2021	1	0	1	1	1	1	1	6
45	Dang	2023	1	0	1	1	1	1	0	5
46	Deng	2016	1	0	0	1	1	1	0	4
47	Deng	2018	1	0	1	1	1	1	0	5
48	Deng	2023	1	1	1	1	1	1	1	7
49	Dong	2022	1	0	1	1	1	1	0	5

Study ID	First Author	Year	Risk of bias criteria							Total
			1	2	3	4	5	6	7	
50	Dong	2023	1	1	1	1	0	1	0	5
51	Donyaei	2023	1	1	1	1	1	1	0	6
52	Doré	2020	0	0	1	1	1	1	1	5
53	Dotson	2016	1	0	1	1	1	1	1	6
54	Dunton	2015	1	0	1	1	1	1	1	6
55	Eddolls	2018	1	0	1	1	1	1	0	5
56	Elavsky (a)	2005	0	0	1	1	1	1	1	5
57	Elavsky (b)	2005	1	0	1	1	1	1	0	5
58	Elavsky	2009	1	0	0	1	1	1	1	5
59	Evans	2017	1	0	0	1	1	1	0	4
60	Fauth	2007	1	0	0	1	1	1	1	5
61	Feng	2024	1	0	1	1	1	1	0	5
62	Fernandes	2024	1	0	0	1	1	1	0	4
63	Fessler	2023	1	0	0	1	1	1	1	5
64	Feuerhahn	2012	0	0	1	1	1	1	0	4
65	Fisher	2017	1	0	1	1	1	1	1	6
66	Fontana	2022	1	0	1	1	1	1	1	6
67	Foroughi	2023	1	1	0	1	1	1	0	5
68	Forshaw	2023	1	1	1	1	1	1	0	6
69	Fredericks	2006	1	0	0	1	0	1	1	4
70	Geniole	2016	1	1	0	1	1	1	1	6
71	Gerber	2014	0	0	1	1	0	1	0	3
72	Giacobbi	2006	1	0	1	1	1	1	0	5
73	Gianotta	2023	1	0	1	1	1	1	1	6
74	Ginis	2003	1	0	0	1	1	1	0	4
75	Ginoux	2021	1	0	1	1	1	1	0	5
76	Goldstein	2020	1	0	1	1	1	1	1	6
77	Gomez-Baya	2017	1	0	0	1	0	1	0	3
78	González-Hernández	2019	1	0	1	1	1	1	0	5
79	Görgülü	2021	1	0	1	1	1	1	0	5
80	Greenleaf	2009	1	0	1	1	1	1	0	5
81	Guérin	2013	1	0	1	1	1	1	1	6
82	Guicciardi	2019	1	0	1	1	1	1	0	5
83	Gujral	2024	1	0	0	1	1	1	0	4
84	Guo	2024	1	0	1	1	1	1	0	5
85	Gyasi	2019	1	0	1	1	0	1	1	5
86	Gyasi	2024	1	0	1	1	1	1	1	6
87	Hachenberger (a)	2022	1	0	1	1	1	1	0	5
88	Hachenberger (b)	2022	1	0	1	1	0	1	0	4
89	Halliday	2019	1	0	1	1	1	1	0	5
90	Han	2023	1	0	1	1	1	1	1	6
91	Hayes	1986	1	0	0	1	0	1	0	3
92	Herring	2021	0	0	1	1	1	1	1	5
93	Herzog	2022	1	1	0	1	1	1	0	5
94	Ho	2015	1	1	1	1	1	1	0	6
95	Hogan	2013	1	0	1	1	1	1	0	5
96	Hogan	2015	1	0	1	1	1	1	0	5
97	Hou	2024	1	0	0	0	0	1	0	2
98	Huang	2021	1	0	1	1	1	1	1	6
99	Huang	2022	1	0	0	1	1	1	1	5
100	Hunt-Shanks	2009	1	0	1	1	1	1	1	6
101	Jenkins	2021	1	1	1	1	1	1	0	6

Study ID	First Author	Year	Risk of bias criteria							Total
			1	2	3	4	5	6	7	
102	Jenkins	2022	1	1	1	1	1	1	0	6
103	Ji	2024	1	0	0	1	1	1	0	4
104	Jia	2023	1	0	1	1	1	1	0	5
105	Johansson	2011	1	0	0	1	1	1	0	4
106	Johnson	2022	1	1	1	0	0	1	0	4
107	Joseph	2013	0	0	1	1	1	1	0	4
108	Kaseva	2019	1	0	1	1	1	1	1	6
109	Kayani	2021	0	0	0	1	1	1	1	4
110	Kikkawa	2023	1	1	1	1	1	1	0	6
111	Klussman	2021	1	0	1	1	1	1	0	5
112	Kong	2022	1	0	0	1	1	1	1	5
113	Konopack	2012	1	0	1	1	1	1	0	5
114	Kozielly	2022	1	0	1	1	1	1	0	5
115	Kratz	2014	1	0	1	1	1	1	0	5
116	Kruk	2019	1	1	1	1	1	1	0	6
117	Ku	2017	1	0	1	1	1	1	1	6
118	Kukihara	2018	1	0	0	1	1	1	0	4
119	La Rocque	2021	1	1	1	1	1	1	1	7
120	Latimer	2004	1	0	0	1	1	1	0	4
121	Lau	2023	1	1	1	1	1	1	1	7
122	Laurier	2021	1	0	0	1	1	1	0	4
123	Leahy	2023	1	0	1	1	0	1	1	5
124	Levante	2024	1	0	0	1	1	1	0	4
125	Lewis	2021	0	0	1	1	0	1	0	3
126	Li	2021	1	0	1	1	1	1	1	6
127	Li	2023	1	1	1	1	1	1	0	6
128	Li, Z.	2024	1	0	0	0	0	1	0	2
129	Li, B.	2024	0	0	1	1	1	1	0	4
130	Li, W.	2024	0	0	0	1	0	0	0	1
131	Liang	2022	1	0	1	1	1	1	1	6
132	Lin (a)	2022	0	0	0	1	1	1	0	3
133	Lin (b)	2022	0	0	0	1	1	1	0	3
134	Lindwall	2011	0	0	0	1	0	1	1	3
135	Lindwall	2012	1	0	0	1	1	1	1	5
136	Liu, S.	2023	1	0	1	1	1	1	0	5
137	Liu, Y.	2023	1	0	1	1	1	1	0	5
138	Liu, M.	2023	0	0	1	1	1	1	0	4
139	Liu, N.	2023	0	0	0	1	1	1	0	3
140	Liu, R.	2024	1	0	0	0	0	1	0	2
141	Liu, Y.	2024	1	0	1	1	1	1	1	6
142	Liu, M.	2024	0	0	1	1	1	1	0	4
143	Liu, X.	2024	1	0	1	1	1	1	1	6
144	Lopes	2023	1	0	1	1	1	1	1	6
145	Mack	2013	0	0	1	1	1	1	0	4
146	Maher	2015	1	0	1	1	1	1	1	6
147	Maher	2021	0	1	1	1	1	1	0	5
148	MalekRivan	2021	1	0	1	1	1	1	1	6
149	Marselle	206	1	0	0	1	1	1	1	5
150	McIntyre	2019	1	0	1	1	1	1	1	6
151	McNeil	2022	1	0	0	1	1	1	0	4
152	McPhie	2012	1	0	0	1	1	1	1	5
153	Meadows	2017	1	0	1	1	1	1	1	6

Study ID	First Author	Year	Risk of bias criteria							Total
			1	2	3	4	5	6	7	
154	Meckes	2020	1	0	1	1	1	1	0	5
155	Meyer	2021	1	0	1	1	0	1	0	4
156	Meyer	2023	0	0	1	1	1	1	0	4
157	Miller	2005	1	0	1	1	1	1	0	5
158	Moya	2021	0	1	1	1	1	1	0	5
159	Mu	2024	0	0	0	0	0	1	0	1
160	Mumba	2021	0	0	0	0	0	1	0	1
161	Mumba	2024	1	1	1	1	1	1	0	6
162	Nezlek	2018	0	0	0	0	0	1	1	2
163	Oberle	2019	1	0	0	1	1	1	0	4
164	Olson	2017	1	1	1	1	1	1	0	6
165	O'Rourke	2023	1	0	1	1	1	1	1	6
166	Oshimi	2022	0	0	0	1	1	1	0	3
167	Pacewicz	2022	1	0	1	1	1	1	0	5
168	Pan	2022	1	0	0	1	1	1	0	4
169	Pascoe	2023	1	0	0	1	1	1	1	5
170	Paxton	2010	0	0	1	1	1	1	1	5
171	Perez-Sousa	2020	1	0	0	1	1	1	1	5
172	Perez-Sousa	2023	0	0	1	1	1	1	1	5
173	Petruzzello	1997	1	0	1	1	1	1	0	5
174	Phillips	2013	1	0	1	1	1	1	0	5
175	Pickett	2012	1	0	1	1	1	1	1	6
176	Precht	2021	1	1	1	1	1	1	1	7
177	Precht	2022	0	0	0	1	1	1	1	4
178	Quarta	2022	0	0	0	1	0	1	1	3
179	Riddervold	2023	1	0	1	1	1	1	0	5
180	Roppolo	2013	1	0	0	1	1	1	0	4
181	Rutherford	2022	1	0	1	1	1	1	1	6
182	Ryan	2008	1	0	1	1	0	1	0	4
183	Santino	2020	0	0	1	1	1	1	1	5
184	Santino	2022	1	0	1	1	1	1	1	6
185	Sato	2016	0	0	1	1	0	1	1	4
186	Shang	2021	1	0	1	1	1	1	0	5
187	Shang	2023	1	0	1	1	1	1	0	5
188	Shin	2014	0	0	0	1	1	1	0	3
189	Smith	2018	1	0	1	1	1	1	1	6
190	Smith	2021	0	0	1	1	1	1	1	5
191	Solberg	2014	1	0	1	1	1	1	1	6
192	Stuntz	2020	1	1	1	1	0	1	1	6
193	Sun	2024	1	0	0	1	1	1	1	5
194	Syue	2022	1	0	1	1	0	1	0	4
195	Taliaferro	2013	0	0	0	1	1	1	1	4
196	Tang	2022	1	0	1	1	1	1	0	5
197	Tao	2022	0	0	0	0	0	1	0	1
198	Theodoropoulou	2017	1	1	1	1	1	1	0	6
199	Tian	2022	1	0	1	1	1	1	0	5
200	Tiggelman	2014	1	0	0	1	1	1	1	5
201	Tihanyi	2015	1	0	0	1	1	1	0	4
202	Toups	2017	1	0	1	1	0	1	1	5
203	Vandendriessche	2019	0	0	0	1	1	1	1	4
204	VanKim	2013	1	0	1	1	1	1	1	6
205	Walsh	2022	1	0	1	1	1	1	0	5

Study ID	First Author	Year	Risk of bias criteria							Total
			1	2	3	4	5	6	7	
206	Walsh	2023	1	0	1	1	0	1	1	5
207	Wang	2020	1	0	1	1	1	1	0	5
208	Wang	2022	0	0	1	1	1	1	1	5
209	Wassink-Vossen	2018	0	0	1	1	1	1	1	5
210	Watt	2022	0	0	0	1	1	1	0	3
211	Wen	2018	1	0	1	1	1	1	1	6
212	Werneck	2020	1	0	0	1	1	1	1	5
213	Werneck	2022	1	0	0	1	0	1	1	4
214	Werneck (a)	2023	0	0	0	0	0	1	1	2
215	Werneck (b)	2023	1	0	1	1	1	1	0	5
216	White	2018	1	1	1	1	1	1	1	7
217	White	2020	1	0	1	1	0	1	1	5
218	Wichers	2012	1	0	1	1	1	1	0	5
219	Wilson	2016	1	0	1	1	1	1	0	5
220	Wu	2024	0	0	1	1	1	1	0	4
221	Wut	2023	1	0	1	1	1	1	0	5
222	Xin	2023	1	0	1	1	1	1	0	5
223	Xiong	2023	1	0	1	1	1	1	0	5
224	Yang	2022	1	0	1	1	1	1	1	6
225	Yao	2022	1	0	0	1	1	1	1	5
226	Yildirim	2023	0	0	1	1	1	1	0	4
227	Yoshikawa	2016	1	0	0	1	1	1	0	4
228	You	2021	1	0	0	1	0	1	1	4
229	You	2022	1	0	1	1	1	1	0	5
230	You	2024	1	0	1	1	1	1	1	6
231	Zeibig	2023	1	1	1	0	0	1	0	4
232	Zhang	2020	1	0	0	1	1	1	0	4
233	Zhang	2021	1	0	1	1	1	1	0	5
234	Zhang, Z.	2022	1	0	0	0	0	1	1	3
235	Zhang, B.	2022	0	0	0	1	0	1	0	2
236	Zhang, J.	2022	0	0	1	1	1	1	0	4
237	Zhang, X. (a)	2022	1	0	1	1	1	1	1	6
238	Zhang, X. (b)	2022	0	0	1	1	1	1	0	4
239	Zhang	2023	1	0	1	1	1	1	1	6
240	Zhao	2021	0	0	1	0	1	1	0	3
241	Zhao	2024	1	1	1	1	1	1	0	6
242	Zheng	2024	1	0	1	1	1	1	0	5
243	Zhou	2023	1	0	1	1	1	1	0	5
244	Zhu	2023	1	0	0	1	1	1	0	4
245	Zhuo	2023	0	0	1	1	1	1	0	4
246	Zou	2023	1	0	1	1	1	1	1	6
247	Zuo	2021	1	0	1	1	0	1	0	4

Note: Criterion 1 = participant selection, Criterion 2 = power calculation, Criterion 3 = valid physical activity measurement, Criterion 4 = valid mental health outcome measurement, Criterion 5 = valid mediator/moderator measure, Criterion 6 = appropriate analysis, Criterion 7 = covariates included in analysis.
